# Broad-Scope Amination of Aryl Sulfamates Catalyzed by a Palladium Phosphine Complex

**DOI:** 10.1021/acscatal.3c03166

**Published:** 2023-08-04

**Authors:** Andrea Monti, Joaquín López-Serrano, Auxiliadora Prieto, M. Carmen Nicasio

**Affiliations:** † Departamento de Química Inorgánica, Universidad de Sevilla, Aptdo 1203, 41071 Sevilla, Spain; ‡ Instituto de Investigaciones Químicas (IIQ), Departamento de Química Inorgánica and Centro de Innovación Química Avanzada (ORFEO-CINQA), Universidad de Sevilla and CSIC, 41092 Sevilla, Spain; § Laboratorio de Catálisis Homogénea, Unidad Asociada al CSIC, CIQSO-Centro de Investigación en Química Sostenible and Departamento de Química, Universidad de Huelva, Campus de El Carmen s/n, 21007 Huelva, Spain

**Keywords:** amination, palladacycle, phosphine, DFT calculations, microkinetic modeling, aryl sulfamates

## Abstract

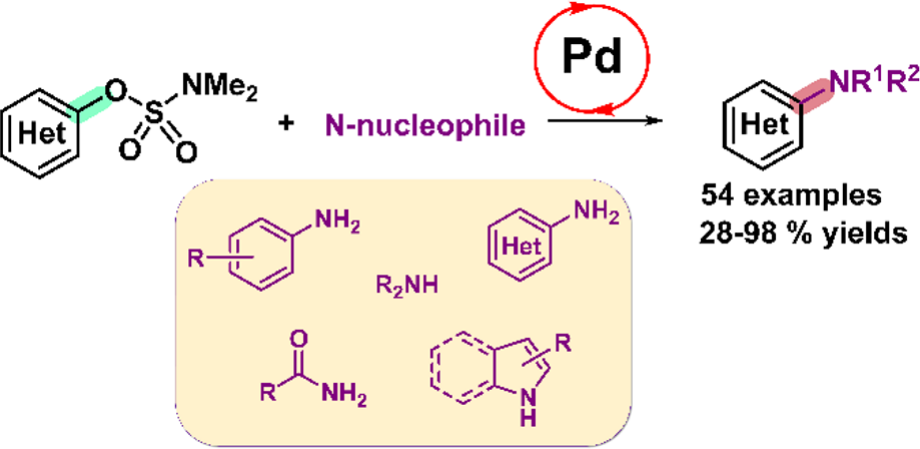

Among phenol-derived electrophiles, aryl sulfamates are attractive substrates since they can be employed as directing groups for C–H functionalization prior to catalysis. However, their use in C–N coupling is limited only to Ni catalysis. Here, we describe a Pd-based catalyst with a broad scope for the amination of aryl sulfamates. We show that the *N*-methyl-2-aminobiphenyl palladacycle supported by the PCyp_2_Ar^Xyl2^ ligand (Cyp = cyclopentyl; Ar^Xyl2^ = 2,6-bis(2,6-dimethylphenyl)phenyl) efficiently catalyzes the C–N coupling of aryl sulfamates with a variety of nitrogen nucleophiles, including anilines, primary and secondary alkyl amines, heteroaryl amines, *N*-heterocycles, and primary amides. DFT calculations support that the oxidative addition of the aryl sulfamate is the rate-determining step. The C–N coupling takes place through a cationic pathway in the polar protic medium.

## Introduction

In recent years, phenol derivatives have gained popularity as viable surrogates for aryl halide electrophiles in transition metal-catalyzed cross-coupling chemistry.^1^ In particular, nickel catalysts allow for the activation and subsequent functionalization of a variety of phenol-derive electrophiles, including less reactive aryl ethers.^2,3^ Despite the tremendous advances made with palladium catalysts in terms of versatility, substrate scope, functional group compatibility, and milder reaction conditions, the use of phenol derivatives is basically limited to more reactive aryl sulfonates (e.g., aryl triflates and tosylates) in Pd-catalyzed reactions.^4^

Unlike sulfonate derivatives, aryl sulfamates are appealing substrates since they can be used as directing groups for C–H functionalization^5^ and show superior stability within the wide range of experimental conditions applied in cross-coupling reactions. Given the ability of Ni to cleave C(sp^2^)–O bonds,^6^ most reported protocols with sulfamates as reaction partners are based on the use of Ni catalysts.^7^ To date, only a few examples with aryl sulfamates as electrophiles in palladium-catalyzed C–C bond formation have been reported.^8^ Of these, the room-temperature Suzuki–Miyaura coupling of aryl sulfamates described by Hazari and co-workers is of particular note.^8c,8d^ The authors ascribe the excellent performance of their catalytic system to the extra stabilization provided by the noncovalent interaction between the Pd center and the biaryl motif of the XPhos ligand.^8d^ This secondary interaction is key to lowering the energy of the transition state for oxidative addition, the turnover-limiting step.

**Figure 1:**
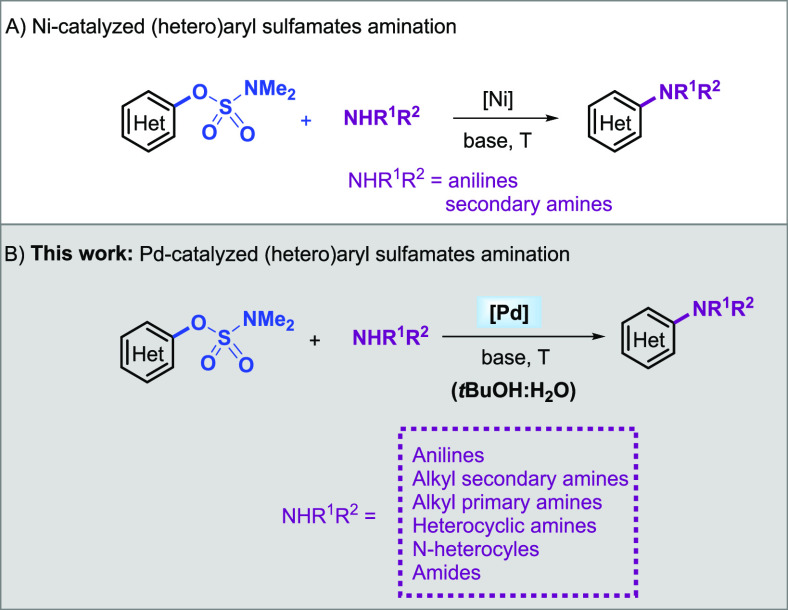
Scope of metal-catalyzed amination of aryl sulfamates.

Recently, we reported on the synthesis of a family of dialkylterphenyl phosphines and discussed their steric and electronic properties.^9^ These phosphines can adopt different coordination modes involving the P atom and one of the flanking aryl rings of the terphenyl fragment.^9a,10^ We showed that 2-aminobiphenyl palladacycles ligated with the bulkier ligand PCyp_2_Ar^Xyl2^ (Cyp = cyclopentyl; Ar^Xyl2^ = 2,6-bis(2,6-dimethylphenyl)phenyl) are excellent precatalysts for the Buchwald–Hartwig reaction of deactivated aryl chlorides with a variety of *N*-nucleophiles.^11^ To expand the array of electrophiles that can be applied in this transformation, we focused on challenging aryl sulfamates, since, to our knowledge, they have not been used as coupling partners in palladium-catalyzed Buchwald–Hartwig aminations.^4,12^ Furthermore, the substrate scope of Ni catalysts reported for the amination of aryl sulfamates remains limited to anilines and secondary alkyl amines (Figure 1A). Catalyst systems that accomplish the coupling of sulfamates with a wide range of substrates are necessary to further broadening the synthetic utility of this protocol.

Here, we report a versatile Pd/phosphine catalyst for the amination of aryl sulfamates with a broad scope of *N*-nucleophiles, including anilines, primary and secondary alkyl amines, heteroaryl amines, *N*-heterocycles, and primary amides (Figure 1B). In addition, we provide evidence by DFT calculation that in the polar protic medium, a cationic pathway is operating.

## Results and Discussion

In a previous work, we described the catalytic capability of a cationic *N*-methyl-2-aminobiphenyl palladacycle supported by sterically demanding phosphine PCyp_2_Ar^Xyl2^, **1**, in the amination of aryl chlorides.^11b^ The advantage of this precatalyst over the parent 2-aminobiphenyl palladacycle is that its activation in the presence of the base releases *N*-methyl carbazole, a byproduct that cannot hinder the catalytic reaction.^13^

Using precatalyst **1** (2.5 mol %), we examined the coupling of naphthalen-1-yl dimethylsulfamate with aniline applying the reaction conditions developed for the amination of aryl chlorides,^11b^ namely, NaO*t*Bu, as the base, dioxane as the solvent, and 110 °C as the reaction temperature. Under these conditions, a promising conversion to the expected *N*-phenylnaphthalen-1-amine, **4a**, of 27% was observed by GC analysis (Table 1, entry 1). To further improve the yield, different solvents were investigated (entries 2–5). Either toluene or DMF provided lower conversions. The use of a protic solvent, such as *t*BuOH, which has been successfully applied in Pd-catalyzed C-N couplings,^14^ also resulted in lower yield of the product. It has been found that the addition of water can enhance the rate of the amination.^14b,15^ To our delight, the use of a 8:1 (vol.) mixture of *t*BuOH:H_2_O significantly increased the conversion, but for driving the reaction to completion, a 1:1 mixture of *t*BuOH and water was crucial, product **4a** being isolated in 97% yield (entries 6–8; see also Table S1). Lowering the catalyst loading or using bases other than NaO*t*Bu reduced the reaction efficiency (entries 9–11); in the latter case, decomposition of the naphthyl sulfamate into naphthol was observed. Notably, complete conversion was also achieved at temperatures as low as 60 °C (entry 12), and even at room temperature, **1** proved to be reactive giving product **4a** in 74% yield (entry 13). However, a reaction temperature of 110 °C was used to study the reaction scope to accomplish the coupling of more challenging substrates.

**Table 1:**
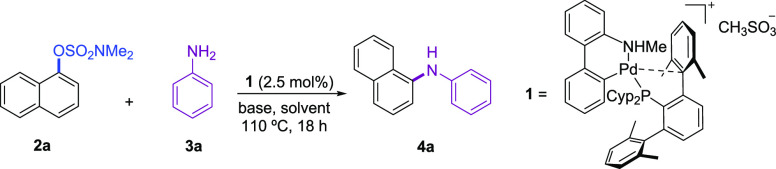
Screening of Conditions for the Coupling of Naphthalen-1-yl Dimethylsulfamate and Aniline^a^

entry	base	solvent	conversion^b^ (%)
1	NaO*t*Bu	dioxane	27
2	NaO*t*Bu	THF	12
3	NaO*t*Bu	toluene	9
4	NaO*t*Bu	DMF	15
5	NaO*t*Bu	*t*BuOH	4
6	NaO*t*Bu	*t*BuOH:H_2_O (8:1)	43
7	NaO*t*Bu	*t*BuOH:H_2_O (3:1)	94
8	NaO*t*Bu	*t*BuOH:H_2_O (1:1)	100 (97)^c^
9^d^	NaO*t*Bu	*t*BuOH:H_2_O (1:1)	90 (85)^c^
10	LiO*t*Bu	*t*BuOH:H_2_O (1:1)	99 (91)^c^
11	NaOH	*t*BuOH:H_2_O (1:1)	100 (90)^c^
12^e^	NaO*t*Bu	*t*BuOH:H_2_O (1:1)	100 (92)^c^
13^f^	NaO*t*Bu	*t*BuOH:H_2_O (1:1)	(74)^c^

a Reaction conditions: naphthyl sulfamate (1 mmol), amine (1.2 mmol), base (1.2 mmol), **1** (0.025 mmol), solvent (2 mL), *T* = 110 °C, 18 h (unoptimized).

b Conversion estimated by GC analysis of the reaction mixtures.

c Yields of isolated products (average of two runs).

d **1** (0.020 mmol).

e *T* = 60 °C.

f Reaction performed at room temperature.

Using the optimized reaction conditions, we tested other ligands using the corresponding ligated *N*-methyl-2-aminobiphenyl palladacycles (see Table S2). Only with XPhos-supported palladacycle was the conversion comparable to that obtained with our precatalyst. However, precatalyst **1** outperformed the catalytic abilities of the XPhos-supported precatalyst when we examined the amination of other substrate combinations (see Table S3).

**Scheme 1:**
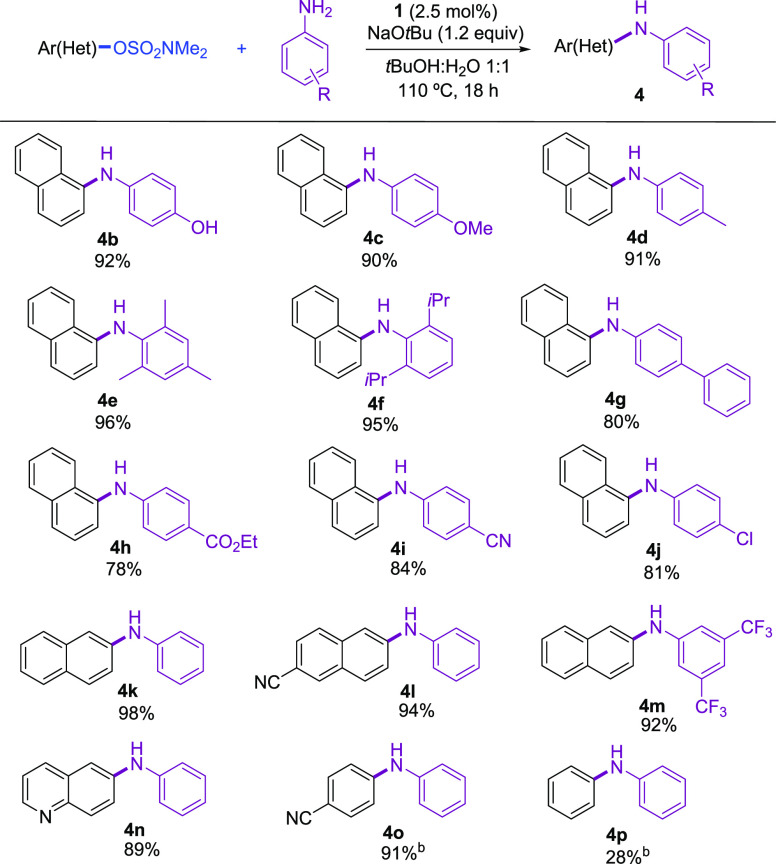
Pd-Catalyzed *N*-Arylation of Anilines with Aryl Sulfamates Reaction conditions: aryl sulfamate (1.0 mmol), amine (1.2 mmol), NaOtBu (1.2 mmol), **1** (0.025 mmol), solvent (2 mL), 110 °C, 18 h. Isolated yields of pure products. Reaction performed with 3 mol % catalyst loading.

Under the optimized conditions, we examined the coupling of a variety of aryl sulfamates and anilines. As shown in Scheme 1, sulfamates derived from 1- and 2-naphthol could be successfully employed as reactant partners in this transformation (**4b**–**4m**). Furthermore, the catalytic system tolerated a quinoline core in the electrophile (**4n**). The nonfused ring *p*-cyanophenyl sulfamate could be efficiently coupled with aniline (**4o**); however, poor results were obtained with the less reactive phenyl sulfamate (**4p**; see Table S4). Regarding the aniline, the scope was broad. Both, electron-rich and electron-poor anilines provided the corresponding coupling products in yields higher than 81% (**4b**–**4j**, **4m**). Moreover, *ortho-*substituted anilines were arylated in excellent yields (**4e**, **4f**). Interestingly, naphthyl sulfamates could be selectively coupled with anilines bearing chloride (**4j**) or free hydroxyl functionalities (**4b**), avoiding the need of protecting groups or the use of weaker bases in the latter case.

**Scheme 2:**
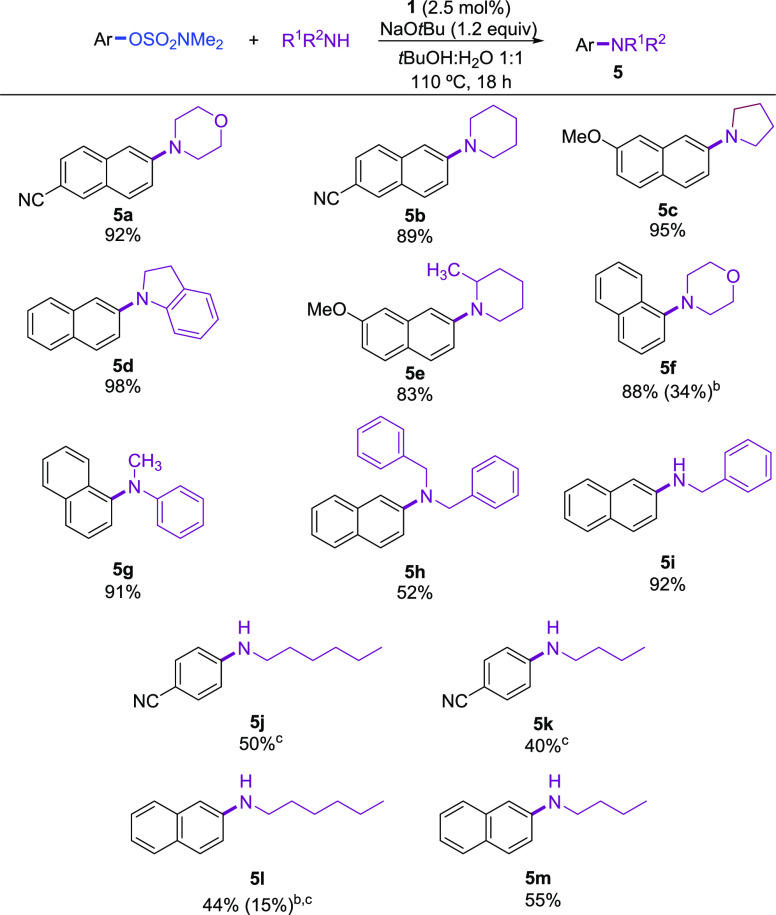
Pd-Catalyzed *N*-Arylation of Aliphatic Amines with Aryl Sulfamates Reaction conditions: aryl sulfamate (1.0 mmol), amine (1.2 mmol), NaO*t*Bu (1.2 mmol), **1** (0.025 mmol), solvent (2 mL), 110 °C, 18 h. Isolated yields of pure products. Conversion obtained using the Pd-XPhos precatalyst (Table S3). 4 mol % **1**.

Next, the scope of aliphatic amines was explored (Scheme 2). Both cyclic and acyclic secondary amines proved to be suitable substrates furnishing the arylated products in useful synthetic yields, using the optimized reaction conditions (Scheme 2, **5a**–**h**). Primary aliphatic amines have not been previously tested in Ni-catalyzed amination of aryl sulfamates. We found that our catalyst system enabled the coupling of benzylamine with naphthalen-2-yl sulfamate in high yield (**5i**). Moreover, linear alkyl primary amines could also be arylated with both naphthyl and phenyl-derived sulfamates, albeit in moderate yield (**5j**–**5m**).

**Scheme 3:**
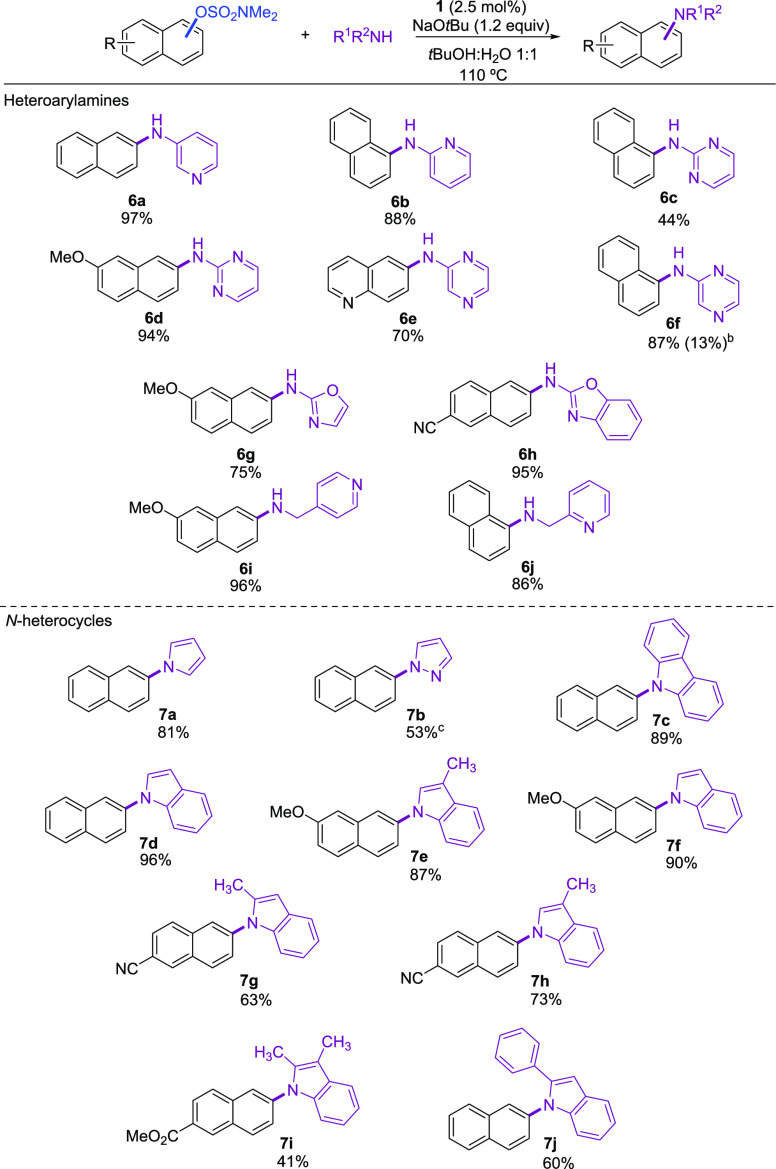
Pd-Catalyzed *N*-Arylation of Heteroarylamines and *N*-Heterocycles with Aryl Sulfamates Reaction conditions: aryl sulfamate (1.0 mmol), amine (1.2 mmol), NaO*t*Bu (1.2 mmol), **1** (0.025 mmol), solvent (2 mL), 110 °C, 18 h. Isolated yields of pure products. Conversion obtained using the Pd-XPhos precatalyst (Table S3). 5 mol % **1**.

In light of the reactivity displayed by precatalyst **1** toward amines, we focused on more challenging *N*-nucleophiles like heteroarylamines and *N*-heterocycles, since they are present as substructures in biologically active molecules, natural products, and pharmaceuticals.^12a,16^ An array of substrate combinations were screened under the optimized conditions (Scheme 3). Regarding the heteroarylamines, 2- and 3-aminopyridine, 2-aminopyrimidine, and 2-aminopyrazine were successfully arylated providing the corresponding diarylamines in isolated yields ranging from 44 to 97% (Scheme 3, **6a**–**f**). 2-Aminooxazol and 2-aminobenzoxazol were efficiently coupled with electron-rich and electron-deficient naphthyl sulfamate derivatives (**6g**, **6h**). Pyridine methanamines were found compatible with the catalyst system, affording the *N*-arylated heterocycles in high yields (**6i**, **6j**). Moreover, *N*-heterocycles such as pyrrole, pyrazole, and carbazole reacted with naphthalen-2-yl sulfamate, giving the desired cross-coupling products in moderate to high yields (Scheme 3, **7a**–**7c**). Chemoselective *N*-arylation of indoles was efficiently accomplished (**7d**–**f**, **7h**) although, with more hindered 2-substituted indoles, slightly lower yields were obtained (**7g**, **7i**, **7j**). To our knowledge, these results represent the first use of aryl sulfamates as electrophiles in the amination of a variety of *N*-nucleophiles that are relevant in pharmaceutical synthesis.

**Scheme 4:**
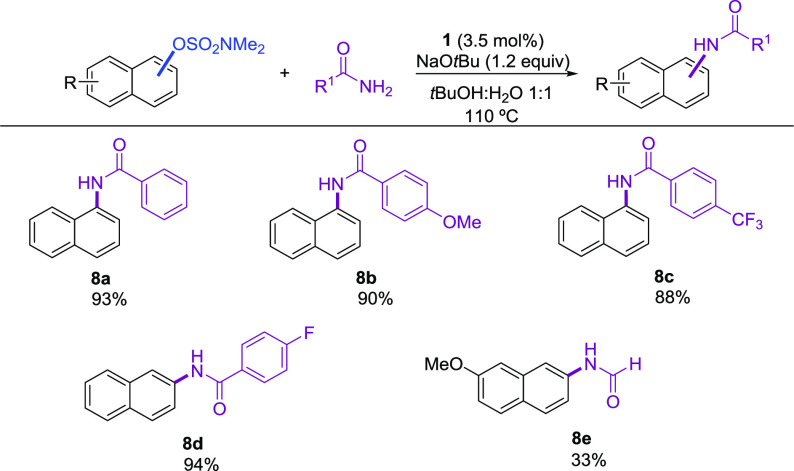
Pd-Catalyzed *N*-Arylation of Amides with Aryl Sulfamates^a^ Reaction conditions: aryl sulfamate (1.0 mmol), amine (1.2 mmol), NaOtBu (1.2 mmol), **1** (0.035), solvent (2 mL), 110 °C, 18 h. Isolated yields of pure products.

Amides are problematic substrates in Pd-catalyzed C–N cross-coupling reactions, due to their reduced nucleophilicity together with their tendency to form k^2^-amidate complexes, responsible for retarding the rate of the reductive elimination step.^17^ However, despite these shortcomings, Pd-based catalysts have been developed that enable the *N*-arylation of amides even with C–O electrophiles,^14b,18^ albeit limited to the more reactive aryl sulfonates. We were pleased to find that parent benzamide and electron-rich and electron-poor benzamide derivatives were all effective *N*-arylated using the disclosed protocol (Scheme 4, **8a**–**d**).

Formamide proved to be more difficult resulting in modest yield of the desired product (**8e**). We are aware of a single example in which an aryl sulfamate was used as electrophile in a Ni-catalyzed *N*-arylation of primary amides.^19^

The catalytic cycle for the aryl amination reactions is well established^20^ and involves the three main steps common to any cross-coupling catalytic manifold: the oxidative addition, the ligand exchange (amine coordination and deprotonation), and the reductive elimination. Almost all the computational studies on C–N coupling reactions have been carried out with aryl halides as model electrophiles. To our knowledge, there is only one theoretical report, focused on the role of DBU as a base in C–N couplings with Pd/phosphine catalysts, in which a phenol-derived electrophile, *p*-tolyl triflate, is considered in the calculations.^20h^ Bearing this in mind, we decided to explore the mechanism of the amination of aryl sulfamates with DFT calculations using (PCyp_2_Ar^Xyl2^)Pd(0) as the catalyst, naphthalen-1-yl dimethylsulfamate and aniline as the substrates, *t*BuO^–^ and OH^–^ as bases, and ethanol as the solvent, for the model chemical system. We used density functional methods (M06L/6-31g(d,p)/SDD//M06/6-311+g(2d,p)/LANL2TZ(f)).

**Figure 2:**
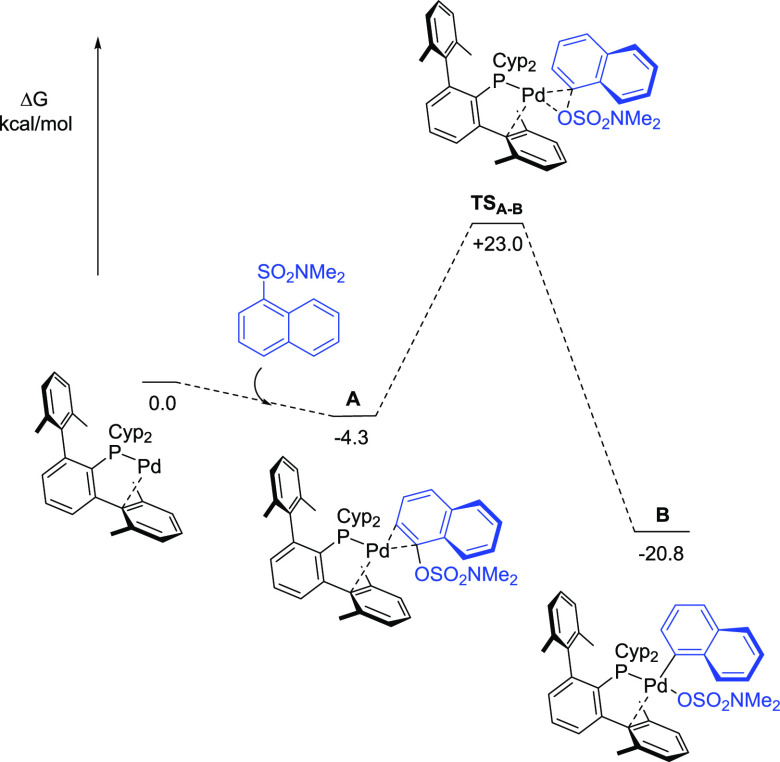
Gibbs free energy profile, in kcal mol^–**1**^, for the oxidative addition step.

We have shown that terphenyl phosphines can adopt pseudobidentate coordination modes, providing additional stabilization by noncovalent interactions between the metal center and a side ring of the terphenyl moiety.^9a,10^ Our calculations account for an extra stabilization of ca. −12 kcal mol^–1^ in ethanol for the PCyp_2_Ar^Xyl2^-ligated Pd(0) active species when the phosphine is binding in a pseudobidentate fashion (see Figure S1), in line with the results found for Nova et al.^8d^ Therefore, calculations have been carried out considering only the bidentate coordination mode of the phosphine ligand. Thus, initial binding of the naphthyl sulfamates to the active Pd(0) species generates η^2^-naphthyl sulfamate complex **A** located −4.3 kcal mol^–1^ below the separate reactants in the free energy surface (Figure 2), which retains the bidentate coordination mode of the phosphine (k^1^-P,η^2^-C_arene_). Subsequently, oxidative addition at **A** is exergonic by 16.5 kcal mol^–1^ and gives rise to species **B** through an energy barrier of 27.3 kcal mol^–1^. This barrier, which is only 1.8 kcal mol^–1^ higher than that obtained by Nova et al.,^8d^ in combination with the energy gain for the formation of **B**, renders this step not reversible. In accordance with the results obtained by Nova et al., in the oxidative addition product **B**, the sulfamate and the phosphine ligands display mutual *trans* orientation. Only one of the sulphonyl oxygen atoms of the sulfamate is bonded to the metal (Pd–O = 2.224 Å), and the phosphine retained the bidentate coordination (k^1^-P,η^2^-C_arene_), with the metrics for **B** being (Pd-C1′ = 2.511 Å, Pd-C2′ = 2.727 Å). To account for the lower reactivity observed for phenyl sulfamate derivatives, the oxidative addition of various *p*-substituted phenyl sulfamates (H, OH, CN) has been performed. Neutral or electron-donating substituents at the *p*-position of the aryl ring increase both the energy of the η^2^-sulfamate complex and the energy of the oxidative addition transition state, when compared with activated aryl sulfamates or naphthyl sulfamate (see Figure S2). This is consistent with our experimental observations.

**Figure 3:**
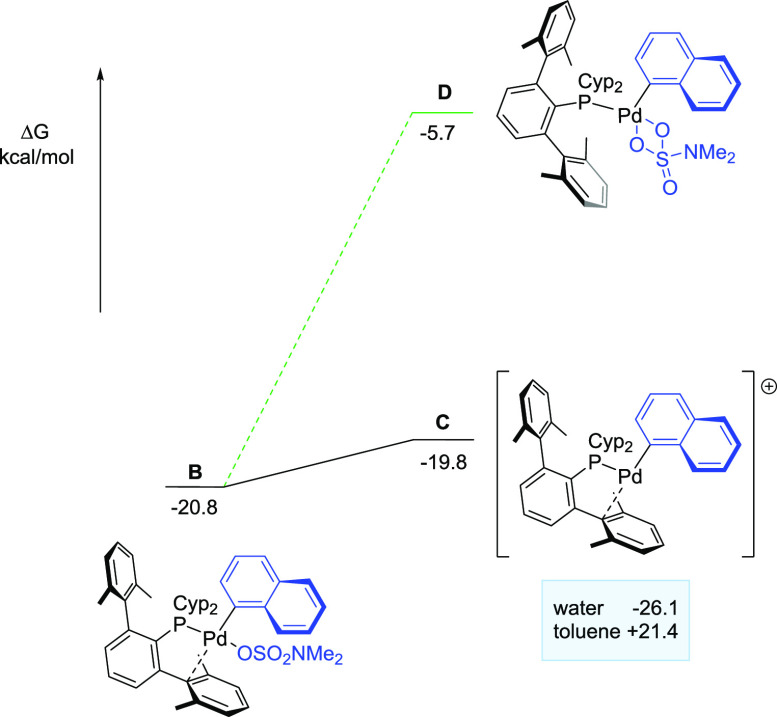
Energy profile of the evaluated pathways for ligand exchange stage. Gibbs free energy profiles are in kcal mol^–1^.

For the ligand exchange step, first the oxidative addition intermediate **B** dissociates the sulfamate anion to give a T-shaped cationic complex **C**, where the position *trans* to the phosphine is vacant. This charge separation event is facilitated by polar solvents.^21^ In fact, calculations carried out with the continuum SMD model yielded energies of −19.8 and −26.1 kcal mol^–1^ for separate **C** and sulfamate in ethanol and water, respectively. However, the same charge separation event calculated in toluene results in the separate fragments lying +21.4 kcal mol^–1^ above the origin (Figure 3). These results support that polar protic solvent mixtures (ethanol and water) favor a dissociative pathway involving the formation of cationic complex **C** with retention of the secondary interaction with the terphenyl moiety, in line with the experimental observations (see Table 1, entry 6).

Alternatively, the loss of the interaction between the metal and the flanking ring of the terphenyl substituent could also generate an open coordination site on the metal in intermediate **B**. However, the resulting species, **D**, which features k^2^-O coordination of the sulfamate is located 14.1 kcal mol^–1^ higher in energy than **C** (Figure 3). While **D** may be an accessible intermediate at the temperature at which reaction occurs, this pathway fails to explain the effect of the polar solvent mixture *t*BuOH:H_2_O in facilitating the reaction.

**Figure 4:**
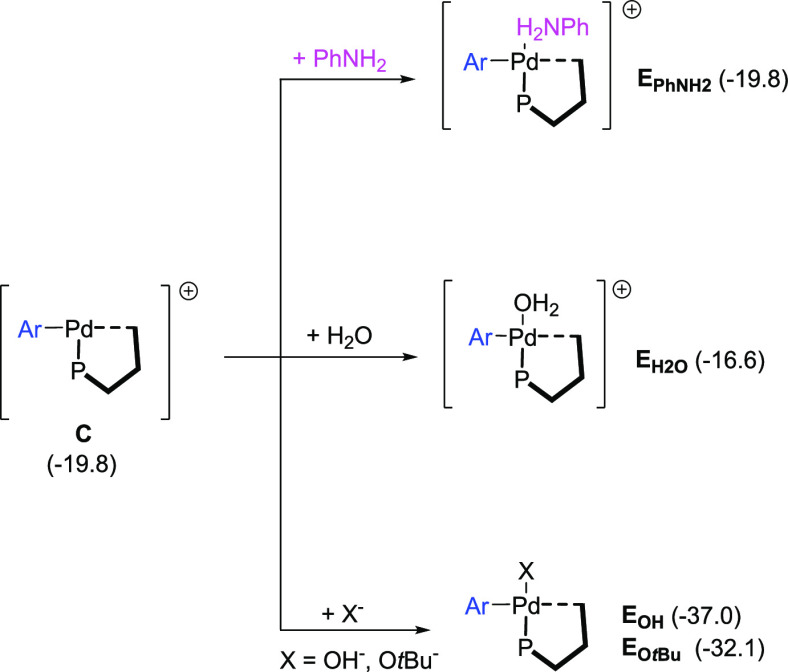
Reaction pathways evaluated for ligand coordination to **C**. In parenthesis: Gibbs energy in kcal mol^–**1**^.

While aniline can coordinate to the metal center in the cationic intermediate **C** to yield the corresponding amino complex, other ligands present in the reaction mixture may also form intermediates worth considering in the analysis of the reaction mechanism (Figure 4). Consequently, we explored the coordination of aniline, water, OH^–^, and *t*BuO^–^.^22^

The coordination of aniline to **C** is thermoneutral (Figure 4), rendering cationic intermediate **E_PhNH2_**. Moreover, the association of H_2_O to intermediate **C** (Figure 4) yields intermediate **E**_**H2O**_ with a relative Gibbs energy of −16.6 kcal mol^–1^, which is 3.2 kcal mol^–1^ higher than that of the aniline adduct **E**_**PhNH2**_. Therefore, H_2_O is not expected to be competitive with aniline for coordination to intermediate **C**. However, OH^–^ coordinates to **C** to form neutral species **E_OH_** located at −37.0 below the reactants (Figure 4). Similarly, *t*BuO^–^ forms a stable neutral adduct **E_O*t*Bu_** but this species is present in very low concentrations (see below) in solution. The participation of the latter in the catalysis is deemed of little importance, and its discussion is relegated to the SI, while the role of the former is shown in the following paragraphs.

**Figure 5:**
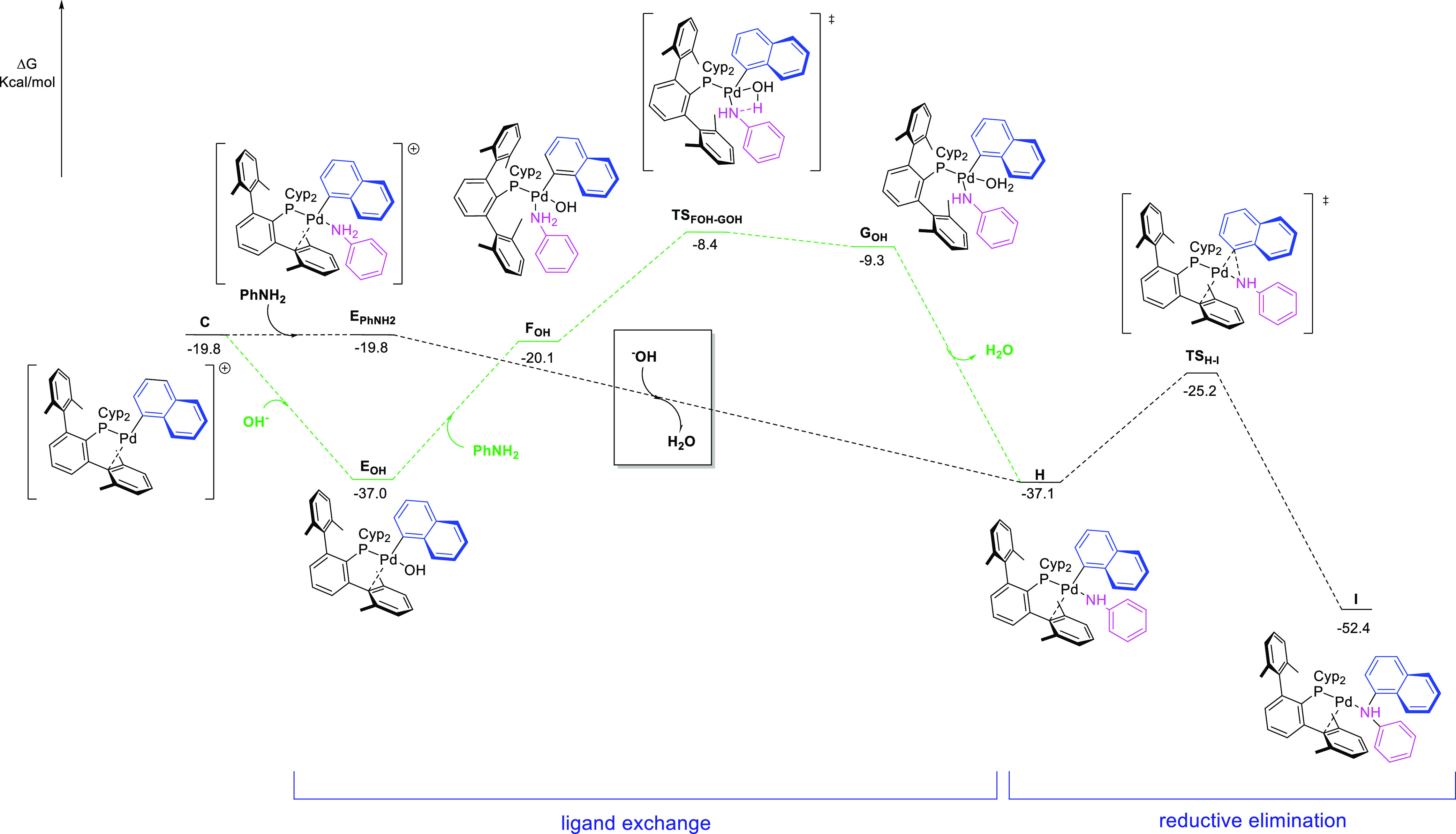
Energy profile of the proposed pathways for the ligand exchange step and reductive elimination. Gibbs energies are in kcal mol^–1^.

Following the above results, we envisioned two pathways for the ligand exchange step. The more favored one (shown in black in Figure 5) starts with cationic intermediate **E_PhNH2_**, which undergoes intermolecular deprotonation of the coordinated aniline by OH^–^. This step shows to be strongly favored thermodynamically (Δ*G* = −17.7 kcal mol^–1^), and importantly, the proton transfer occurs with a negligible energy barrier.^23^ The resulting species **H**, which displays the anilido ligand in the *trans* position to phosphorus, has a relative Gibbs energy of −37.1 kcal mol^–1^.

On the contrary, in the alternative pathway (shown in green in Figure 5), OH^–^ is acting as a ligand and as a base. From neutral intermediate **E_OH_**, coordination of aniline delivers intermediate **F_OH_**, located at −20.1 kcal mol^–1^. Intramolecular deprotonation of aniline is endergonic and takes place through **TS_FOH-GOH_**. The overall barrier from intermediate **E_OH_** is 28.6 kcal mol^–1^, slightly higher than that calculated for the oxidative step and higher than the reverse barrier for the dissociation of OH^–^ in **E_OH_** to regenerate **C**. It is worth noting that all attempts to detect the corresponding Pd-hydroxo species experimentally were unsuccessful.

These considerations were further supported by application of microkinetic modeling. These analyses can give information about the evolution of the concentration of each species with time considering rate constants provided by DFT calculations.^24^ In our case, the microkinetic model indicated that the calculated barrier for oxidative addition may be overestimated by ca. 2 kcal mol^–1^ at 383 K and, more importantly, it revealed that **E_H2O_**, **E_OH_**, and **E_O*t*Bu_** were present in very low concentration in the reaction mixture during the catalysis (see Figures S3 and S4 in the Supporting Information). In addition, the microkinetic model verified that the contribution of any pathway other than the one starting from intermediate **E_PhNH2_** to the formation of the C–N coupling product is negligible.

Finally, the last stage of the catalytic cycle is the formation of the C–N bond from intermediate **H**. The reductive elimination takes place through **TS_H-I_** (Figure 5) with an activation energy barrier of 11.9 kcal·mol^–1^, generating intermediate **I**, in which the C–N product is coordinated to the Pd(0) center through the N atom. To close the catalytic cycle, displacement of the diarylamine by naphthalen-1-yl dimethylsulfamate occurs with an energy cost of 2.25 kcal mol^–1^.

## Conclusions

In summary, we have developed a general Pd-based catalytic system for the amination of synthetically versatile aryl sulfamates. This protocol allows use of a broad range of *N*-nucleophiles including anilines, secondary amines, primary alkyl amines, heteroaryl amines, *N*-heterocycles, and primary amides. The use of a mixture of polar protic solvents (*t*BuOH:H_2_O) has been found to be crucial to attaining high conversions. Computational studies show that oxidative addition of aryl sulfamate is the rate-limiting step and that in polar protic solvents, the reaction proceeds through a cationic pathway.

## Supporting Information Available

General considerations; optimization experiments; unsuccessful results with aryl sulfamates; synthesis of aryl sulfamate substrates; general catalytic procedures; characterization data of reaction products; NMR spectra of compounds; computational details; microkinetic model (PDF)
Optimized coordinates for calculated structures (XYZ)

## Supplementary Material

cs3c03166_si_001.pdf

cs3c03166_si_002.xyz
