# Supplementary material for: Broad-Scope Amination of Aryl Sulfamates Catalyzed by a Palladium Phosphine Complex
Source: ACS Catal. 2023 Aug 4;13(16):10945–52. doi: 10.1021/acscatal.3c03166 (PMC10443792; doi:10.1021/acscatal.3c03166)
Supplement: Supplementary file 1 — cs3c03166_si_001.pdf [file cs3c03166_si_001.pdf]

Supporting Information

Broad Scope Amination of Aryl Sulfamates Catalyzed by a Palladium Phosphine Complex.

Andrea Monti,[†] Joaquín López-Serrano,[‡,*] Auxiliadora Prieto,[§,*] M. Carmen Nicasio[†,*]

[†]Departamento de Química Inorgánica, Universidad de Sevilla, Aptdo 1203, 41071 Sevilla, Spain.

[‡]Instituto de Investigaciones Químicas (IIQ), Departamento de Química Inorgánica and Centro de Innovación Química Avanzada (ORFEO-CINQA), Universidad de Sevilla and CSIC, 41092 Sevilla, Spain.

§Laboratorio de Catálisis Homogénea, Unidad Asociada al CSIC, CIQSO-Centro de Investigación en Química Sostenible and Departamento de Química, Campus de El Carmen s/n, Universidad de Huelva, 21007 Huelva, Spain.

joaquin.lopez@iiq.csic.es; maria.prieto@diq.uhu.es; mnicasio@us.es

Table of contents

1. General considerations	S2
2. Optimization experiments.	S3-S4
3. Table S4. Unsuccessful results with aryl sulfamates.	S5
4. Synthesis of aryl sulfamate substrates.	S5-S7
5. General catalytic procedures.	S7
6. Characterization data of reaction products.	S8-S27
7. NMR spectra of compounds.	S28-S72
8. Computational details	S73-S76
9. Microkinetic model	S77-S80
10. References	S80-S82

1. General considerations.

All preparations and manipulations were carried out under oxygen-free nitrogen, using conventional Schlenk techniques. Solvents were rigorously dried and degassed before use. $PCyp_2Ar^{Xyl2}$,[1] [Pd(N-methyl-2-aminobiphenyl)($PCyp_2Ar^{Xyl2}$)](OMs) **1**,[2a] [Pd(2-aminobiphenyl)($PCyp_2Ar^{Xyl2}$)](OMs) **Pd-L2**,[2b] [Pd(N-methyl-2-aminobiphenyl)(L)](OMs) (L = PMe_2Ar^{Xyl2}, **Pd-L3**;[2a] L = P*t*Bu_3, **Pd-P*t*Bu**;[2c] L = XPhos, **Pd-XPhos**[2d]), 1-naphthyl *N,N*-dimethylsulfamate (**2a**),[3] 6-cyano-2-naphthyl-*N,N*-dimethylsulfamate (**2c**),[3] 6-quinolinyl *N,N*-dimethylsulfamate (**2d**),[3] *N,N*-dimethylbenzenesulfonamide (**2f**),[3] 7-methyl-6-((*N*,*N*-dimethylsulfamoyl)oxy)-2-naphthoate (**2h**),[3] were synthesized by following previously reported procedures. Reagents were purchased from commercial suppliers and used without further purification. Solution NMR spectra were recorded on Bruker Avance III 300 MHz, Bruker Avance III 500 MHz, Bruker Avance NEO 300 MHz, Bruker Avance NEO 500 MHz. The ^{1}H and ^{13}C resonances of the solvent were used as the internal standard and the chemical shifts are reported relative to TMS. High resolution mass spectra were registered on Orbitrap Elite Mass Spectrometer at the Centro de Investigación Tecnología e Innovación, CITIUS (Universidad de Sevilla).

2. Optimization experiments.

Table S1. Effect of solvent and base on C-N coupling yield.[a]

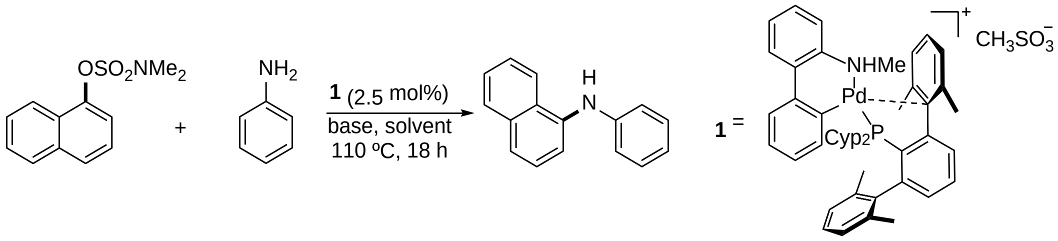

entry	base	solvent	conversion[b] (%)
1	NaO*t*Bu	Dioxane	27
2	NaO*t*Bu	THF	12
3	NaO*t*Bu	Toluene	9
4	NaO*t*Bu	DMF	15
5	NaO*t*Bu	*t*BuOH	4
6	NaO*t*Bu	*t*BuOH:H_2O (8:1)	43
7	NaO*t*Bu	*t*BuOH:H_2O (3:1)	94
8	NaO*t*Bu	*t*BuOH:H_2O (1:1)	100 (97)[c]
9[d]	NaO*t*Bu	*t*BuOH:H_2O (1:1)	90 (85)
10	NaO*t*Bu	H_2O	-
11	LiO*t*Bu	*t*BuOH:H_2O (1:1)	99 (91)[c]
12	NaOH	*t*BuOH:H_2O (1:1)	100 (90)[c]
13[e]	NaO*t*Bu	*t*BuOH:H_2O (1:1)	100 (92)[c]
14[f]	NaO*t*Bu	*t*BuOH:H_2O (1:1)	(74)[c]

[a] Reactions conditions: naphthyl sulfamate (1 mmol), amine (1.2 mmol), base (1.2 mmol), 1 (0.025 mmol), solvent (2 ml), T = 110 ºC, 18 h (unoptimized). [b] Conversion estimated by GC analysis of the reaction mixtures. [c] Yields of isolated products (average of two runs). [d] **1** (0.020 mmol). [e] T = 60 ºC. [f] Reaction performed at room temperature.

Table S2. Catalyst screen.

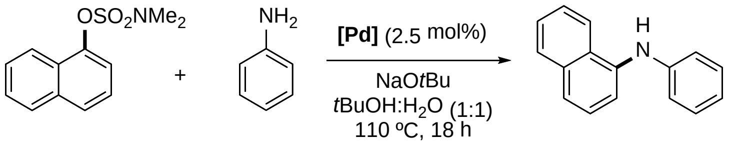

[Pd] =

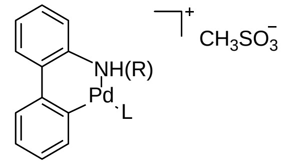

R = Me; L = PCyp$_2$Ar^{Xyl2}; **1**
R = H; L = PCyp$_2$Ar^{Xyl2}; **Pd-L2**
R = Me; L = PMe$_2$Ar^{Xyl2}; **Pd-L3**
R = Me; L = P*t*Bu$_3$; **Pd-P*t*Bu**
R = Me; L = XPhos; **Pd-XPhos**

Precatalyst	**1**	**Pd-L2**	**Pd-L3**	**Pd-P*t*Bu$_3$**	**Pd-XPhos**
Conversion[a] (%)	100 (97)[b]	92 (86)[b]	32	2	96 (90)[b]

[a] Conversion estimated by GC analysis of the reaction mixtures. [b]Yields of isolated products

Table S3. Comparison of catalytic abilities of 1 and Pd-XPhos.[a]

Complex **1** was compared against **Pd-XPhos** for C-N coupling using different substrate combinations.

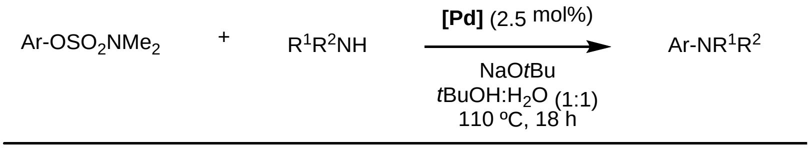

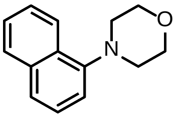

1: 100% (88%)[b]
Pd-XPhos: 34%

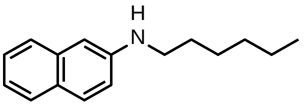

1: 50% (44%)[b,c]
Pd-XPhos: 15%[c]

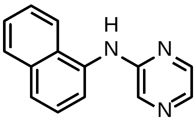

1: 100% (87%)[b]
Pd-XPhos: 13%

[a] Reaction conditions: aryl sulfamate (1.0 mmol), amine (1.2 mmol), NaOtBu (1.2 mmol), 1 (0.025 mmol), solvent (2 mL), 110 ºC, 18 h. Conversion by GC using dodecane as internal standard. [b]Isolated yields of pure products. [c] Reaction performed with 4 mol% catalyst loading.

3. Table S4. Unsuccessful results with aryl sulfamates.[a]

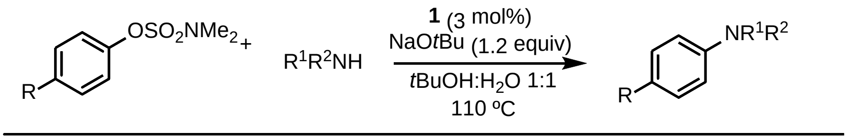

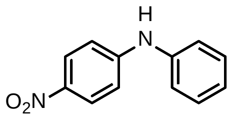

15%

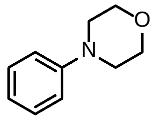

20%

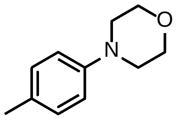

3%[b]

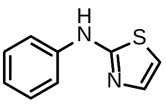

5%[b]

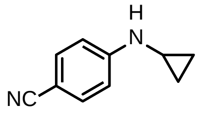

15%[c]

[a] Reaction conditions: aryl sulfamate (1.0 mmol), amine (1.2 mmol), NaOtBu (1.2 mmol), 1 (0.03 mmol), solvent (2 mL), 110 ºC, 18 h. Isolated yields of pure products. [b] Conversion by GC-cromatography. [c] Reaction performed with 4 mol% catalyst loading.

4. Synthesis of aryl sulfamate substrates.

4.1. Synthesis of 2-naphthyl *N,N*-dimethylsulfamate, 2b.

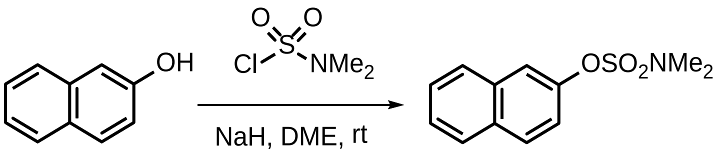

A round bottom flask was charged with NaH (0.60 g, 15.12 mmol, 1.2 equiv, 60% dispersion in oil). Then a solution of 2-naphthol (1.82 g, 12.60 mmol, 1 equiv) in DME (32 mL) was added dropwise via cannula to the NaH. A solution of dimethylsulfamoyl chloride (1.30 mL, 11.97 mmol, 0.95 equiv.) in DME (10 mL) was then added dropwise via cannula to the reaction vessel. The reaction was allowed to stir for 17 h, and then quenched with H_2O (2 mL). The volatiles were removed under reduced pressure, and then Et_2O (20 mL) and H_2O (15 mL) were added. The layers were separated, and the organic layer was washed successively with a solution of 1 M KOH (10 mL) and H_2O (20 mL). The combined aqueous layers were extracted with Et_2O (3 x 15 mL). The combined organic layers were then washed with brine (15 mL), dried over $MgSO_4$, and concentrated under reduced pressure to yield 2-naphthylsulfamate as a light brown solid (2.70 g, 89% yield).

^{1}H NMR (300 MHz, $CDCl_3$): δ 7.91-7.80 (m, 3H), 7.76 (d, 1H, *J* = 2.4 Hz), 7.57-7.45 (m, 2H), 7.42 (dd, 1H, *J* = 8.9; 2.4 Hz), 3.01 (s, 6H).

^{13}C NMR (75 MHz, $CDCl_3$): δ 148.0, 133.8, 131.9, 130.1, 128.0, 127.9, 127.0, 126.3, 120.9, 119.1, 38.9.

ESI-MS (Orbitrap): *m/z* calculated for $C_{12}H_{13}NO_3SNa^+$ $[M + Na]^+$ 274.0508, found 274.0509.

4.2. Synthesis of 6-cyano-2-naphthyl-*N,N*-dimethylsulfamate 2c.

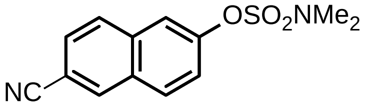

Following the procedure described for **2b**, this product is obtained as a yellow solid (940 mg, 38% yield).

^1H NMR (300 MHz, $CDCl_3$): δ 8.22 (s, 1H), 7.91 (t, 2H, *J* = 8.1 Hz), 7.80 (d, 1H, *J* = 1.6 Hz), 7.63 (d, 1H, *J* = 8.6 Hz), 7.52 (dd, 1H, *J* = 8.6; 1.6 Hz), 3.04 (s, 6H).

^{13}C NMR (75 MHz, $CDCl_3$): δ 150.2, 135.3, 133.9, 130.7, 130.5, 129.2, 127.5, 122.8, 119.0, 118.9, 109.8, 38.9.

ESI-MS (Orbitrap): *m/z* calculated for $C_{13}H_{12}N_2O_3SNa^+$ $[M + Na]^+$ 299.0461, found 299.0461.

4.3. Synthesis of 6-quinolinyl *N,N*-dimethylsulfamate (2d).

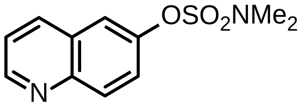

A round bottom flask was charged with NaH (0.43 g, 10.76 mmol, 1.2 equiv, 60% dispersion in oil), and cooled to 0 ºC. Then a solution of 6-hydroxyquinoline (1.29 g, 8.90 mmol, 1 equiv) in DME (24 mL) was added dropwise via cannula to the NaH. The resulting solution was warmed to rt for 10 min, and then cooled to 0 ºC. A solution of dimethyl sulfamoyl chloride (1 mL, 9.21 mmol, 1.03 equiv) in DME (6 mL) was then added dropwise via cannula to the reaction vessel. The reaction was warmed to rt, allowed to stir for 18 h, and then quenched with several drops of water. The solvent was removed under reduced pressure, and the solid material was dissolved in Et_2O (50 mL) and H_20 (15 mL), and transferred to a separatory funnel. The layers were separated, and the organic layer was washed with 1 M KOH (15 mL), then H_2O (15 mL). The combined aqueous layers were extracted with Et_2O (3x20 mL). The combined organic layers were then washed with brine (15 mL), dried over $MgSO_4$, and concentrated under reduced pressure to yield 6-quinolinyl sulfamate as a brown solid (1.40 g, 56% yield).

Spectroscopic data were consistent with those reported in the literature.[3]

4.4. Synthesis of 7-methoxy-2-naphthyl *N,N*-dimethylsulfamate (2g).

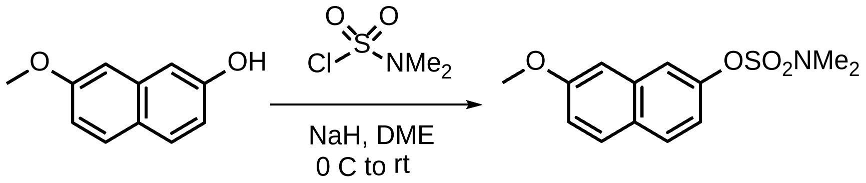

A round bottom flask was charged with NaH (0.43 g, 10.76 mmol, 1.2 equiv, 60% dispersion in oil), and cooled to 0 ºC. Then a solution of 7-methoxy-2-naphthol (1.55 g, 8.90 mmol, 1 equiv) in DME (24 mL) was added dropwise via cannula to the NaH. The resulting solution was warmed to rt for 10 min, and then cooled to 0 ºC. A solution of dimethyl sulfamoyl chloride (1 mL, 9.21 mmol, 1.03 equiv.) in DME (6 mL) was then added dropwise via cannula to the reaction vessel. The reaction was warmed to rt, allowed to stir for 15 h, and then quenched with several drops of water. The solvent was removed under reduced pressure, and the solid material was dissolved in Et_2O (50 mL) and H_2O (15 mL), and transferred to a separatory funnel. The layers were separated, and the organic layer was washed with 1 M KOH (15 mL), then H_2O (15 mL). The combined aqueous layers were extracted with Et_2O (3x20 mL). The combined organic layers were then washed with brine (15 mL), dried over $MgSO_4$, and concentrated under reduced pressure to yield 7-methoxy-2-naphthyl sulfamate as a white solid (1.93 g, 77% yield).

^{1}H NMR (300 MHz, $CDCl_3$): δ 7.76 (dd, 2H, *J* = 13.1; 8.9 Hz), 7.66 (d, 1H, *J* = 2.4 Hz), 7.25 (dd, 1H, *J* = 8.9; 2.4 Hz), 7.18-7.10 (m, 2H), 3.92 (s, 3H), 3.01 (s, 6H).

^{13}C NMR (75 MHz, $CDCl_3$): δ 158.6, 148.6, 135.3, 129.7, 129.4, 127.4, 119.3, 118.4, 118.1, 105.9, 55.5, 39.0.

ESI-MS (Orbitrap): *m/z* calculated for $C_{13}H_{15}NO_4SNa^+$ $[M + Na]^+$ 304.0614, found 304.0615.

5. General catalytic procedure for the C-N coupling.

The precatalyst **1** (2.5 mol%) and the base NaO*t*Bu (1.2 mmol) were placed into a vial equipped with a J Young tap containing a magnetic bar. The aryl sulfamate (1.0 mmol), the nitrogen *N*-nucleophile (1.2 mmol) and the mixture of solvents (2 mL) were added in turn, under a nitrogen atmosphere. The reaction mixture was stirred at 110 ºC for 18 h in an oil bath. The reaction mixture was allowed cooling to room temperature, diluted with ethyl acetate (10 mL) and filtered through a Celite plug. The solution was evaporated to dryness and the residue was purified by flash chromatography.

Some of the products have already been described in the literature. However, ^{1}H NMR spectra of these compounds used to identify them have been included herein along

with the references where their spectroscopic characterization appeared. For new products, all relevant spectroscopic and analytical data are provided.

6. Characterization data of reaction products.

***N*-phenylnaphthalen-1-amine[4] (Table 1, 4a)**

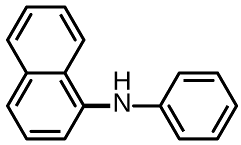

Following the general procedure, a mixture of **2a** (251.3 mg, 1 mmol), aniline (110 μL, 1.2 mmol), NaO*t*Bu (115.3 mg, 1.2 mmol), **1** (2.5 mol%) in *t*BuOH/H_2O 1:1 (2 mL) was stirred at 110 ºC for 18 h. The crude product was purified by column chromatography in silica gel, eluting with a mixture 1:20 ethyl acetate/hexanes to provide the title compound as a light brown solid. Yield: 212.4 mg, 97%.

^{1}H NMR (300 MHz, $CDCl_3$): δ 8.00 (d, 1H, *J* = 8.5 Hz), 7.85 (d, 1H, *J* = 8.8 Hz), 7.55 (dd, 1H, *J* = 7.2; 2.3 Hz), 7.51-7.41 (m, 2H), 7.40-7.32 (m, 2H), 7.24 (t, 2H, *J* = 7.7 Hz), 6.97 (d, 2H, *J* = 8.1 Hz), 6.90 (t, 1H, *J* = 7.2 Hz), 5.75 (br s, 1H).

4-(naphthalen-1-ylamino)phenol (Scheme 2, 4b)

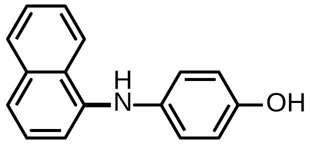

Following the general procedure, a mixture of **2a** (251.3 mg, 1 mmol), 4-hydroxyaniline (131.1 mg, 1.2 mmol), NaO*t*Bu (115.3 mg, 1.2 mmol), **1** (2.5 mol%) in *t*BuOH/H_2O 1:1 (2 mL) was stirred at 110 ºC for 18 h. The crude product was purified by column chromatography in silica gel, eluting with a mixture 1:5 ethyl acetate/hexanes to provide the title compound as a violet oil. Yield: 216.3 mg, 92%.

^{1}H NMR (300 MHz, $CDCl_3$): δ 8.11-7.80 (m, 2H), 7.61-7.41 (m, 3H), 7.40-7.24 (m, 1H), 7.10 (d, 1H, *J* = 7.5 Hz), 7.00 (d, 2H, *J* = 8.8 Hz), 6.81 (d, 2H, *J* = 8.8 Hz), 5.72 (br s, 1H).

^{13}C NMR (75 MHz, $CDCl_3$): δ 151.1, 141.1, 137.1, 134.8, 128.7, 126.3, 126.2, 125.5, 122.2, 121.5, 121.2, 121.1, 120.5, 119.5, 116.4, 111.9.

ESI-MS (Orbitrap): *m/z* calculated for $C_{16}H_{14}ON^+$ $[M + H]^+$ 236.1070, found 236.1070.

***N*-(4-Methoxyphenyl)naphthalen-1-amine[5] (Scheme 2, 4c)**

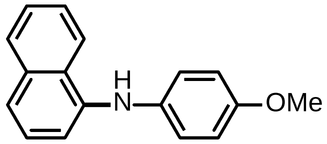

Following the general procedure, a mixture of **2a** (251.3 mg, 1 mmol), 4-methoxyaniline (147.7 mg, 1.2 mmol), NaO*t*Bu (115.3 mg, 1.2 mmol), **1** (2.5 mol%) in *t*BuOH/H_2O 1:1 (2 mL) was stirred at 110 ºC for 18 h. The crude product was purified by column chromatography in silica gel, eluting with a mixture 1:20 ethyl acetate/hexanes to provide the title compound as a light brown solid. Yield: 224.3 mg, 90%.

^{1}H NMR (300 MHz, $CDCl_3$): δ 8.02 (d, 1H, *J* = 7.6 Hz), 7.89 (d, 1H, *J* = 7.6 Hz), 7.57-7.45 (m, 3H), 7.37 (t, 1H, *J* = 7.8 Hz), 7.15 (d, 1H, *J* = 7.4 Hz), 7.09 (d, 2H, *J* = 8.8 Hz), 6.92 (d, 2H, *J* = 8.8 Hz), 5.89 (br s, 1H), 3.84 (s, 3H).

N-(4-methylphenyl)naphthalen-1-amine[6] (Scheme 2, 4d)

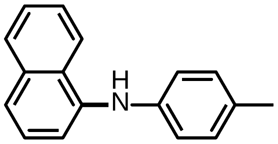

Following the general procedure, a mixture of **2a** (251.3 mg, 1 mmol), 4-methylaniline (128.6 mg, 1.2 mmol), NaO*t*Bu (115.3 mg, 1.2 mmol), **1** (2.5 mol%) in *t*BuOH/H_2O 1:1 (2 mL) was stirred at 110 ºC for 18 h. The crude product was purified by column chromatography in silica gel, eluting with a mixture 1:20 ethyl acetate/hexanes to provide the title compound as a brown solid. Yield: 213 mg, 91%.

^{1}H NMR (300 MHz, $CDCl_3$): δ 8.04 (d, 1H, J_{HH} = 7.5 Hz), 7.89 (d, 1H, J_{HH} = 7.5 Hz), 7.59-7.45 (m, 3H), 7.40 (t, 1H, *J* = 7.8 Hz), 7.31 (d, 1H, *J* = 7.5 Hz), 7.13 (d, 2H, *J* = 8.3 Hz), 6.98 (d, 2H, *J* = 8.3 Hz), 5.90 (br s, 1H), 2.35 (s, 3H).

N-(2,4,6-Trimethylphenyl)naphthalen-1-amine[7] (Scheme 2, 4e)

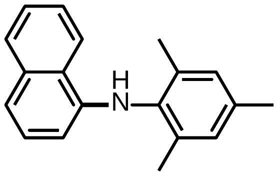

Following the general procedure, a mixture of **2a** (251.3 mg, 1 mmol), 1,3,5-trimethylaniline (168.6 µL, 1.2 mmol), NaO*t*Bu (115.3 mg, 1.2 mmol), **1** (2.5 mol%) in *t*BuOH/H_2O 1:1 (2 mL) was stirred at 110 ºC for 18 h. The crude product was purified by column chromatography in silica gel, eluting with hexanes to provide the title compound as an orange oil. Yield: 249.5 mg, 96%.

^{1}H NMR (300 MHz, $CDCl_3$): δ 8.12-8.09 (m, 1H), 7.90-7.87 (m, 1H), 7.59-7.51 (m, 2H), 7.34 (d, 1H, *J* = 8.1 Hz), 7.25 (t, 1H, *J* = 7.8 Hz), 7.04 (s, 2H), 6.26 (d, 1H, *J* = 7.4 Hz), 5.69 (br s, 1H), 2.39 (s, 3H), 2.22 (s, 6H).

N-[2,6-diisopropylphenyl)naphthalen-1-amine[8] (Scheme 2, 4f)

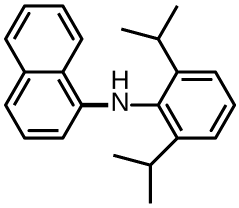

Following the general procedure, a mixture of **2a** (251.3 mg, 1 mmol), 2,6-diisopropylaniline (188.6 µL, 1.2 mmol), NaO*t*Bu (115.3 mg, 1.2 mmol), **1** (2.5 mol%) in *t*BuOH/H_2O 1:1 (2 mL) was stirred at 110 ºC for 18 h. The crude product was purified by column chromatography in silica gel, eluting with hexanes to provide the title compound as an orange oil. Yield: 286.5 mg, 95%.

^{1}H NMR (300 MHz, $CDCl_3$): δ 8.26-8.18 (m, 1H), 8.04-7.96 (m, 1H), 7.70-7.61 (m, 2H), 7.55-7.40 (m, 4H), 7.35 (t, 1H, *J* = 7.6 Hz), 6.38 (dd, 1H, *J* = 7.4; 1.0 Hz), 5.90 (br s, 1H), 3.34 (sept, 2H, *J* = 13.7; 6.9 Hz), 1.36 (d, 6H, *J* = 6.9 Hz), 1.27 (d, 6H, *J* = 6.9 Hz).

N-([1,1′-biphenyl]-2-yl)naphthalen-1-amine[9] (Scheme 2, 4g)

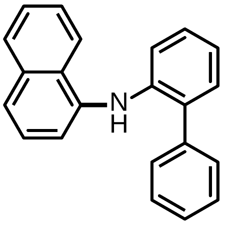

Following the general procedure, a mixture of **2a** (251.3 mg, 1 mmol), 2-aminobiphenyl (203.1 mg, 1.2 mmol), NaO*t*Bu (115.3 mg, 1.2 mmol), **1** (2.5 mol%) in *t*BuOH/H_2O 1:1 (2 mL) was stirred at 110 ºC for 18 h. The crude product was purified by column chromatography in silica gel, eluting with hexanes to provide the title compound as a light pink solid. Yield: 236.5 mg, 80%.

^{1}H NMR (300 MHz, $CDCl_3$): δ 7.93 (t, 2H, J = 7.7 Hz), 7.71-7.59 (m, 3H), 7.58-7.42 (m, 7H), 7.39 (d, 1H, J = 8.1 Hz), 7.32-7.22 (m, 1H), 7.16 (d, 1H, J = 8.1 Hz), 7.06 (t, 1H, J = 7.6 Hz), 6.06 (br s, 1H).

Ethyl 4-(naphthalen-1-ylamino)benzoate[10] (Scheme 2, 4h)

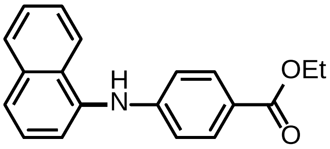

Following the general procedure, a mixture of **2a** (251.3 mg, 1 mmol), 4-(ethoxycarbonyl)aniline (198.2 mg, 1.2 mmol), NaO*t*Bu (115.3 mg, 1.2 mmol), **1** (2.5 mol%) in *t*BuOH/H_2O 1:1 (2 mL) was stirred at 110 ºC for 18 h. The crude product was purified by column chromatography in silica gel, eluting with a mixture 1:7 ethyl acetate/hexanes to provide the title compound as a light brown solid. Yield: 226.7 mg, 78%.

^{1}H NMR (300 MHz, $CDCl_3$): δ 7.98 (d, 1H, J = 8.1 Hz), 7.95-7.87 (m, 3H), 7.75-7.68 (m, 1H), 7.56-7.49 (m, 1H), 7.48-7.43 (m, 2H), 6.84 (d, 2H, J = 8.8 Hz), 6.28 (br s, 1H), 4.34 (q, 2H, J = 7.3 Hz), 1.37 (t, 3H, J = 7.1 Hz).

4-(Naphthalen-1ylamino)benzonitrile[11] (Scheme 2, 4i)

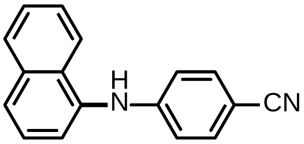

Following the general procedure, a mixture of **2a** (251.3 mg, 1 mmol), 4-cyanoaniline (141.8 mg, 1.2 mmol), NaO*t*Bu (115.3 mg, 1.2 mmol), **1** (2.5 mol%) in *t*BuOH/H_2O 1:1 (2 mL) was stirred at 110 ºC for 18 h. The crude product was purified by column chromatography in silica gel, eluting with a mixture 1:7 ethyl acetate/hexanes to provide the title compound as a brown solid. Yield: 205.9 mg, 84%.

^{1}H NMR (300 MHz, $CDCl_3$): δ 7.93 (t, 2H, J = 7.7 Hz), 7.77 (d, 1H, J = 7.7 Hz), 7.60-7.35 (m, 6H), 6.79 (d, 2H, J = 8.8 Hz), 6.28 (br s, 1H).

^{13}C NMR (75 MHz, $CDCl_3$): δ 150.1, 135.8, 134.9, 133.9, 129.6, 128.8, 126.7, 126.6, 126.4, 126.0, 122.4, 121.6, 120.2, 114.7, 101.0.

ESI-MS (Orbitrap): m/z calculated for $C_{17}H_{12}N_2Na^+$ $[M + Na]^+$ 267.0893, found 267.0894.

N-(4-chlorophenyl)naphthalen-1-amine[9] (Scheme 2, 4j)

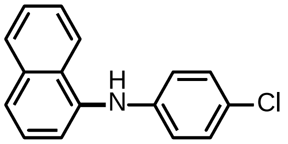

Following the general procedure, a mixture of **2a** (251.3 mg, 1 mmol), 4-chloroaniline (153.1 mg, 1.2 mmol), NaO*t*Bu (115.3 mg, 1.2 mmol), **1** (2.5 mol%) in *t*BuOH/H_2O 1:1 (2 mL) was stirred at 110 ºC for 18 h. The crude product was purified by column chromatography in silica gel, eluting with a mixture 1:20 ethyl acetate/hexanes to provide the title compound as a light brown solid. Yield: 204.8 mg, 81%.

^{1}H NMR (300 MHz, $CDCl_3$): δ 8.06-7.96 (m, 1H), 7.90 (dd, 1H, *J* = 7.3; 2.2 Hz), 7.64 (d, 1H, *J* = 8.1 Hz), 7.59-7.46 (m, 2H), 7.43 (t, 1H, *J* = 9.8 Hz), 7.34 (dd, 1H, *J* = 7.4; 1.2 Hz), 7.25-7.18 (m, 2H), 6.94-6.83 (m, 2H), 5.88 (br s, 1H).

N-phenylnaphthale-2-amine[12] (Scheme 2, 4k)

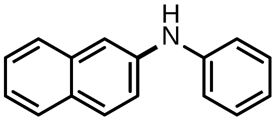

Following the general procedure, a mixture of **2b** (251.3 mg, 1 mmol), aniline (110 μL, 1.2 mmol), NaO*t*Bu (115.3 mg, 1.2 mmol), **1** (2.5 mol%) in *t*BuOH/H_2O 1:1 (2 mL) was stirred at 110 ºC for 18 h. The crude product was purified by column chromatography in silica gel, eluting with a mixture 1:20 ethyl acetate/hexanes to provide the title compound as a light brown solid. Yield: 213.5 mg, 98%.

^{1}H NMR (300 MHz, $CDCl_3$): δ 7.78 (d, 2H, *J* = 8.7 Hz), 7.68 (d, 1H, *J* = 8.2 Hz), 7.51-7.40 (m, 2H), 7.35 (t, 3H, *J* = 7.1 Hz), 7.25 (dd, 1H, *J* = 8,7; 2.4 Hz), 7.20 (d, 2H, *J* = 7.7 Hz), 7.03 (t, 1H, *J* = 7.3 Hz), 5.85 (br s, 1H).

6-(phenylamino)-2-naphthonitrile (Scheme 2, 4l)

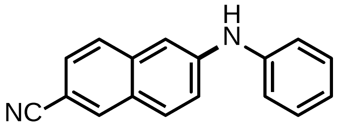

Following the general procedure, a mixture of **2c** (276.3 mg, 1 mmol), aniline (110 μL, 1.2 mmol), NaO*t*Bu (115.3 mg, 1.2 mmol), **1** (2.5 mol%) in *t*BuOH/H_2O 1:1 (2 mL) was stirred at 110 ºC for 18 h. The crude product was purified by column chromatography in silica gel, eluting with a mixture 1:5 ethyl acetate/hexanes to provide the title compound as a brown solid. Yield: 229.3 mg, 94%.

^{1}H NMR (300 MHz, $CDCl_3$): δ 8.04 (s, 1H), 7.74 (d, 1H, *J* = 8.9 Hz), 7.62 (d, 1H, *J* = 8.6 Hz), 7.48 (dd, 1H, *J* = 8.5; 1.5 Hz), 7.41-7.31 (m, 3H), 7.29-7.18 (m, 3H), 7.10 (td, 1H, *J* = 7.3; 1.2 Hz), 6.10 (br s, 1H).

^{13}C NMR (75 MHz, $CDCl_3$): δ 144.5, 141.2, 136.6, 133.9, 130.0, 129.7, 127.3, 127.2, 123.3, 120.6, 120.4, 120.1, 108.7, 105.7.

ESI-MS (Orbitrap): *m/z* calculated for $C_{17}H_{12}N_2Na^+$ $[M + Na]^+$ 267.0893, found 267.0894.

_N_-[3,5-Bis(trifluoromethyl)phenyl]naphthalen-2-amine (Scheme 2, 4m)

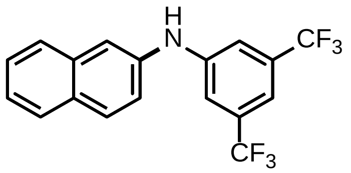

Following the general procedure, a mixture of **2b** (251.3 mg, 1 mmol), 3,5-bis(trifluoromethyl)aniline (187.4 µL, 1.2 mmol), NaO*t*Bu (115.3 mg, 1.2 mmol), **1** (2.5 mol%) in *t*BuOH/H_2O 1:1 (2 mL) was stirred at 110 ºC for 18 h. The crude product was purified by column chromatography in silica gel, eluting with hexanes to provide the title compound as a white solid. Yield: 326.2 mg, 92%.

^{1}H NMR (300 MHz, $CDCl_3$): δ 7.91-7.83 (m, 2H), 7.78 (d, 1H, J = 8.0 Hz), 7.58-7.45 (m, 6H), 7.28 (dd, 1H, J = 8.8; 2.3 Hz), 6.05 (br s, 1H).

^{13}C NMR (75 MHz, $CDCl_3$): δ 145.4, 138.1, 134.5, 133.0 (q, J_{CF} = 33 Hz), 130.6, 130.0, 128.0, 127.1, 127.0, 125.1, 123.5 (q, J_{CF} = 273 Hz), 121.1, 116.2, 115.8-115.5 (m), 113.4 (q, J_{CF} = 4 Hz).

ESI-MS (Orbitrap): *m/z* calculated for $C_{18}H_{12}NF_6^+$ $[M + H]^+$ 356.0868, found 356.0865.

_N_-Phenylquinolin-6-amine[13] (Scheme 2, 4n)

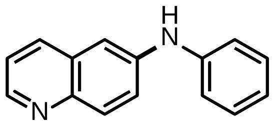

Following the general procedure, a mixture of **2d** (252.3 mg, 1 mmol), aniline (110 µL, 1.2 mmol), NaO*t*Bu (115.3 mg, 1.2 mmol), **1** (2.5 mol%) in *t*BuOH/H_2O 1:1 (2 mL) was stirred at 110 ºC for 18 h. The crude product was purified by column chromatography in silica gel, eluting with ethyl acetate to provide the title compound as a yellow solid. Yield: 194.9 mg, 89%.

^{1}H NMR (300 MHz, $CDCl_3$): δ 8.71 (dd, 1H, J = 4.2; 1.5 Hz), 7.99 (d, 1H, J = 9.0 Hz), 7.94 (d, 1H, J = 8.3 Hz), 7.42 (dd, 1H, J = 9.0; 2.6 Hz), 7.39-7.27 (m, 4H), 7.24-7.16 (m, 2H), 7.03 (td, 1H, J = 8.5; 1.1 Hz), 5.99 (br s, 1H).

4-(phenylamino)benzonitrile[14] (Scheme 2, 4o)

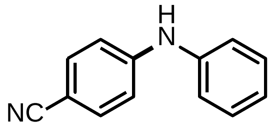

Following the general procedure, a mixture of **2e** (227.2 mg, 1 mmol), aniline (110 µL, 1.2 mmol), NaO*t*Bu (115.3 mg, 1.2 mmol), **1** (3 mol%) in *t*BuOH/H_2O 1:1 (2 mL) was stirred at 120 ºC for 18 h. The crude product was purified by column chromatography in silica gel, eluting with a mixture 1:8 ethyl acetate/hexanes to provide the title compound as a yellow solid. Yield: 175.8 mg, 91%.

^{1}H NMR (300 MHz, $CDCl_3$): δ 7.46 (d, 2H, J = 8.7 Hz), 7.40-7.31 (m, 2H), 7.22-7.15 (m, 2H), 7.14-7.08 (m, 1H), 6.98 (d, 2H, J = 8.8 Hz), 6.24 (br s, 1H).

Diphenylamine[15] (Scheme 2, 4p)

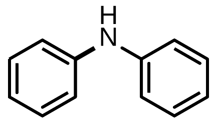

Following the general procedure, a mixture of **2f** (201.0 mg, 1 mmol), aniline (110 μL, 1.2 mmol), NaO*t*Bu (115.3 mg, 1.2 mmol), **1** (3 mol%) in *t*BuOH/H_2O 1:1 (2 mL) was stirred at 120 ºC for 18 h. The crude product was purified by column chromatography in silica gel, eluting with a mixture 1:20 ethyl acetate/hexanes to provide the title compound as a white solid. Yield: 47.8 mg, 28%.

^{1}H NMR (300 MHz, $CDCl_3$): δ 7.28 (td, 4H, *J* = 8.3; 1.9 Hz), 7.08 (dd, 4H, *J* = 8.5; 0.9 Hz), 6.94 (t, 2H, *J* = 7.3 Hz), 5.69 (br s, 1H).

6-morpholino-2-naphthonitrile (Scheme 3, 5a)

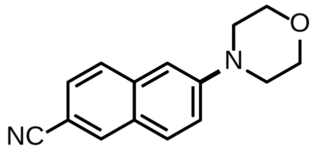

Following the general procedure, a mixture of **2c** (276.3 mg, 1 mmol), morpholine (104.6 μL, 1.2 mmol), NaO*t*Bu (115.3 mg, 1.2 mmol), **1** (2.5 mol%) in *t*BuOH/H_2O 1:1 (2 mL) was stirred at 110 ºC for 18 h. The crude product was purified by column chromatography in silica gel, eluting with a mixture 1:3 ethyl acetate/hexanes to provide the title compound as a yellow solid. Yield: 215.0 mg, 92%.

^{1}H NMR (300 MHz, $CDCl_3$): δ 8.07 (s, 1H), 7.76 (d, 1H, *J* = 9.1 Hz), 7.70 (d, 1H, *J* = 8.4 Hz), 7.51 (dd, 1H, *J* = 8.6; 1.6 Hz), 7.33 (dd, 1H, *J* = 9.1; 2.5 Hz), 7.07 (d, 1H, *J* = 2.5 Hz), 3.91 (br t, 4H, *J* = 4.8 Hz), 3.34 (br t, 4H, *J* = 5.0 Hz).

^{13}C NMR (75 MHz, $CDCl_3$): δ 151.3, 136.5, 133.8, 129.6, 127.7, 127.2, 127.1, 120.0, 119.6, 109.1, 106.2, 66.8, 48.8.

ESI-MS (Orbitrap): *m/z* calculated for $C_{15}H_{14}ON_2^+$ [M + H]$^+$ 239.1179, found 239.1177.

6-(Piperidin-1-yl)-2-naphthonitrile (Scheme 3, 5b)

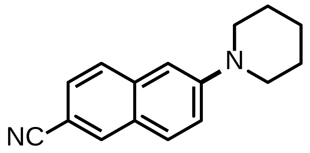

Following the general procedure, a mixture of **2c** (276.3 mg, 1 mmol), piperidine (118.6 μL, 1.2 mmol), NaO*t*Bu (115.3 mg, 1.2 mmol), **1** (2.5 mol%) in *t*BuOH/H_2O 1:1 (2 mL) was stirred at 110 ºC for 18 h. The crude product was purified by column chromatography in silica gel, eluting with a mixture 1:10 ethyl acetate/hexanes to provide the title compound as a light pink solid. Yield: 210.0 mg, 89%.

^{1}H NMR (300 MHz, $CDCl_3$): δ 8.03 (s, 1H), 7.70 (d, 1H, *J* = 9.3 Hz), 7.65 (d, 1H, *J* = 8.4 Hz), 7.46 (dd, 1H, *J* = 8.6; 1.7 Hz), 7.34 (dd, 1H, *J* = 9.1; 2.5 Hz), 7.05 (d, 1H, *J* = 2.6 Hz), 3.36 (br t, 4H, *J* = 5.1 Hz), 1.79-1.60 (m, 6H).

^{13}C NMR (75 MHz, $CDCl_3$): δ 151.8, 136.8, 133.7, 129.4, 127.5, 127.0, 126.5, 120.4, 120.2, 108.9, 105.4, 49.9, 25.7, 24.4.

ESI-MS (Orbitrap): *m/z* calculated for $C_{16}H_{17}N_2^+$ $[M + H]^+$ 237.1386, found 237.1386.

1-(7-Methoxynaphthalen-2-yl)pyrrolidine (Scheme 3, 5c)

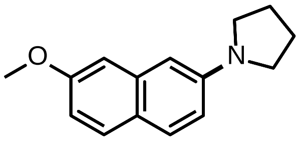

Following the general procedure, a mixture of **2g** (282.3 mg, 1 mmol), pyrrolidine (100,2 µL, 1.2 mmol), Na*Ot*Bu (115.3 mg, 1.2 mmol), **1** (2.5 mol%) in *t*BuOH/H_2O 1:1 (2 mL) was stirred at 110 ºC for 18 h. The crude product was purified by column chromatography in silica gel, eluting with a mixture 1:20 ethyl acetate/hexanes to provide the title compound as a red oil. Yield: 216.1 mg, 95%.

^1H NMR (300 MHz, $CDCl_3$): δ 7.60 (dd, 2H, *J* = 12.2; 8.9 Hz), 6.98 (d, 1H, *J* = 2.4 Hz), 6.84 (dt, 2H, *J* = 8.9; 2.3 Hz); 6.69 (d, 1H, *J* = 2.2 Hz); 3.91 (s, 3H), 3.41 (br t, 4H, *J* = 6.6 Hz), 2.06 (br t, 4H, J_{HH} = 6.7 Hz).

^{13}C NMR (75 MHz, $CDCl_3$): δ 158.3, 146.6, 136.6, 129.3, 128.7, 121.9, 113.9, 113.4, 104.3, 55.3, 47.9, 25.6.

ESI-MS (Orbitrap): *m/z* calculated for $C_{15}H_{18}ON^+$ $[M + H]^+$ 228.1383, found 228.1381.

1-(naphthalen-2-yl)indoline[16] (Scheme 3, 5d)

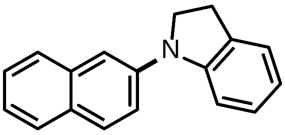

Following the general procedure, a mixture of **2b** (251.3 mg, 1 mmol), indoline (134.6 µL, 1.2 mmol), Na*Ot*Bu (115.3 mg, 1.2 mmol), **1** (2.5 mol%) in *t*BuOH/H_2O 1:1 (2 mL) was stirred at 110 ºC for 18 h. The crude product was purified by column chromatography in silica gel, eluting with hexanes to provide the title compound as a red oil. Yield: 240.3 mg, 98%.

^1H NMR (300 MHz, $CDCl_3$): δ 7.82 (t, 2H, *J* = 9.2 Hz), 7.75 (d, 1H, *J* = 8.3 Hz), 7.60 (dd, 1H, *J* = 8.9; 2.4 Hz), 7.50-7.42 (m, 2H), 7.36 (t, 1H, *J* = 7.5 Hz), 7.30-7.21 (m, 2H), 7.14 (t, 1H, *J* = 7.7 Hz), 6.82 (t, 1H, *J* = 7.3 Hz), 4.09 (t, 2H, *J* = 8.4 Hz, CH_2), 3.20 (t, 2H, *J* = 8.4 Hz).

1-(7-methoxynaphthalen-2-yl)-2-methylpiperidine (Scheme 3, 5e)

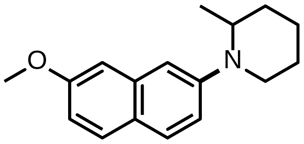

Following the general procedure, a mixture of **2g** (282.3 mg, 1 mmol), 2-methyl piperidine (141 µL, 1.2 mmol), Na*Ot*Bu (115.3 mg, 1.2 mmol), **1** (2.5 mol%) in *t*BuOH/H_2O 1:1 (2 mL) was stirred at 110 ºC for 18 h. The crude product was purified by column chromatography in

silica gel, eluting with a mixture 1:20 ethyl acetate/hexanes to provide the title compound as a light yellow oil. Yield: 212.7 mg, 83%.

^{1}H NMR (300 MHz, $CDCl_3$): δ 7.60 (t, 2H, *J* = 8.5 Hz), 7.19-7.11 (m, 1H), 7.05-6.97 (m, 2H), 6.92 (dd, 1H, *J* = 8.9; 2.5 Hz), 3.90 (s, 3H), 3.39-3.22 (m, 1H), 3.13-3.00 (m, 1H), 1.90-1.50 (m, 7H), 1.03 (d, 3H, *J* = 6.6 Hz).

^{13}C NMR (75 MHz, $CDCl_3$): δ 149.8, 129.2, 128.8, 128.3, 123.6, 117.6, 117.6, 115.4, 111.0, 105.0, 55.2, 51.5, 44.6, 31.6, 26.1, 19.5, 13.7.

ESI-MS (Orbitrap): *m/z* calculated for $C_{17}H_{22}ON^+$ $[M + H]^+$ 256.1696, found 256.1696.

4-(Naphthalen-1-yl)morpholine[17] (Scheme 3, 5f)

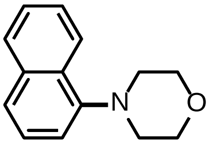

Following the general procedure, a mixture of **2a** (251.3 mg, 1 mmol), morpholine (104.6 µL, 1.2 mmol), NaO*t*Bu (115.3 mg, 1.2 mmol), **1** (2.5 mol%) in *t*BuOH/H_2O 1:1 (2 mL) was stirred at 110 ºC for 18 h. The crude product was purified by column chromatography in silica gel, eluting with a mixture 1:10 ethyl acetate/hexanes to provide the title compound as a light brown solid. Yield: 187.4 mg, 88%.

^{1}H NMR (300 MHz, $CDCl_3$): δ 8.28-8.18 (m, 1H), 7.89-7.78 (m, 1H), 7.58 (d, 1H, *J* = 8.2 Hz), 7.54-7.46 (m, 2H), 7.42 (t, 1H, *J* = 7.5 Hz), 7.10 (d, 1H, *J* = 8.2 Hz), 4.06-3.95 (m, 4H), 3.20-3.07 (m, 4H).

***N*-Methyl-*N*-phenylnaphthalen-1-amine[5] (Scheme 3, 5g)**

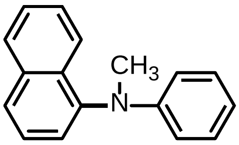

Following the general procedure, a mixture of **2a** (251.3 mg, 1 mmol), *N*-methylaniline (130.0 µL, 1.2 mmol), NaO*t*Bu (115.3 mg, 1.2 mmol), **1** (2.5 mol%) in *t*BuOH/H_2O 1:1 (2 mL) was stirred at 110 ºC for 18 h. The crude product was purified by column chromatography in silica gel, eluting with hexanes to provide the title compound as a yellow solid. Yield: 211.5 mg, 91%.

^{1}H NMR (300 MHz, $CDCl_3$): δ 7.91 (t, 2H, *J* = 7.8 Hz), 7.80 (d, 1H, *J* = 8.2 Hz), 7.55-7.47 (m, 2H), 7.46-7.40 (m, 1H), 7.38 (d, 1H, *J* = 7.2 Hz), 7.17 (t, 2H, *J* = 8.0 Hz), 6.74 (td, 1H, *J* = 7.4; 0.9 Hz), 6.63 (dd, 2H, *J* = 7.9; 0.9 Hz), 3.41 (s, 3H).

_N,N_-Dibenzylnaphthalen-2-amine[18] (Scheme 3, 5h)

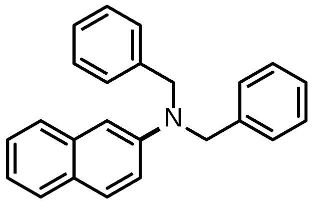

Following the general procedure, a mixture of **2b** (251.3 mg, 1 mmol), dibenzylamine (241.4 μL, 1.2 mmol), Na*Ot*Bu (115.3 mg, 1.2 mmol), **1** (2.5 mol%) in *t*BuOH/H_2O 1:1 (2 mL) was stirred at 110 ºC for 18 h. The crude product was purified by column chromatography in silica gel, eluting with hexanes to provide the title compound as a white solid. Yield: 168.7 mg, 52%.

^{1}H NMR (300 MHz, $CDCl_3$): δ 7.89-7.79 (m, 2H), 7.76-7.70 (m, 1H), 7.61 (d, 1H, *J* = 8.3 Hz), 7.54-7.46 (m, 2H), 7.44-7.32 (m, 8H), 7.30-7.20 (m, 2H), 7.07 (d, 1H, *J* = 2.6 Hz), 4.81 (s, 4H).

_N_-benzylnaphthalen-2-amine[19] (Scheme 3, 5i)

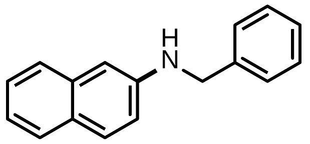

Following the general procedure, a mixture of **2b** (251.3 mg, 1 mmol), benzylamine (131 μL, 1.2 mmol), Na*Ot*Bu (115.3 mg, 1.2 mmol), **1** (2.5 mol%) in *t*BuOH/H_2O 1:1 (2 mL) was stirred at 110 ºC for 18 h. The crude product was purified by column chromatography in silica gel, eluting with a mixture 1:20 ethyl acetate/hexanes to provide the title compound as a light brown solid. Yield: 213.1 mg, 92%.

^{1}H NMR (300 MHz, $CDCl_3$): δ 7.69 (dd, 1H, *J* = 8.1; 0.5 Hz), 7.65 (d, 1H, *J* = 8.9 Hz), 7.61 (dd, 1H, *J* = 8.2; 0.5 Hz), 7.47-7.27 (m, 6H), 7.21 (ddd, 1H, *J* = 8.1; 6.8; 1.2 Hz), 6.93 (dd, 1H, *J* = 8.7; 2.4 Hz), 6.85 (d, 1H, *J* = 2.3 Hz), 4.45 (s, 2H), 4.20 (br s, 1H).

4-(Hexylamino)benzonitrile[20] (Scheme 3, 5j)

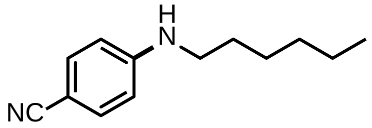

Following the general procedure, a mixture of **2e** (227.2 mg, 1 mmol), n-hexylamine (158.8 μL, 1.2 mmol), Na*Ot*Bu (115.3 mg, 1.2 mmol), **1** (4 mol%) in *t*BuOH/H_2O 1:1 (2 mL) was stirred at 120 ºC for 18 h. The crude product was purified by column chromatography in silica gel, eluting with a mixture 1:7 ethyl acetate/hexanes to provide the title compound as a white solid. Yield: 100.2 mg, 50%.

^{1}H NMR (300 MHz, $CDCl_3$): δ 7.40 (d, 2H, *J* = 8.3 Hz), 6.53 (d, 2H, *J* = 8.3 Hz), 4.19 (br s, 1H), 3.20-2.92 (m, 2H), 1.73-1.04 (m, 8H), 0.90 (t, 3H, *J* = 6.8 Hz).

4-(butylamino)benzonitrile[21] (Scheme 3, 5k)

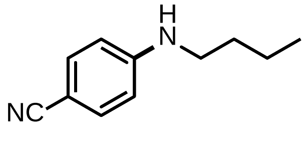

Following the general procedure, a mixture of **2e** (227.2 mg, 1 mmol), n-butylamine (118.8 μL, 1.2 mmol), Na*Ot*Bu (115.3 mg,

1.2 mmol), **1** (4 mol%) in *t*BuOH/H_2O 1:1 (2 mL) was stirred at 120 ºC for 18 h. The crude product was purified by column chromatography in silica gel, eluting with a mixture 1:20 ethyl acetate/hexanes to provide the title compound as a light brown solid. Yield: 70.0 mg, 40%.

^1H NMR (300 MHz, $CDCl_3$): δ 7.41 (d, 2H, *J* = 8.7 Hz), 6.54 (d, 2H, *J* = 8.7 Hz), 4.15 (br s, 1H), 3.15 (q, 2H, *J* = 6.5 Hz), 1,70-1.52 (m, 2H), 1.43 (hex, 2H, *J* = 7.1 Hz), 0.96 (t, 3H, *J* = 7.3 Hz).

N-Hexylnaphthalen-2-amine[22] (Scheme 3, 5l)

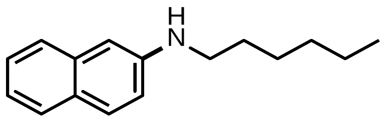

Following the general procedure, a mixture of **2b** (251.3 mg, 1 mmol), n-hexylamine (158.8 μL, 1.2 mmol), NaO*t*Bu (115.3 mg, 1.2 mmol), **1** (3 mol%) in *t*BuOH/H_2O 1:1 (2 mL) was stirred at 110 ºC for 18 h. The crude product was purified by column chromatography in silica gel, eluting with a mixture 1:8 ethyl acetate/hexanes to provide the title compound as a colorless oil. Yield: 92.7 mg, 44%.

^1H NMR (300 MHz, $CDCl_3$): δ 7.69 (d, 1H, *J* = 8.0 Hz), 7.64 (d, 2H, *J* = 8.9 Hz), 7.43-7.33 (m, 1H), 7.25-7.16 (m, 1H), 6.89 (dd, 1H, *J* = 8.8; 2.3 Hz), 6.82 (d, 1H, *J* = 1.9 Hz), 3.78 (br s, 1H), 3.23 (t, 2H, *J* = 7.1 Hz), 1.77-1.61 (m, 2H), 1.54-1.23 (m, 6H), 1.03-0.83 (m, 3H).

N-Butylnaphthalen-2-amine[23] (Scheme 3, 5m)

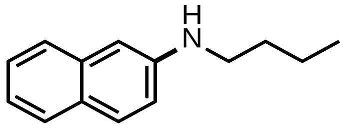

Following the general procedure, a mixture of **2a** (251.3 mg, 1 mmol), n-butylamine (118.8 μL, 1.2 mmol), NaO*t*Bu (115.3 mg, 1.2 mmol), **1** (2.5 mol%) in *t*BuOH/H_2O 1:1 (2 mL) was stirred at 110 ºC for 18 h. The crude product was purified by column chromatography in silica gel, eluting with a mixture 1:10 ethyl acetate/hexanes to provide the title compound as a yellow-brown oil. Yield: 109.8 mg, 55%.

^1H NMR (300 MHz, $CDCl_3$): δ 7.66 (d, 1H, *J* = 7.9 Hz), 7.61 (d, 2H, *J* = 8.6 Hz), 7.40-7.30 (m, 1H), 7.22-7.13 (m, 1H), 6.87 (dd, 1H, *J* = 8.8; 2.3 Hz), 6.80 (d, 1H, *J* = 2.2 Hz), 3.76 (br s, 1H), 3.23 (t, 2H, *J* = 7.1 Hz), 1.76-1.61 (m, 2H), 1.57-1.40 (m, 2H), 0.99 (t, 3H, *J* = 7.3 Hz).

N-(naphthalen-2-yl)pyridyl-3-amine (Scheme 4, 6a)

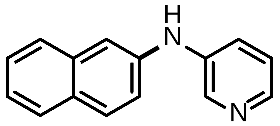

Following the general procedure, a mixture of **2b** (251.3 mg, 1 mmol), 3-aminopyridine (112.7 mg, 1.2 mmol), NaO*t*Bu (115.3 mg, 1.2 mmol), **1** (2.5 mol%) in *t*BuOH/H_2O 1:1 (2 mL) was stirred

at 110 ºC for 18 h. The crude product was purified by column chromatography in silica gel, eluting with a mixture 1:10 ethyl acetate/hexanes to provide the title compound as a brown solid. Yield: 213.6 mg, 97%.

^{1}H NMR (300 MHz, $CDCl_3$): δ 8.48 (br s, 1H), 8.22 (d, 1H, *J* = 4.2 Hz), 7.83-7.72 (m, 2H), 7.67 (d, 1H, *J* = 8.2 Hz), 7.55-7.39 (m, 3H), 7.38-7.29 (m, 1H), 7.25-7.17 (m, 2H), 5.93 (br s, 1H).

^{13}C NMR (75 MHz, $CDCl_3$): δ 142.4, 142.2, 140.4, 140.0, 139.8, 134.6, 129.8, 129.7, 127.9, 127.0, 126.8, 124.2, 124.0, 120.2, 112.9.

ESI-MS (Orbitrap): *m/z* calculated for $C_{15}H_{13}N_2^+$ $[M + H]^+$ 221.1073, found 221.1072.

N-(naphthalen-1-yl)pyridine-2-amine[24] (Scheme 4, 6b)

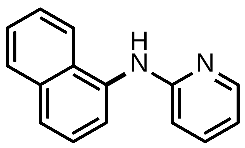

Following the general procedure, a mixture of **2a** (251.3 mg, 1 mmol), 2-aminopyridine (112.8 mg, 1.2 mmol), NaO*t*Bu (115.3 mg, 1.2 mmol), **1** (2.5 mol%) in *t*BuOH/H_2O 1:1 (2 mL) was stirred at 110 ºC for 18 h. The crude product was purified by column chromatography in silica gel, eluting with a mixture 1:7 ethyl acetate/hexanes to provide the title compound as a yellow solid. Yield: 194.6 mg, 88%.

^{1}H NMR (300 MHz, $CDCl_3$): δ 8.19-8.14 (m, 1H), 8.13-8.06 (m, 1H), 7.92-7.88 (m, 1H), 7.72 (d, 1H, *J* = 8.1 Hz), 7.58 (dd, 1H, *J* = 7.4; 1.2 Hz), 7.54-7.44 (m, 3H), 7.40 (ddd, 2H, *J* = 8.5; 7.2; 1.9 Hz), 6.69 (ddd, 1H, *J* = 7.2; 5.0; 0.9 Hz), 6.62 (dt, 1H, *J* = 8.5; 0.8 Hz).

N-(Naphthalen-1-yl)pyrimidin-2-amine[25] (Scheme 4, 6c)

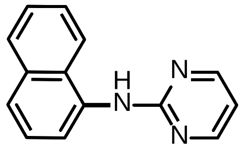

Following the general procedure, a mixture of **2a** (251.3 mg, 1 mmol), 2-aminopyrimidine (114.0 mg, 1.2 mmol), NaO*t*Bu (115.3 mg, 1.2 mmol), **1** (2.5 mol%) in *t*BuOH/H_2O 1:1 (2 mL) was stirred at 110 ºC for 18 h. The crude product was purified by column chromatography in silica gel, eluting with a mixture 1:2 ethyl acetate/hexanes to provide the title compound as a light-yellow solid. Yield: 98.2 mg, 44%.

^{1}H NMR (300 MHz, $CDCl_3$): δ 8.41-8.31 (m, 2H), 8.11-8,03 (m, 1H), 8.01 (d, 1H, *J* = 7.6 Hz), 7.92-7.85 (m, 1H), 7.71 (d, 1H, *J* = 8.1 Hz), 7.57-7.46 (m, 3H), 6.71-6.63 (m, 1H).

N-(7-Methoxynaphthalen-2-yl)pyrimidin-2-amine (Scheme 4, 6d)

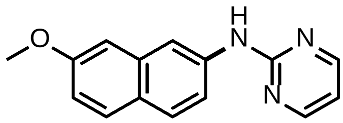

Following the general procedure, a mixture of **2g** (282.3 mg, 1 mmol), 2-aminopyrimidine (114.0 mg, 1.2 mmol), NaO*t*Bu (115.3 mg, 1.2 mmol), **1** (2.5 mol%) in *t*BuOH/H_2O 1:1 (2

mL) was stirred at 110 ºC for 18 h. The crude product was purified by column chromatography in silica gel, eluting with a mixture 1:10 ethyl acetate/hexanes to provide the title compound as a white solid. Yield: 235.9 mg, 94%.

^{1}H NMR (300 MHz, $CDCl_3$): δ 8.48 (d, 2H, *J* = 4.0 Hz), 8.23 (d, 1H, *J* = 2.1 Hz), 7.69 (dd, 2H, *J* = 15.1; 8.8 Hz), 7.41 (dt, 1H, *J* = 8.8; 1.5 Hz), 7.12 (d, 1H, *J* = 2.5 Hz), 7.03 (ddd, 1H, *J* = 8.9; 2.5; 0.5 Hz), 6.75 (td, 1H, *J* = 4.8; 1.3 Hz), 3.92 (s, 3H).

^{13}C NMR (75 MHz, $CDCl_3$): δ 158.3, 158.2, 137.6, 135.7, 129.2, 128.5, 125.5, 118.1, 117.2, 114.3, 114.3, 112.8, 105.7, 55.4.

ESI-MS (Orbitrap): *m/z* calculated for $C_{15}H_{14}ON_3^+$ $[M + H]^+$ 252.1131, found 252.1132.

N-(Pyrazin-2-yl)quinoline-6-amine[26] (Scheme 4, 6e)

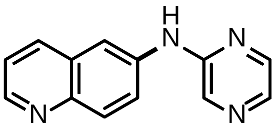

Following the general procedure, a mixture of **2d** (252.3 mg, 1 mmol), 2-aminopyrazine (114.0 mg, 1.2 mmol), NaO*t*Bu (115.3 mg, 1.2 mmol), **1** (2.5 mol%) in *t*BuOH/H_2O 1:1 (2 mL) was stirred at 110 ºC for 18 h. The crude product was purified by column chromatography in silica gel, eluting with ethyl acetate to provide the title compound as a light brown solid. Yield: 154.8 mg, 70%.

^{1}H NMR (300 MHz, $CDCl_3$): δ 8.81 (dd, 1H, *J* = 4.2; 1.6 Hz), 8.33 (d, 1H, *J* = 1,3 Hz), 8.24-8.20 (m, 1H), 8.19 (d, 1H, *J* = 2,5 Hz), 8.14-8.03 (m, 3H), 7.64 (dd, 1H, *J* = 9.0; 2.5 Hz), 7.38 (dd, 1H, *J* = 8.3; 4.3 Hz), 6.98 (br s, 1H).

ESI-MS (Orbitrap): *m/z* calculated for $C_{13}H_{11}N_4^+$ $[M + H]^+$ 223.0978, found 223.0980.

N-(Naphthalen-1yl)pyrazin-2-amine (Scheme 4, 6f)

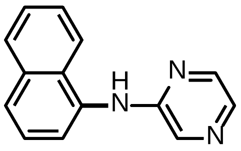

Following the general procedure, a mixture of **2a** (251.3 mg, 1 mmol), 2-aminopyrazine (114.0 mg, 1.2 mmol), NaO*t*Bu (115.3 mg, 1.2 mmol), **1** (2.5 mol%) in *t*BuOH/H_2O 1:1 (2 mL) was stirred at 110 ºC for 18 h. The crude product was purified by column chromatography in silica gel, eluting with a mixture 1:2 ethyl acetate/hexanes to provide the title compound as a yellow solid. Yield: 191.2 mg, 87%.

^{1}H NMR (300 MHz, $CDCl_3$): δ 8.13-7.98 (m, 3H), 7.97-7.86 (m, 2H), 7.76 (d, 1H, *J* = 8.2 Hz), 7.62 (d, 1H, *J* = 7.3 Hz), 7.58-7.42 (m, 3H).

^{13}C NMR (75 MHz, $CDCl_3$): δ 154.2, 142.2, 134.9, 132.4, 129.3, 128.8, 127.8, 126.6, 126.3, 126.0, 122.2, 121.1.

ESI-MS (Orbitrap): *m/z* calculated for $C_{14}H_{12}N_3^+$ $[M + H]^+$ 222.1026, found 222.1025.

N-(7-Methoxynaphthalen-2-yl)oxazol-2-amine (Scheme 4, 6g)

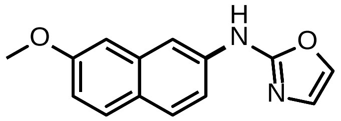

Following the general procedure, a mixture of **2g** (282.3 mg, 1 mmol), 2-aminooxazole (100.9 mg, 1.2 mmol), NaO*t*Bu (115.3 mg, 1.2 mmol), **1** (2.5 mol%) in *t*BuOH/H_2O 1:1 (2 mL) was stirred at 110 ºC for 18 h. The crude product was purified by column chromatography in silica gel, eluting with a mixture 1:2 ethyl acetate/hexanes to provide the title compound as a dark brown solid. Yield: 180.7 mg, 75%.

^{1}H NMR (300 MHz, $CDCl_3$): δ 7.74-7.61 (m, 3H), 7.44 (d, 1H, *J* = 2.1 Hz), 7.20-7.09 (m, 2H), 7.04-6.94 (m, 2H), 3.90 (s, 3H).

^{13}C NMR (75 MHz, $CDCl_3$): δ 158.5, 141.2, 136.3, 136.0, 129.3, 129.2, 125.1, 124.8, 118.1, 116.6, 112.1, 105.1, 55.4.

ESI-MS (Orbitrap): *m/z* calculated for $C_{14}H_{13}O_2N_2^+$ $[M + H]^+$ 241.0972, found 241.0969.

6-(benzo[*d*]oxazol-2-ylamino)-2-naphthonitrile (Scheme 4, 6h)

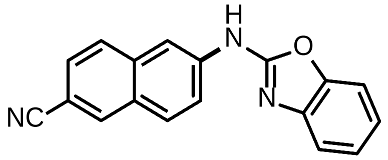

Following the general procedure, a mixture of **2c** (276.3 mg, 1 mmol), 2-aminobenzoxazole (161.0 mg, 1.2 mmol), NaO*t*Bu (115.3 mg, 1.2 mmol), **1** (2.5 mol%) in *t*BuOH/H_2O 1:1 (2 mL) was stirred at 110 ºC for 18 h. The crude product was purified by column chromatography in silica gel, eluting with a mixture 1:5 ethyl acetate/hexanes to provide the title compound as a yellow solid. Yield: 271.4 mg, 95%.

^{1}H NMR (300 MHz, DMSO-d_6): δ 11.12 (br s, 1H), 8.53 (d, 1H, *J* = 1.9 Hz), 8.46 (d, 1H, *J* = 0.7 Hz), 8.04 (t, 2H, *J* = 8.7 Hz), 7.86 (dd, 1H, *J* = 8.9; 2.1 Hz), 7.71 (dd, 1H, *J* = 8.5; 1.6 Hz), 7.59-7.50 (m, 2H), 7.27 (td, 1H, *J* = 7.6; 1.2 Hz), 7.19 (td, 1H, *J* = 7.7; 1.3 Hz).

^{13}C NMR (75 MHz, DMSO-d_6): δ 157.5, 147.0, 142.1, 139.3, 135.5, 134.0, 129.7, 128.4, 127.9, 127.0, 124.2, 122.3, 120.5, 119.4, 117.1, 112.3, 109.2, 106.1.

ESI-MS (Orbitrap): *m/z* calculated for $C_{18}H_{12}ON_3^+$ $[M + H]^+$ 286.0975, found 286.0975.

7-Methoxy-*N*-(pyridine-4-ylmethyl)naphthalen-2-amine (Scheme 4, 6i)

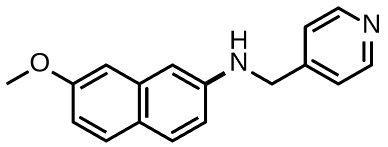

Following the general procedure, a mixture of **2g** (282.3 mg, 1 mmol), 4-(aminomethyl)pyridine (122.8 µL, 1.2 mmol), NaO*t*Bu (115.3 mg, 1.2 mmol), **1** (2.5 mol%) in *t*BuOH/H_2O 1:1 (2 mL) was stirred at 110 ºC for 18 h. The crude product was purified by

column chromatography in silica gel, eluting with ethyl acetate to provide the title compound as a light brown solid. Yield: 253.4 mg, 96%.

^{1}H NMR (300 MHz, $CDCl_3$): δ 8.55 (d, 2H, *J* = 5.9 Hz), 7.56 (d, 2H, *J* = 9.0 Hz), 7.29 (d, 2H, *J* = 6.0 Hz), 6.92-6.83 (m, 2H), 6.75 (dd, 1H, *J* = 8.7; 2.4 Hz), 6.60 (d, 1H, *J* = 2.3 Hz), 4.42 (s, 2H), 3.84 (s, 3H).

^{13}C NMR (75 MHz, $CDCl_3$): δ 158.4, 150.0, 148.9, 145.8, 136.3, 129.2, 129.0, 123.1, 122.1, 115.2, 114.8, 104.6, 104.3, 55.2, 47.0.

ESI-MS (Orbitrap): *m/z* calculated for $C_{17}H_{17}ON_2^+$ $[M + H]^+$ 236.1335, found 236.1335.

_N_-(Pyridin-2-ylmethyl)naphthalen-1-amine[27] (Scheme 4, 6j)

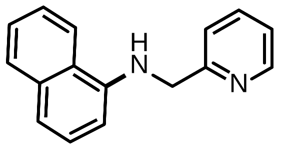

Following the general procedure, a mixture of **2a** (251.3 mg, 1 mmol), 2-(aminomethyl)pyridine (122.8 μL, 1.2 mmol), NaO*t*Bu (115.3 mg, 1.2 mmol), **1** (2.5 mol%) in *t*BuOH/H_2O 1:1 (2 mL) was stirred at 110 ºC for 18 h. The crude product was purified by column chromatography in silica gel, eluting with a mixture 1:20 ethyl acetate/hexanes to provide the title compound as a yellow oil. Yield: 200.4 mg, 86%.

^{1}H NMR (300 MHz, $CDCl_3$): δ 8.67 (ddd, 1H, *J* = 4.9; 1.7; 0.9 Hz), 8.07-7.98 (m, 1H), 7.90-7.81 (m, 1H), 7.59 (td, 1H, *J* = 7.7; 1.8 Hz), 7.53-7.48 (m, 2H), 7.44-7.27 (m, 3H), 7.21-7.14 (m, 1H), 6.62 (dd, 1H, *J* = 7.4; 1.1 Hz), 5.79 (br s, 1H), 4.62 (s, 2H).

1-(Naphthalen-2-yl)-1*H*-pyrrole[28] (Scheme 4, 7a)

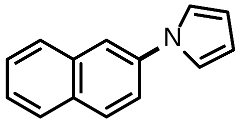

Following the general procedure, a mixture of **2b** (251.3 mg, 1 mmol), 1H-pyrrole (83.2 μL, 1.2 mmol), NaO*t*Bu (115.3 mg, 1.2 mmol), **1** (2.5 mol%) in *t*BuOH/H_2O 1:1 (2 mL) was stirred at 110 ºC for 18 h. The crude product was purified by column chromatography in silica gel, eluting with hexanes to provide the title compound as a light brown solid. Yield: 157.4 mg, 81%.

^{1}H NMR (300 MHz, $CDCl_3$): δ 8.00-7.80 (m, 4H), 7.64 (dd, 1H, *J* = 8.9; 2.2 Hz), 7.60-7.48 (m, 2H), 7.30 (d, 2H, *J* = 1.4 Hz), 6.51 (d, 2H, *J* = 1.9 Hz).

6-(1*H*-Pyrazol-1-yl)-2-naphthonitrile. (Scheme 4, 7b)

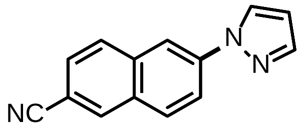

Following the general procedure, a mixture of **2c** (251.3 mg, 1 mmol), 1H-pyrazole (81.4 mg, 1.2 mmol), NaO*t*Bu (115.3 mg, 1.2 mmol), **1** (5 mol%) in *t*BuOH/H_2O 1:1 (2 mL) was stirred at 110 ºC for 18 h. The crude product was purified by column chromatography in silica gel,

eluting with a mixture 1:4 ethyl acetate/hexanes to provide the title compound as a light yellow solid. Yield: 115.0 mg, 53%.

^{1}H NMR (300 MHz, $CDCl_3$): δ 8.24 (br s, 1H), 8.19 (d, 1H, *J* = 1.9 Hz), 8.10 (dd, 1H, *J* = 2.5; 0.5 Hz), 8.05 (dd, 1H, *J* = 8.9; 2.1 Hz), 8.01 (br s, 1H), 7.99-7.92 (m, 1H), 7.81 (d, 1H, *J* = 1.5 Hz), 7.65 (dd, 1H, *J* = 8.5; 1.6 Hz), 6.56 (dd, 1H, *J* = 2.5; 1.8 Hz).

^{13}C NMR (75 MHz, $CDCl_3$): δ 142.2, 140.1, 135.4, 134.1, 130.7, 130.3, 129.3, 127.7, 127.1, 120.2, 119.2, 116.0, 108.7.

ESI-MS (Orbitrap): *m/z* calculated for $C_{14}H_{10}N_3^+$ $[M + H]^+$ 220.0869, found 220.0867.

9-(Naphthalen-2-yl)-9*H*-carbazole[29] (Scheme 4, 7c)

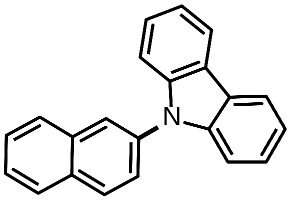

Following the general procedure, a mixture of **2b** (251.3 mg, 1 mmol), carbazole (200.5 mg, 1.2 mmol), NaO*t*Bu (115.3 mg, 1.2 mmol), **1** (2.5 mol%) in *t*BuOH/H_2O 1:1 (2 mL) was stirred at 110 ºC for 18 h. The crude product was purified by column chromatography in silica gel, eluting with hexanes to provide the title compound as a white solid. Yield: 259.6 mg, 89%.

^{1}H NMR (300 MHz, $CDCl_3$): δ 8.21 (d, 2H, *J* = 7.7 Hz), 8.08 (d, 2H, *J* = 8.9 Hz), 7.99 (dd, 1H, *J* = 6.1; 3.4 Hz), 7.93 (dd, 1H, *J* = 6.1; 3.4 Hz), 7.69 (dd, 1H, *J* = 8.6; 2.1 Hz), 7.60 (dt, 2H, *J* = 9.3; 3.6 Hz), 7.52-7.39 (m, 4H), 7.34 (t, 2H, *J* = 7.2 Hz).

^{13}C NMR (75 MHz, $CDCl_3$): δ 141.2, 135.4, 134.2, 132.6, 130.0, 128.1, 128.0, 127.0, 126.6, 126.1, 125.6, 125.5, 123.6, 120.5, 120.2, 110.0.

1-(Naphthalen-2-yl)-1*H*-indole[30] (Scheme 4, 7d)

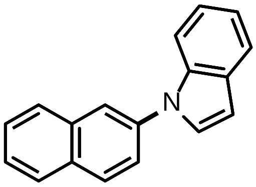

Following the general procedure, a mixture of **2b** (251.3 mg, 1 mmol), indole (140.6 mg, 1.2 mmol), NaO*t*Bu (115.3 mg, 1.2 mmol), **1** (2.5 mol%) in *t*BuOH/H_2O 1:1 (2 mL) was stirred at 110 ºC for 18 h. The crude product was purified by column chromatography in silica gel, eluting with hexanes to provide the title compound as a white solid. Yield: 235.4 mg, 96%.

^{1}H NMR (300 MHz, $CDCl_3$): δ 8.00 (d, 1H, *J* = 8.5 Hz), 7.97-7.87 (m, 3H), 7.74 (dd, 1H, *J* = 7.1; 1.5 Hz), 7.71-7.63 (m, 2H), 7.61-7.51 (m, 2H), 7.46 (d, 1H, *J* = 3.3 Hz), 7.31-7.18 (m, 2H), 6.75 (dd, 1H, *J* = 3.3; 0.8 Hz).

1-(7-Methoxynaphthalen-2-yl)-3-methyl-1*H*-indole (Scheme 4, 7e)

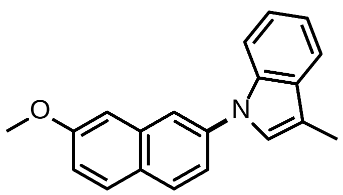

Following the general procedure, a mixture of **2g** (282.3 mg, 1 mmol), 3-methylindole (157.4 mg, 1.2 mmol), NaO*t*Bu (115.3 mg, 1.2 mmol), **1** (2.5 mol%) in *t*BuOH/H_2O 1:1 (2 mL) was stirred at 110 ºC for 18 h. The crude product was purified by column chromatography in silica gel, eluting with a mixture 1:20 ethyl acetate/hexanes to provide the title compound as a white solid. Yield: 252.0 mg, 87%.

^{1}H NMR (300 MHz, $CDCl_3$): δ 7.99-7.80 (m, 3H), 7.77 (d, 2H, *J* = 7.1 Hz), 7.56 (d, 1H, *J* = 8.5 Hz), 7.43-7.15 (m, 5H), 4.00 (s, 3H), 2.52 (s, 3H).

^{13}C NMR (75 MHz, $CDCl_3$): δ 158.6, 138.2, 136.3, 135.4, 130.1, 129.5, 129.3, 127.2, 125.8, 122.6, 120.9, 120.4, 120.0, 119.4, 118.8, 113.2, 110.7, 105.8, 55.5, 9.8.

ESI-MS (Orbitrap): *m/z* calculated for $C_{20}H_{18}ON^+$ $[M + H]^+$ 288.1383, found 288.1385.

1-(7-Methoxynaphthalen-2-yl)-1*H*-indole (Scheme 4, 7f)

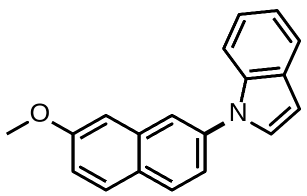

Following the general procedure, a mixture of **2g** (282.3 mg, 1 mmol), indole (140.6 mg, 1.2 mmol), NaO*t*Bu (115.3 mg, 1.2 mmol), **1** (2.5 mol%) in *t*BuOH/H_2O 1:1 (2 mL) was stirred at 110 ºC for 18 h. The crude product was purified by column chromatography in silica gel, eluting with hexanes to provide the title compound as a pink solid. Yield: 246.7 mg, 90%.

^{1}H NMR (300 MHz, $CDCl_3$): δ 7.91 (d, 1H, *J* = 8.6 Hz), 7.86-7.78 (m, 2H), 7.76-7.70 (m, 1H), 7.69-7.64 (m, 1H), 7.52 (dt, 1H, *J* = 8.6; 2.3 Hz), 7.45 (t, 1H, *J* = 3.0 Hz), 7.28-7.16 (m, 4H), 6.76-6.71 (m, 1H), 3.96 (s, 3H).

^{13}C NMR (75 MHz, $CDCl_3$): δ 158.5, 137.9, 136.1, 135.2, 129.4, 129.4, 129.3, 128.1, 127.3, 122.4, 121.2, 120.9, 120.9, 120.4, 119.0, 110.7, 105.7, 103.7, 55.4.

ESI-MS (Orbitrap): *m/z* calculated for $C_{19}H_{16}ON^+$ $[M + H]^+$ 274.1226, found 274.1229.

6-(2-Methyl-1*H*-indol-1-yl)-2-naphthonitrile (Scheme 4, 7g)

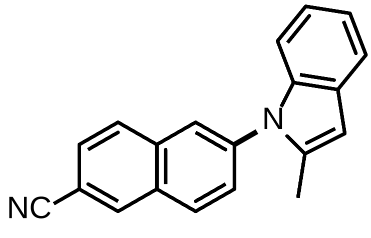

Following the general procedure, a mixture of **2c** (276.3 mg, 1 mmol), 2-methylindole (157.7 mg, 1.2 mmol), NaO*t*Bu (115.3 mg, 1.2 mmol), **1** (2.5 mol%) in *t*BuOH/H_2O 1:1 (2 mL) was stirred at 110 ºC for 18 h. The crude product was purified by column chromatography in

silica gel, eluting with hexanes to provide the title compound as a white solid. Yield: 180.0 mg, 63%.

^{1}H NMR (300 MHz, $CDCl_3$): δ 8.33 (s, 1H), 8.06 (d, 1H, *J* = 8.7 Hz), 7.96 (d, 1H, *J* = 8.5 Hz), 7.90 (d, 1H, *J* = 1.8 Hz), 7.71 (dd, 1H, *J* = 8.5; 1.6 Hz), 7.67-7.57 (m, 2H), 7.25-7.09 (m, 3H), 6.51 (br s, 1H), 2.39 (s, 3H).

^{13}C NMR (75 MHz, $CDCl_3$): δ 138.6, 138.2, 136.9, 135.2, 134.1, 131.2, 130.1, 129.3, 128.6, 127.9, 127.4, 126.2, 121.7, 120.7, 120.0, 119.0, 110.1, 109.9, 102.7, 13.6.

ESI-MS (Orbitrap): *m/z* calculated for $C_{20}H_{15}N_2^+$ $[M + H]^+$ 283.1230, found 283.1232.

6-(3-Methyl-1*H*-indol-1-yl)-2- naphthonitrile (Scheme 4, 7h)

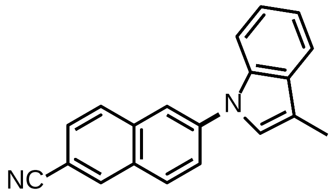

Following the general procedure, a mixture of **2c** (276.3 mg, 1 mmol), 3-methylindole (157.6 mg, 1.2 mmol), NaO*t*Bu (115.3 mg, 1.2 mmol), **1** (2.5 mol%) in *t*BuOH/H_2O 1:1 (2 mL) was stirred at 110 ºC for 18 h. The crude product was purified by column chromatography in silica gel, eluting with a mixture 1:10 ethyl acetate/hexanes to provide the title compound as a white solid. Yield: 206.7 mg, 73%.

^{1}H NMR (300 MHz, $CDCl_3$): δ 8.27 (br s, 1H), 8.02 (dd, 1H, *J* = 8.7; 4.9 Hz), 7.97-7.88 (m, 2H), 7.85-7.76 (m, 1H), 7.72-7.62 (m, 3H), 7.34-7.21 (m, 3H), 2.45 (s, 3H).

^{13}C NMR (75 MHz, $CDCl_3$): δ 140.4, 135.9, 135.6, 134.1, 130.5, 130.3, 130.2, 128.9, 127.6, 125.2, 124.6, 123.1, 120.7, 120.5, 119.7, 119.3, 114.6, 110.5, 109.1, 9.8.

ESI-MS (Orbitrap): *m/z* calculated for $C_{20}H_{14}N_2Na^+$ $[M + Na]^+$ 305.1049, found 305.1048.

Methyl-6-(2,3-dimethyl-1*H*-indol-1-yl)-2-naphthoate (Scheme 4, 7i)

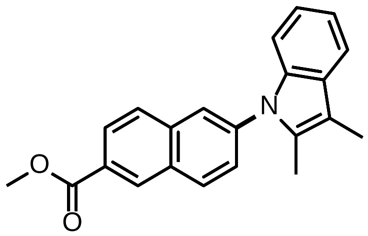

Following the general procedure, a mixture of **2h** (309.3 mg, 1 mmol), 2,3-dimethylindole (174.2 mg, 1.2 mmol), NaO*t*Bu (115.3 mg, 1.2 mmol), **1** (2.5 mol%) in *t*BuOH/H_2O 1:1 (2 mL) was stirred at 110 ºC for 18 h. The crude product was purified by column chromatography in silica gel, eluting with a mixture 1:7 ethyl acetate/hexanes to provide the title compound as a yellow solid. Yield: 135 mg, 41%.

^{1}H NMR (300 MHz, $CDCl_3$): δ 8.70 (br s, 1H), 8.21-8.06 (m, 3H), 7.97-7.88 (m, 1H), 7.85 (d, 1H, *J* = 2.0 Hz), 7.60-7.48 (m, 2H), 7.20-7.07 (m, 3H), 4.02 (s, 3H), 2.36 (s, 3H), 2.30 (s, 3H).

^{13}C NMR (75 MHz, $CDCl_3$): δ 167.2 (C=O), 138.2, 137.4, 136.0, 132.8, 131.4, 131.0, 130.8, 129.2, 128.2, 127.1, 126.3, 125.9, 121.6, 120.6, 120.0, 118.2, 109.8, 109.0, 52.5, 11.3, 9.0.

ESI-MS (Orbitrap): *m/z* calculated for $C_{22}H_{20}O_2N^+$ $[M + H]^+$ 330.1489, found 330.1490.

1-(Naphthalen-2-yl)-2-phenyl-1*H*-indole (Scheme 4, 7j)

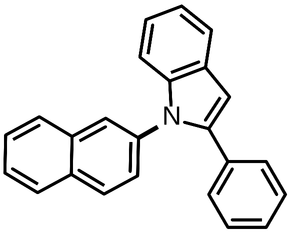

Following the general procedure, a mixture of **2b** (251.3 mg, 1 mmol), 2-phenyl indole (231.8 mg, 1.2 mmol), NaO*t*Bu (115.3 mg, 1.2 mmol), **1** (2.5 mol%) in *t*BuOH/H_2O 1:1 (2 mL) was stirred at 110 ºC for 18 h. The crude product was purified by column chromatography in silica gel, eluting with hexanes to provide the title compound as a light yellow solid. Yield: 190.6 mg, 60%.

^1H NMR (300 MHz, $CDCl_3$): δ 7.91-7.79 (m, 4H), 7.75-7.69 (m, 1H), 7.54 (dt, 2H, J = 9.5; 3.2 Hz), 7.37-7.28 (m, 4H), 7.23-7.16 (m, 5H), 6.86 (s, 1H).

^{13}C NMR (75 MHz, $CDCl_3$): δ 140.9, 136.1, 133.6, 132.6, 132.2, 129.2, 128.9, 128.5, 128.4, 128.2, 127.9, 127.9, 127.3, 126.7, 126.4, 126.1, 122.4, 120.8, 120.6, 110.7, 103.9.

ESI-MS (Orbitrap): *m/z* calculated for $C_{24}H_{18}N^+$ $[M + H]^+$ 320.1434, found 320.1431.

***N*-(Naphthalen-1-yl)benzamide[31] (Scheme 5, 8a)**

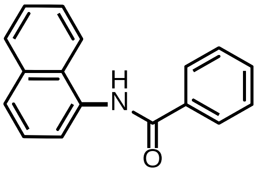

Following the general procedure, a mixture of **2a** (251.3 mg, 1 mmol), benzamide (145.2 mg, 1.2 mmol), NaO*t*Bu (115.3 mg, 1.2 mmol), **1** (3.5 mol%) in *t*BuOH/H_2O 1:1 (2 mL) was stirred at 110 ºC for 18 h. The crude product was purified by column chromatography in silica gel, eluting with a mixture 1:5 ethyl acetate/hexanes to provide the title compound as a light pink solid. Yield: 230.8 mg, 93%.

^1H NMR (300 MHz, $CDCl_3$): δ 8.24 (br s, 1H), 8.08-7.95 (m, 3H), 7.94-7.85 (m, 2H), 7.75 (d, 1H, J = 8.2 Hz), 7.64-7.45 (m, 6H).

4-Methoxy-*N*-(naphthalen-1-yl)benzamide[32] (Scheme 5, 8b)

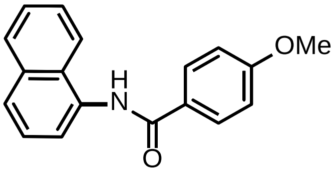

Following the general procedure, a mixture of **2a** (251.3 mg, 1 mmol), 4-methoxybenzamide (181.2 mg, 1.2 mmol), NaO*t*Bu (115.3 mg, 1.2 mmol), **1** (3.5 mol%) in *t*BuOH/H_2O 1:1 (2 mL) was stirred at 110 ºC for 18 h. The crude product was purified by column chromatography in silica gel, eluting with a mixture 1:4 ethyl acetate/hexanes to provide the title compound as a white solid. Yield: 250.2 mg, 90%.

^1H NMR (300 MHz, $CDCl_3$): δ 8.13 (br s, 1H), 8.03 (d, 1H, *J* = 7.4 Hz), 7.96 (dt, 2H, *J* = 8.9; 2.1 Hz), 7.94-7.86 (m, 2H), 7.74 (d, 1H, *J* = 8.2 Hz), 7.58-7.47 (m, 3H), 7.02 (dt, 2H, *J* = 8.8; 2.1 Hz), 3.90 (s, 3H).

_N_-(Naphthalen-1-yl)-4-(trifluoromethyl)benzamide (8c)

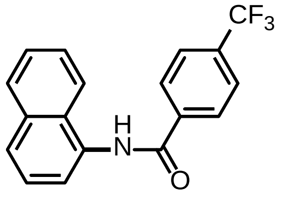

Following the general procedure, a mixture of **2a** (251.3 mg, 1 mmol), 4-(trifluoromethyl)benzamide (227.2 mg, 1.2 mmol), NaO*t*Bu (115.3 mg, 1.2 mmol), **1** (3.5 mol%) in *t*BuOH/H_2O 1:1 (2 mL) was stirred at 110 ºC for 18 h. The crude product was purified by column chromatography in silica gel, eluting with a mixture 1:4 ethyl acetate/hexanes to provide the title compound as a white solid. Yield: 276.8 mg, 88%.

^1H NMR (300 MHz, $CDCl_3$): δ 8.27 (br s, 1H), 8.06 (d, 2H, *J* = 8.0 Hz), 7.99-7.82 (m, 3H), 7.76 (t, 3H, *J* = 6.9 Hz), 7.59-7.43 (m, 3H).

^{13}C NMR (75 MHz, $CDCl_3$): δ 165.3 (C=O), 138.2, 134.3, 133.8 (q, J_{CF} = 33 Hz), 132.1, 129.1 (2C), 127.9, 127.7, 126.8, 126.8, 126.4, 126.0 (q, J_{CF} = 4 Hz, 2C), 125.9, 123.8 (q, J_{CF} = 272 Hz), 121.8, 120.8.

4-Fluoro-_N_-(naphthalen-2-yl)benzamide (Scheme 5, 8d)

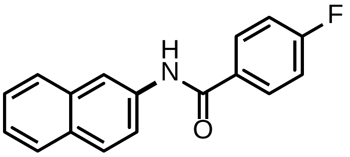

Following the general procedure, a mixture of **2b** (251.3 mg, 1 mmol), 4-fluorobenzamide (167.2 mg, 1.2 mmol), NaO*t*Bu (115.3 mg, 1.2 mmol), **1** (3.5 mol%) in *t*BuOH/H_2O 1:1 (2 mL) was stirred at 110 ºC for 18 h. The crude product was purified by column chromatography in silica gel, eluting with a mixture 1:5 ethyl acetate/hexanes to provide the title compound as a white solid. Yield: 248.6 mg, 94%.

^1H NMR (300 MHz, CDCl*3*): δ 8.31 (d, 1H, *J* = 2.2 Hz), 8.00-7.88 (m, 3H), 7.87-7.76 (m, 3H), 7.58 (dd, 1H, *J* = 8.8; 2.2 Hz), 7.46 (dddd, 2H, *J* = 9.4; 8.2; 6.9; 1.6 Hz), 7.20 (br t, 2H, *J* = 8.7 Hz).

^{13}C NMR (75 MHz, $CDCl_3$): δ 165.1 (d, J_{CF} = 252 Hz), 164.9 (C=O), 135.4, 134.0, 131.3 (d, J_{CF} = 3 Hz), 131.0, 129.6, 129.5, 129.1, 127.9, 127.8, 126.8, 125.4, 120.2, 117.3, 116.1 (d, J_{CF} = 22 Hz).

ESI-MS (Orbitrap): *m/z* calculated for $C_{17}H_{12}ONFNa^+$ $[M + Na]^+$ 288.0795, found 288.0797.

_N_-(7-Methoxynaphthalen-2-yl)formamide (Scheme 5, 8e)

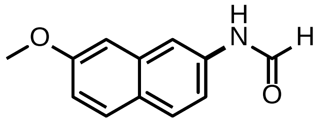

Following the general procedure, a mixture of **2g** (282.3 mg, 1 mmol), formamide (47.6 µL, 1.2 mmol), NaO*t*Bu (115.3 mg, 1.2 mmol), **1** (3.5 mol%) in *t*BuOH/H_2O 1:1 (2 mL) was stirred at 110 ºC for 18 h. The crude product was purified by column chromatography in silica gel, eluting with a mixture 1:10 ethyl acetate/hexanes to provide the title compound as a white solid. Yield: 66.0 mg, 33%.

^{1}H NMR (300 MHz, $CDCl_3$): δ 7.78 (t, 3H, *J* = 8.5 Hz), 7.53-7.42 (m, 1H), 7.37 (ddd, 1H, *J* = 8.1; 6.9; 1.3 Hz), 7.23-7.14 (m, 2H), 3.94 (s, 3H).

^{13}C NMR (75 MHz, $CDCl_3$): δ 157.8, 134.7, 129.5, 129.1, 127.8, 126.9, 126.5, 125.8, 123.7, 118.8, 106.0, 55.4.

ESI-MS (Orbitrap): *m/z* calculated for $C_{12}H_{11}ON_2Na^+$ $[M + Na]^+$ 224.0763, found 224.0766.

7. NMR spectra of compounds.

^{1}H NMR spectrum of 2-naphthyl *N*,*N*-dimethylsulfamate, 2b, (300 MHz, $CDCl_3$, 298 K)

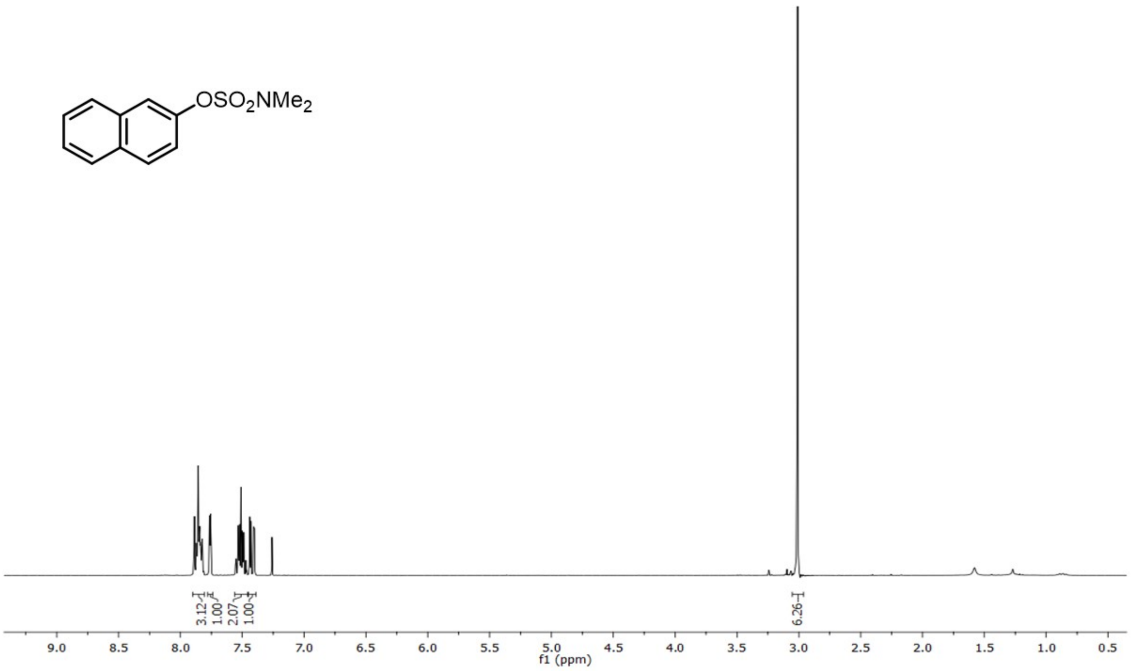

^{13}C{^{1}H} NMR spectrum of 2-naphthyl *N*,*N*-dimethylsulfamate, 2b, (75 MHz, $CDCl_3$, 298 K)**.**

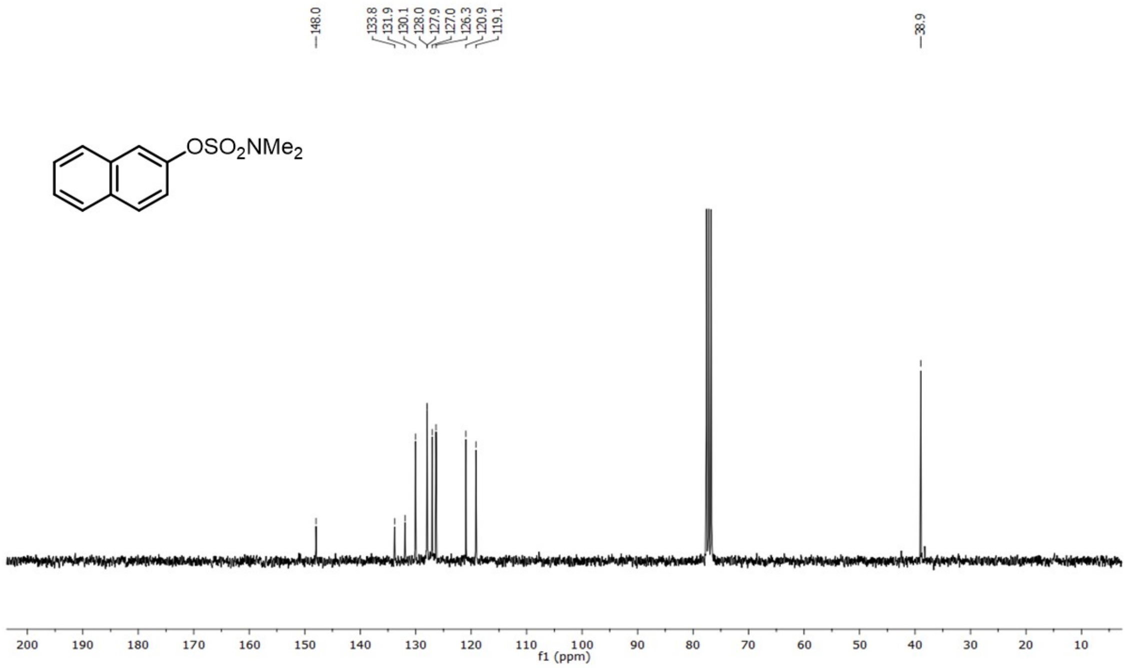

^{1}H NMR spectrum of 6-cyano-2-naphthyl-*N*,*N*-dimetylsulfamate, 2c, (300 MHz, $CDCl_3$, 298 K)

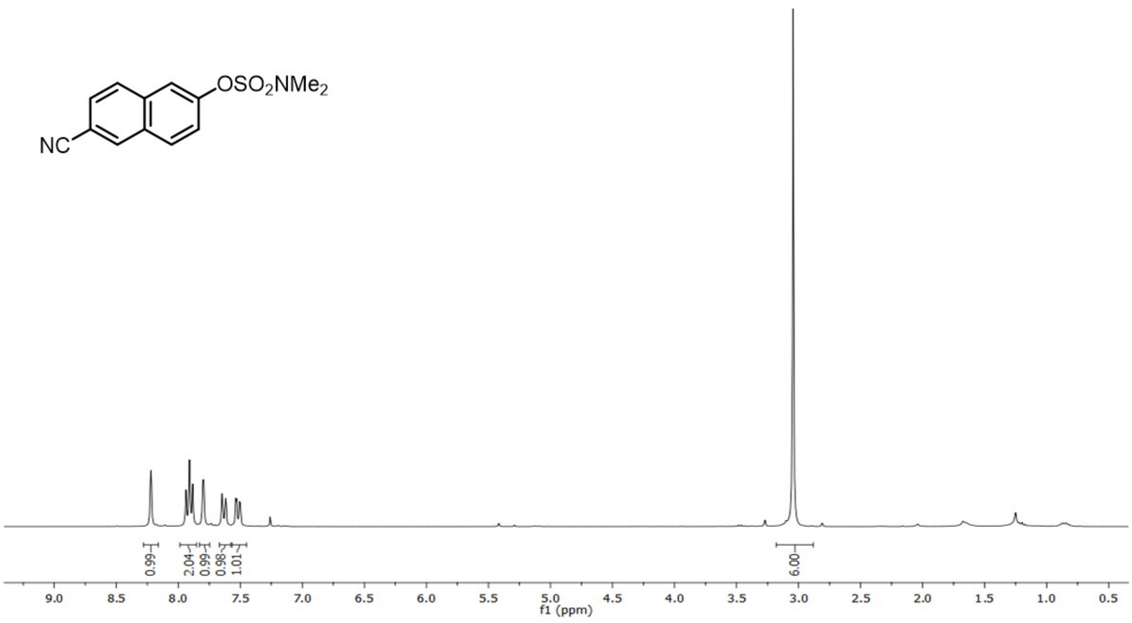

^{13}C{^{1}H} NMR spectrum of 6-cyano-2-naphthyl-*N*,*N*-dimetylsulfamate, 2c, (75 MHz, $CDCl_3$, 298 K)

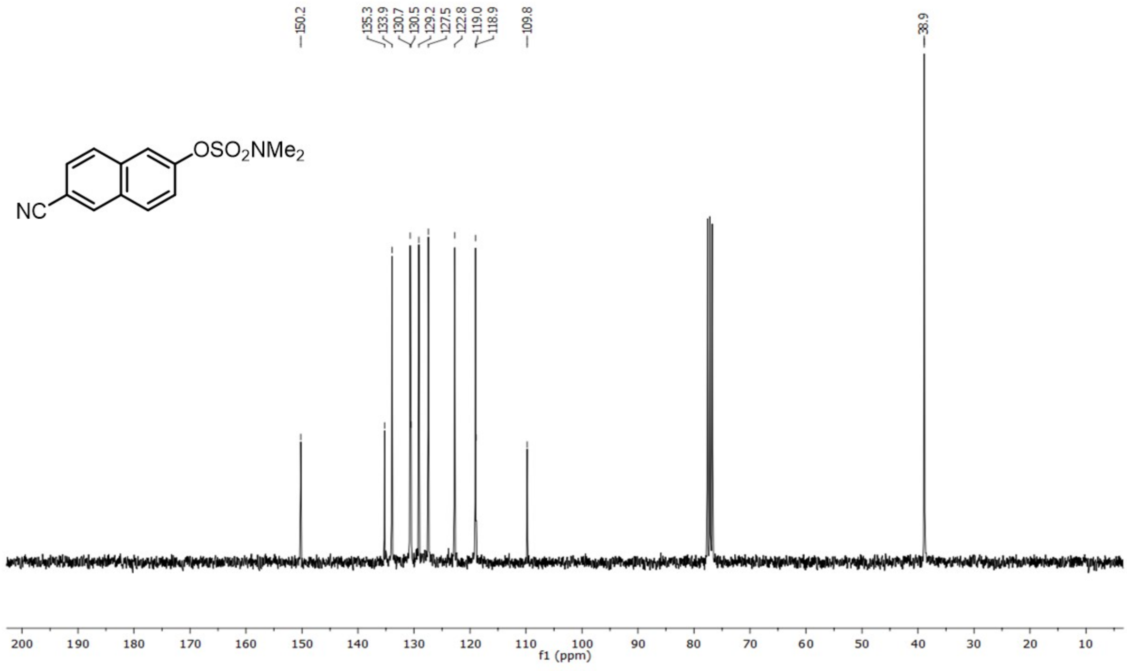

^{1}H NMR spectrum of 7-methoxynaphthalen-2-yl-dimethylsulfamate, 2g (300 MHz, $CDCl_3$, 298 K)**.**

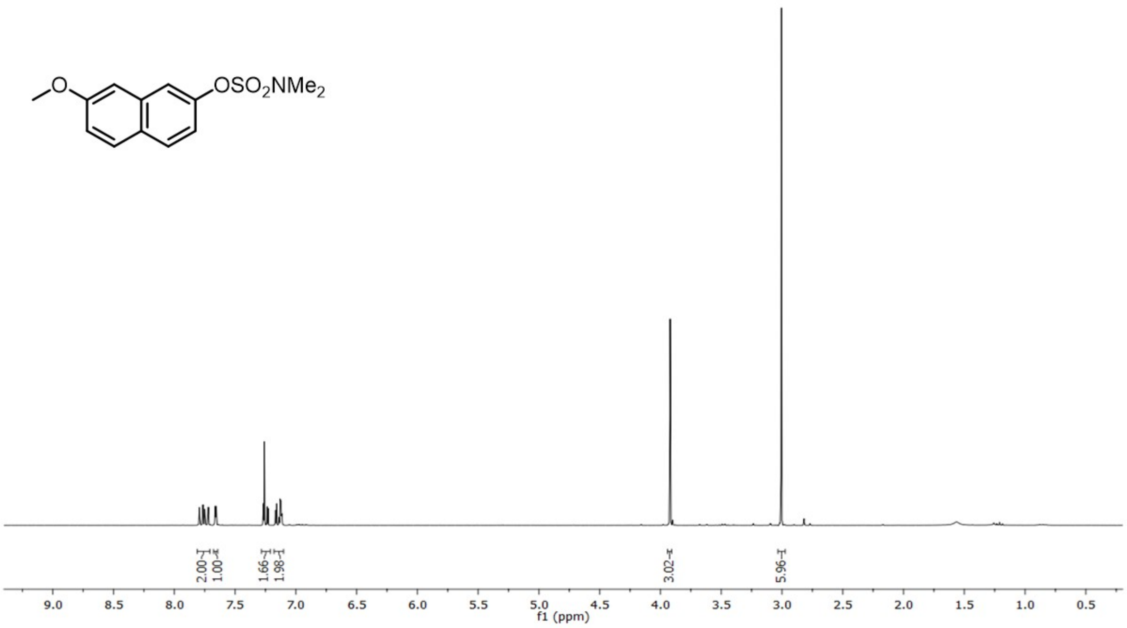

$^{13}C\{^1H\}$ NMR spectrum of 7-methoxynaphthalen-2-yl-dimethylsulfamate, 2g, (75 MHz, $CDCl_3$, 298 K)

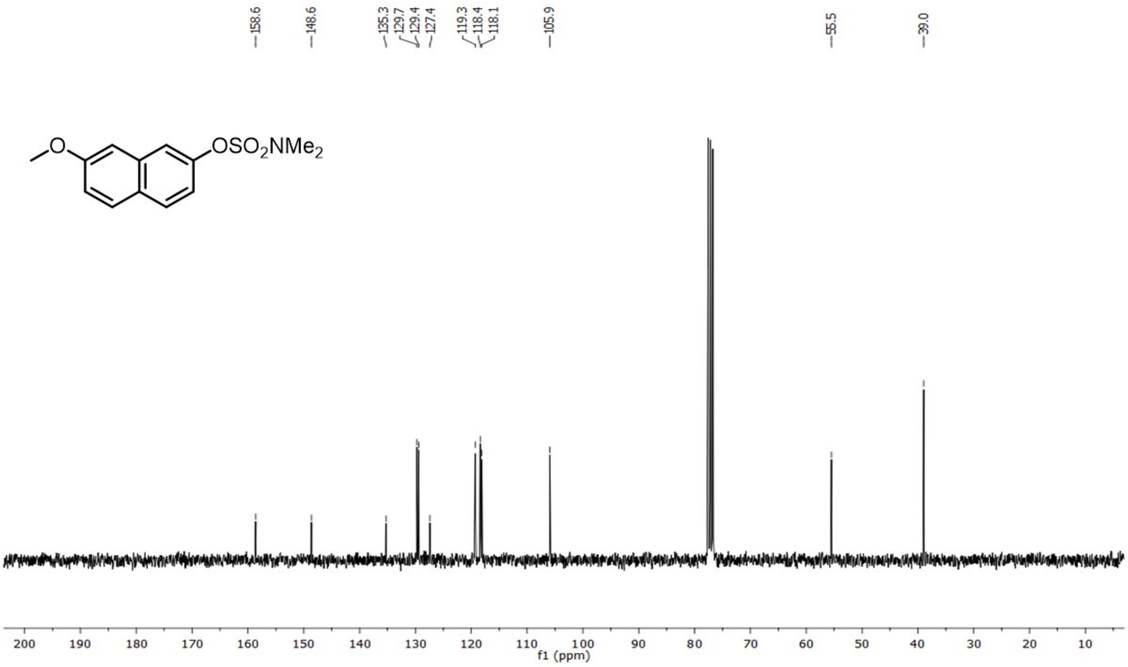

^{1}H NMR spectrum of *N*-phenylnaphthalen-1-amine, 4a, (300 MHz, $CDCl_3$, 298 K)

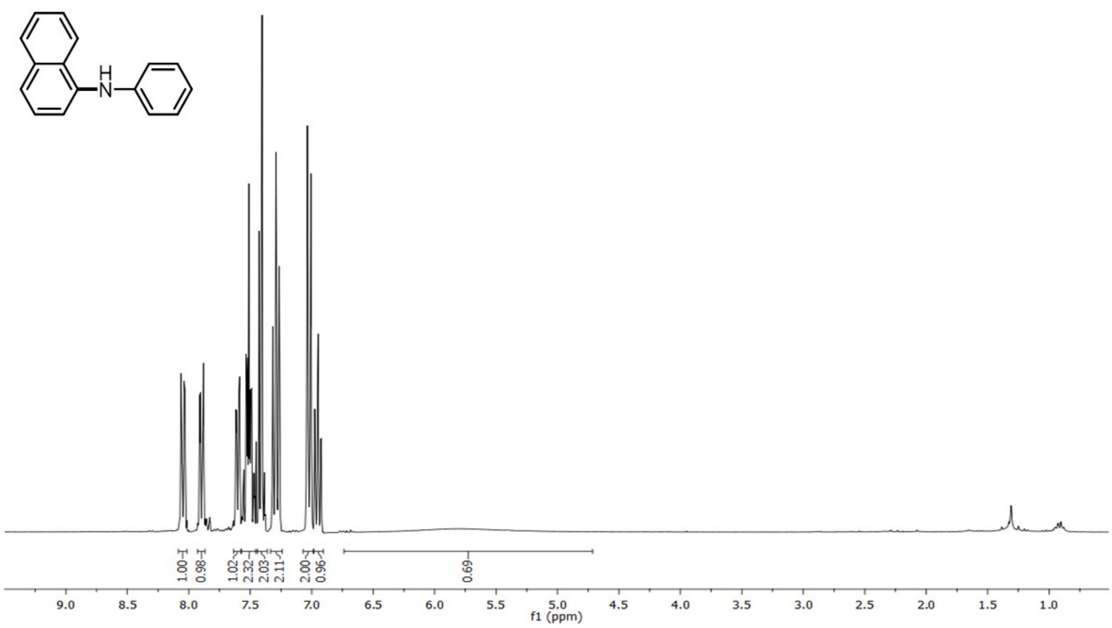

^{1}H NMR spectrum of 4-(naphthalen-1-ylamino)phenol, 4b, (300 MHz, $CDCl_3$, 298 K)

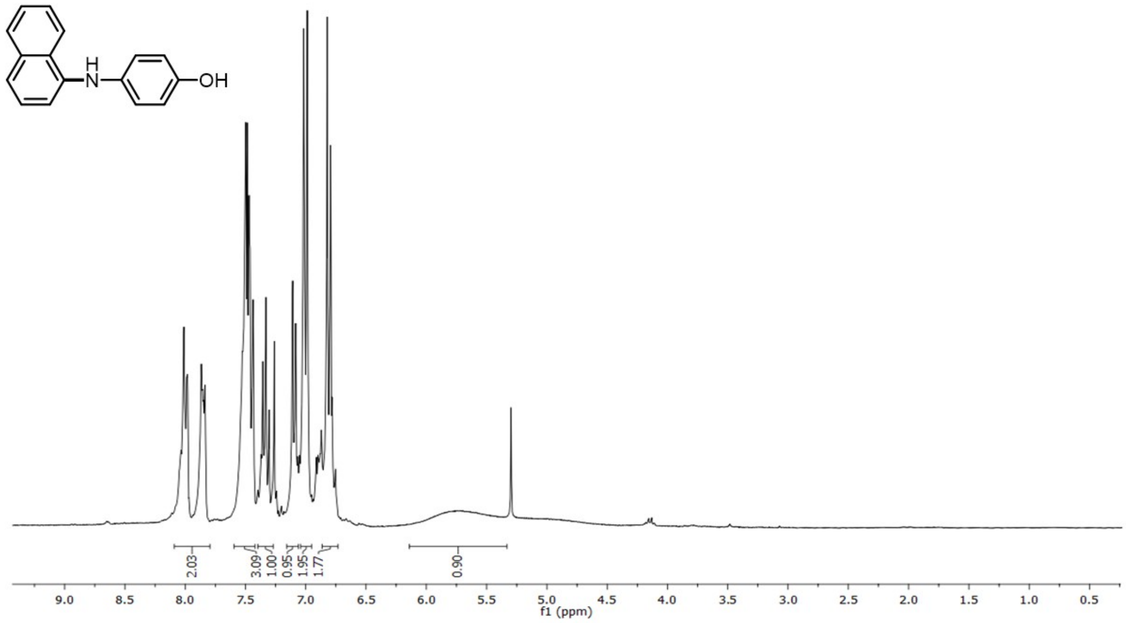

^{13}C{^{1}H} NMR spectrum of 4-(naphthalen-1-ylamino)phenol, 4b, (75 MHz, $CDCl_3$, 298 K)

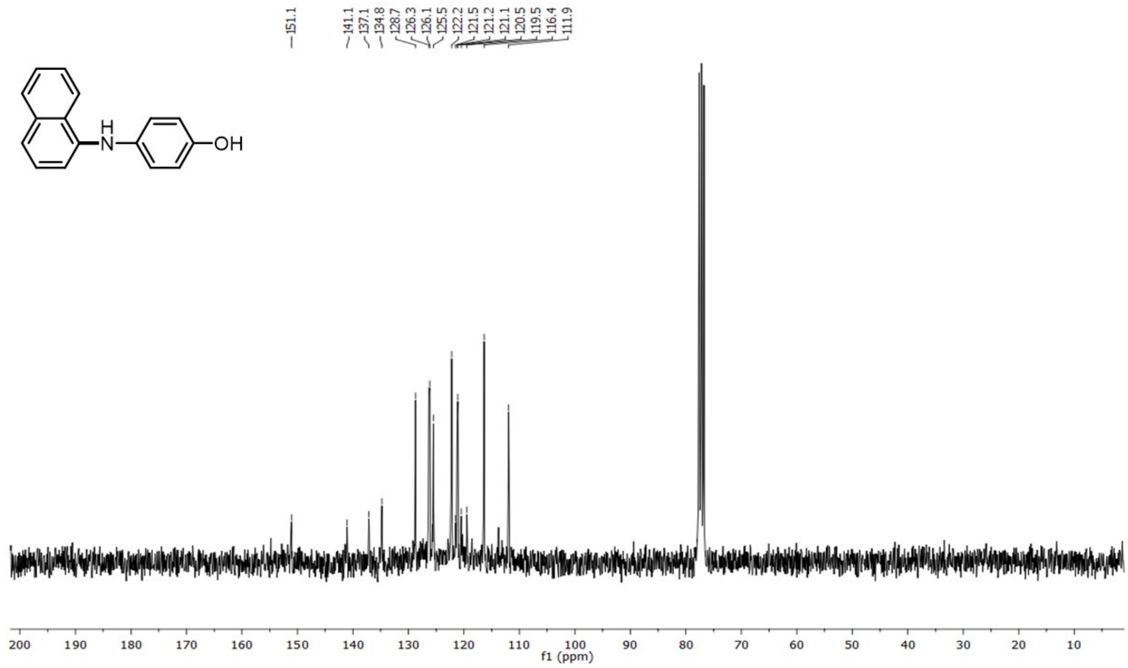

^{1}H NMR spectrum of *N*-(4-Methoxyphenyl)naphthalen-1-amine, 4c, (300 MHz, $CDCl_3$, 298 K)

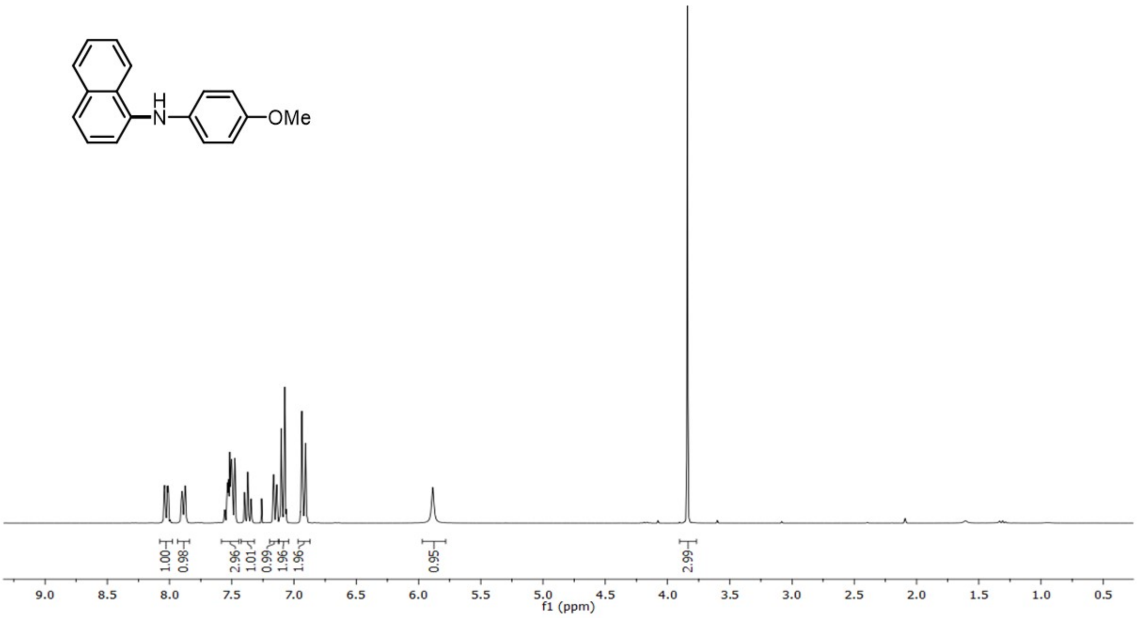

^{1}H NMR spectrum of *N*-(4-methylphenyl)naphthalen-1-amine, 4d, (75 MHz, $CDCl_3$, 298 K)

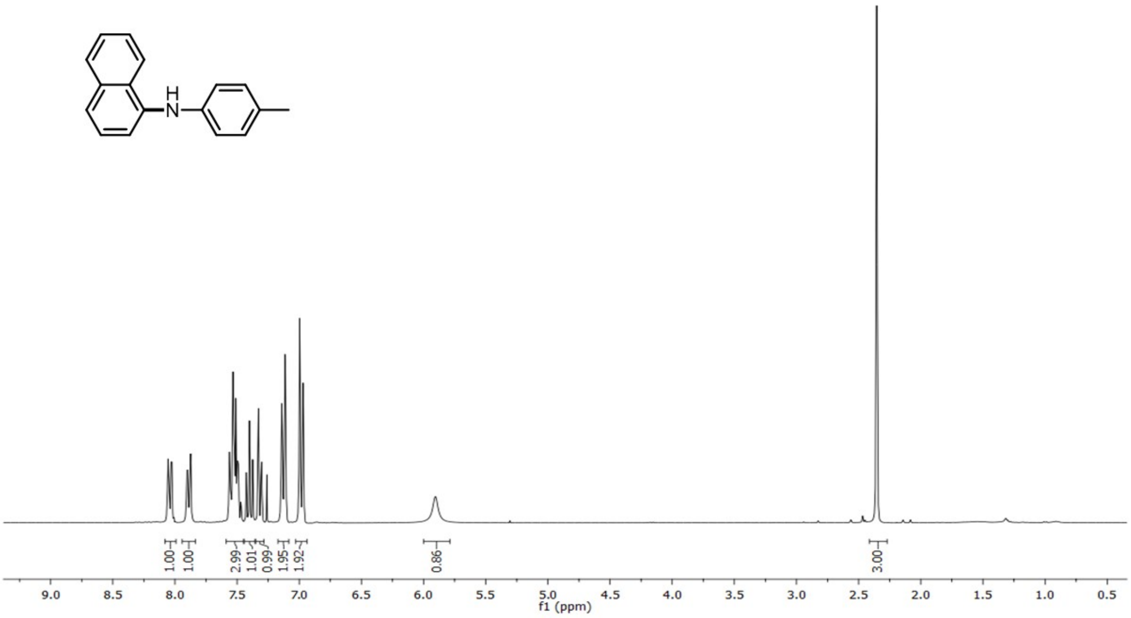

^{1}H NMR spectrum of *N*-(2,4,6-Trimethylphenyl)naphthalen-1-amine, 4e, (300 MHz, $CDCl_3$, 298 K)**.**

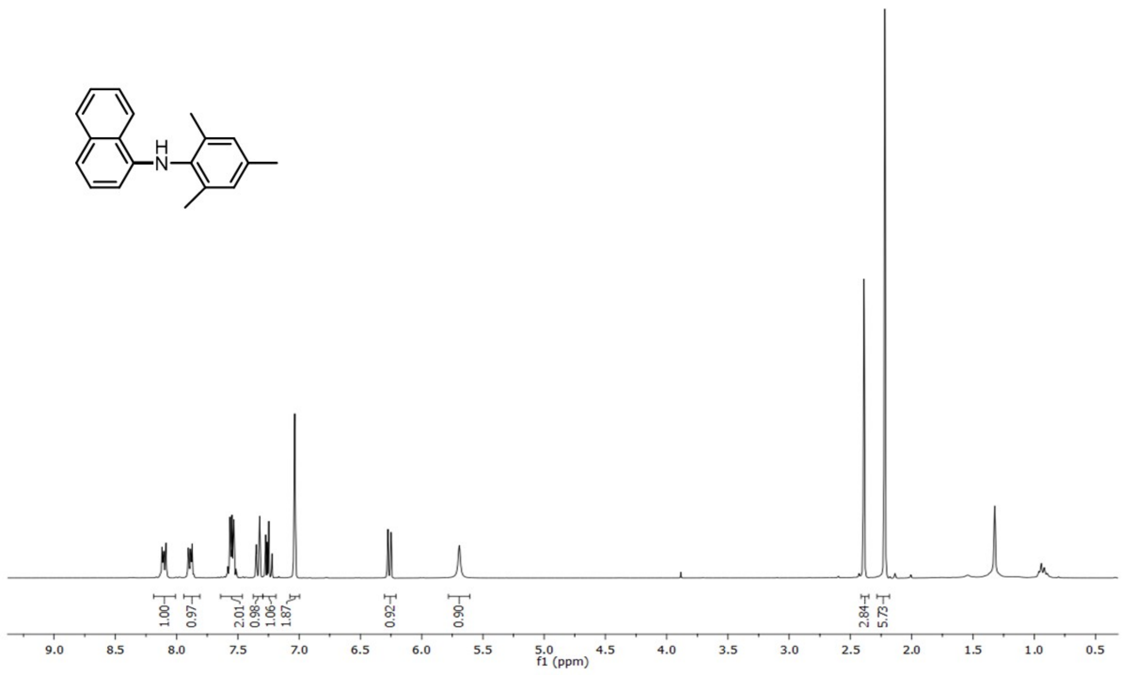

^{1}H NMR spectrum of *N*-(2,6-diisopropylphenyl)naphthalen-1-amine, 4f, (300 MHz, $CDCl_3$, 298 K)

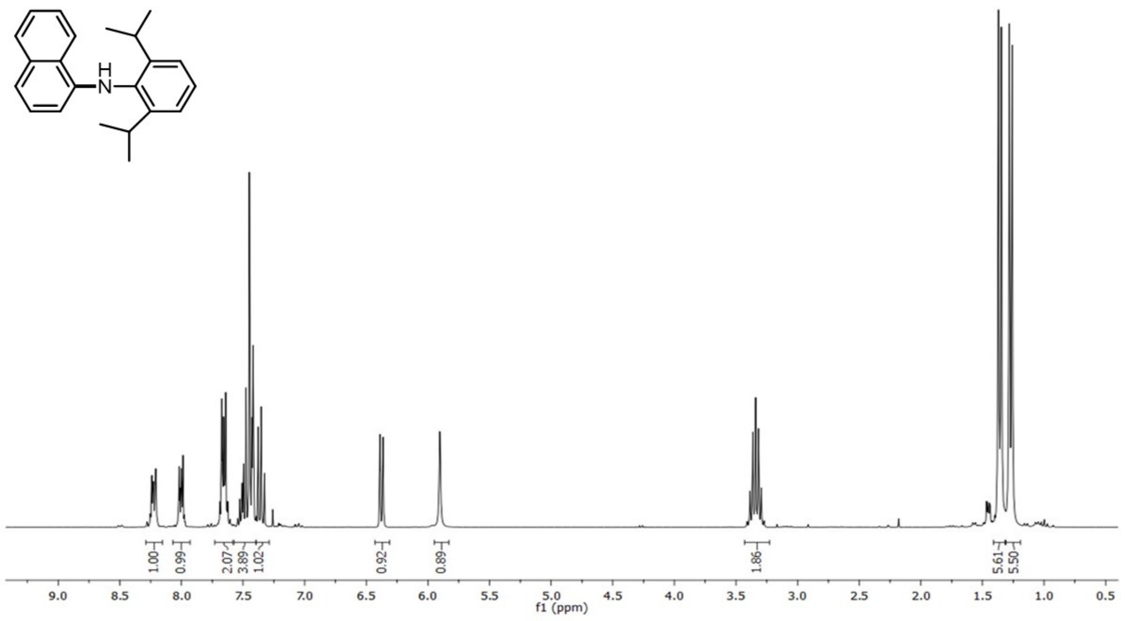

^{1}H NMR spectrum of *N*-([1,1′-biphenyl]-2-yl)naphthalen-1-amine, 4g, (300 MHz, $CDCl_3$, 298 K)

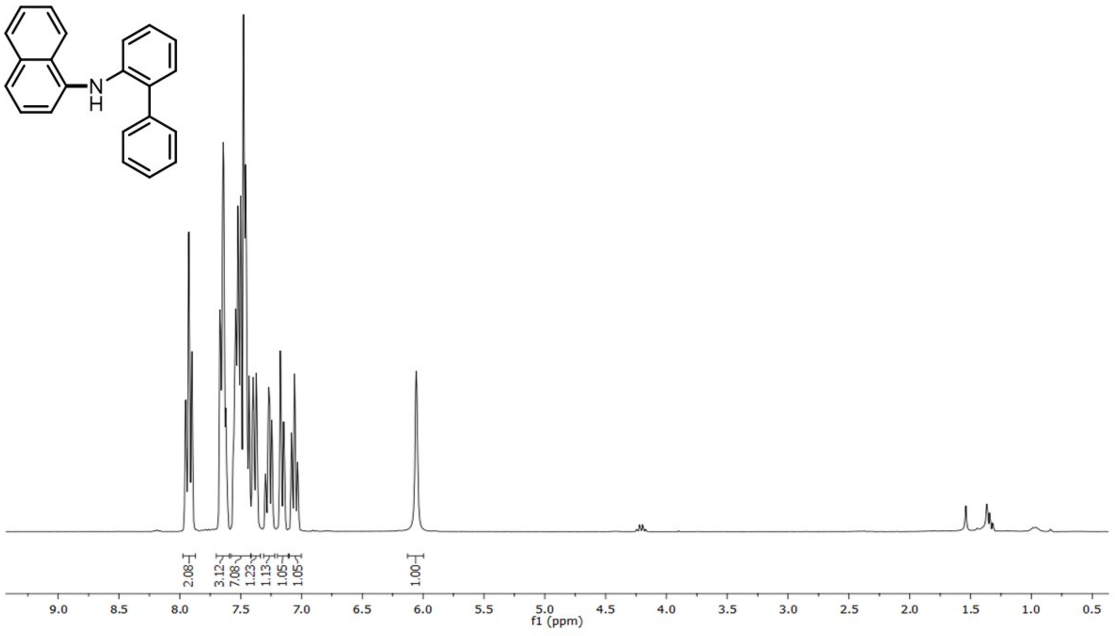

^{1}H NMR spectrum of ethyl 4-(naphthalen-1-ylamino)benzoate, 4h, (300 MHz, $CDCl_3$, 298 K)

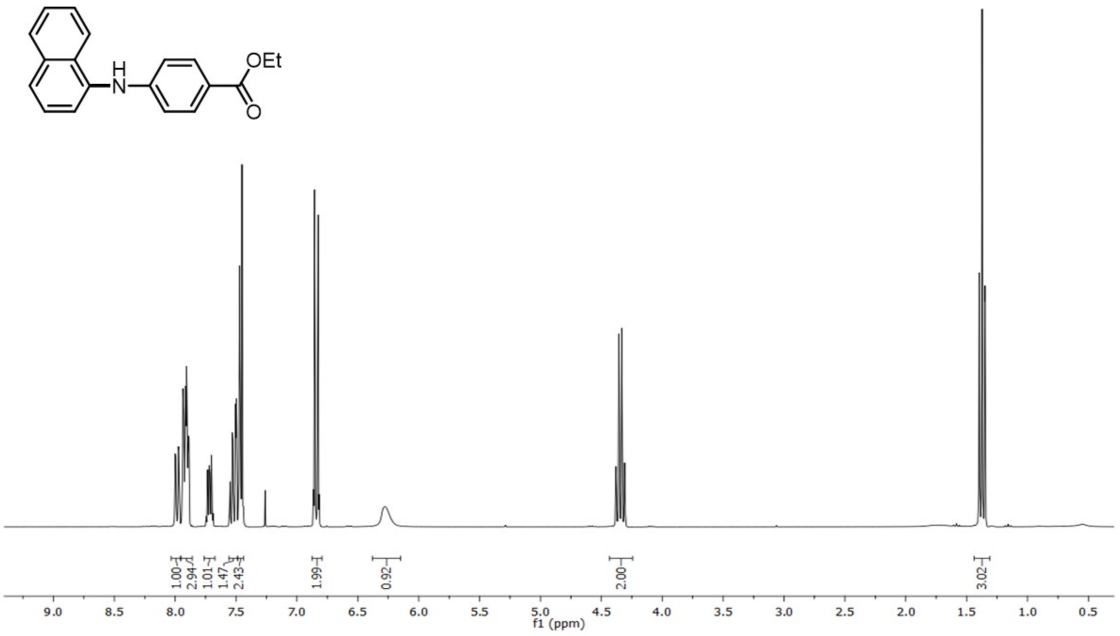

^{1}H NMR spectrum of 4-(Naphthalen-1ylamino)benzonitrile 4i, (300 MHz, $CDCl_3$, 298 K)

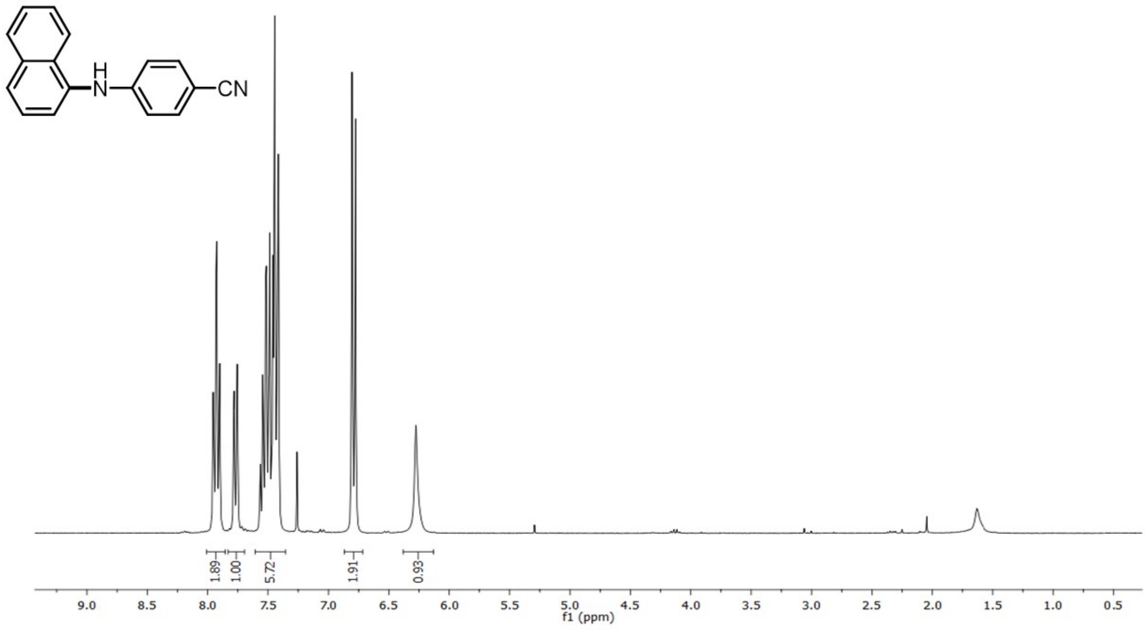

^{13}C{^{1}H} NMR spectrum of 4-(Naphthalen-1ylamino)benzonitrile, 4i, (75 MHz, $CDCl_3$, 298 K)

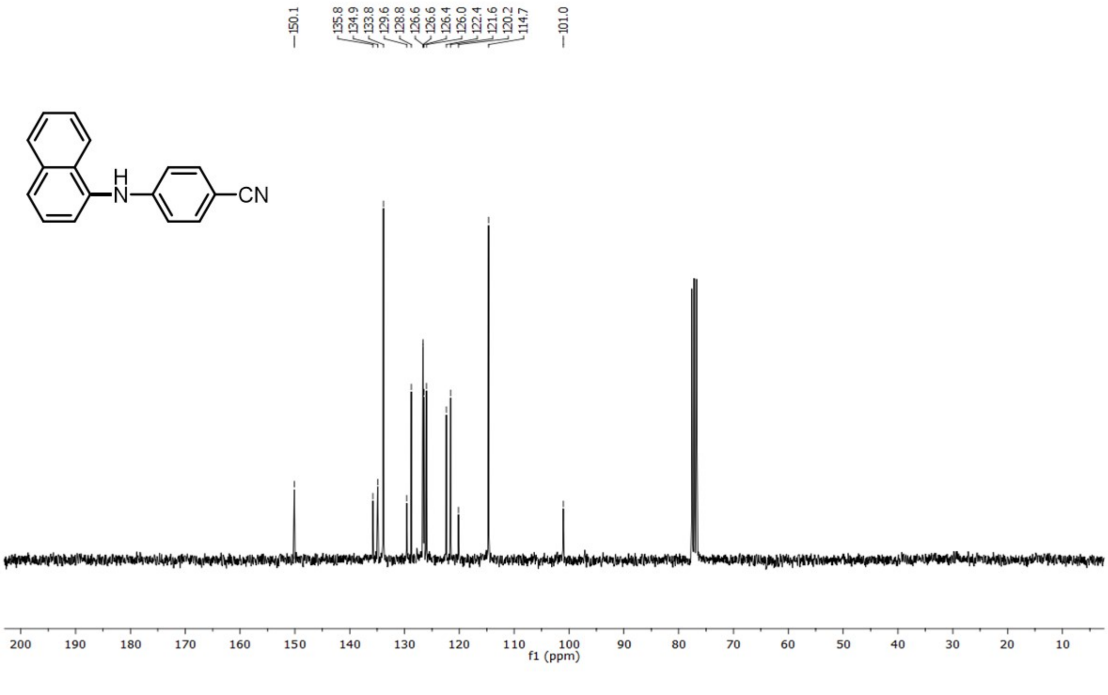

^{1}H NMR spectrum of *N*-(4-chlorophenyl)naphthalen-1-amine, 4j, (300 MHz, $CDCl_3$, 298 K)

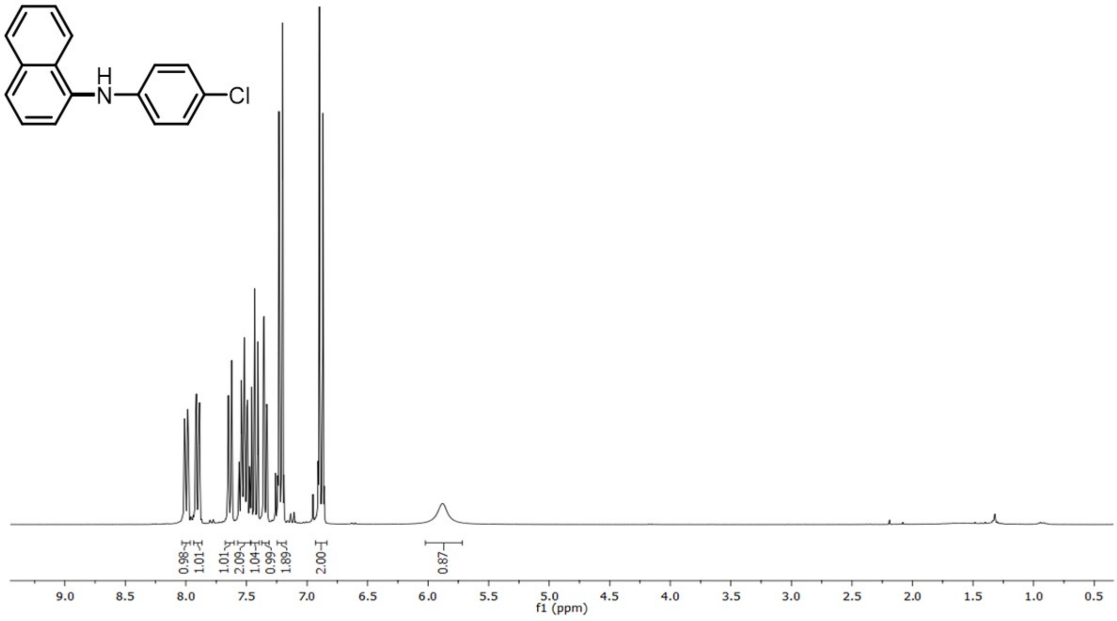

^{1}H NMR spectrum of *N*-phenylnaphthalen-2-amine, 4k, (300 MHz, $CDCl_3$, 298 K)

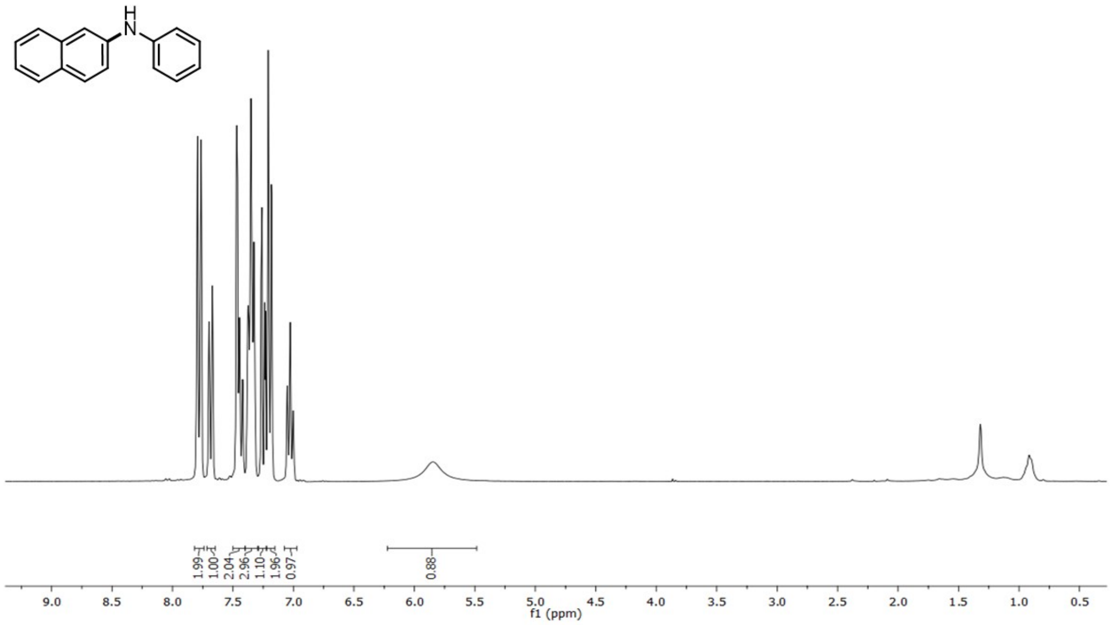

^{1}H NMR spectrum of 6-(phenylamino)-2-naphthonitrile, 4l, (300 MHz, $CDCl_3$, 298 K)

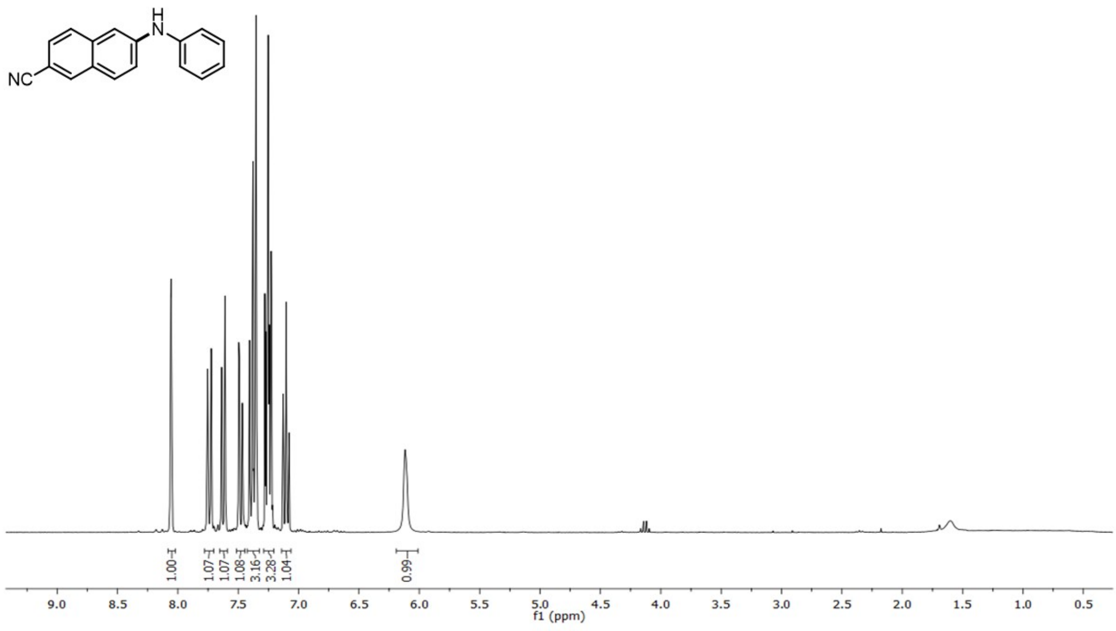

^{13}C{^{1}H} NMR spectrum of 6-(phenylamino)-2-naphthonitrile, 4l, (75 MHz, $CDCl_3$, 298 K)

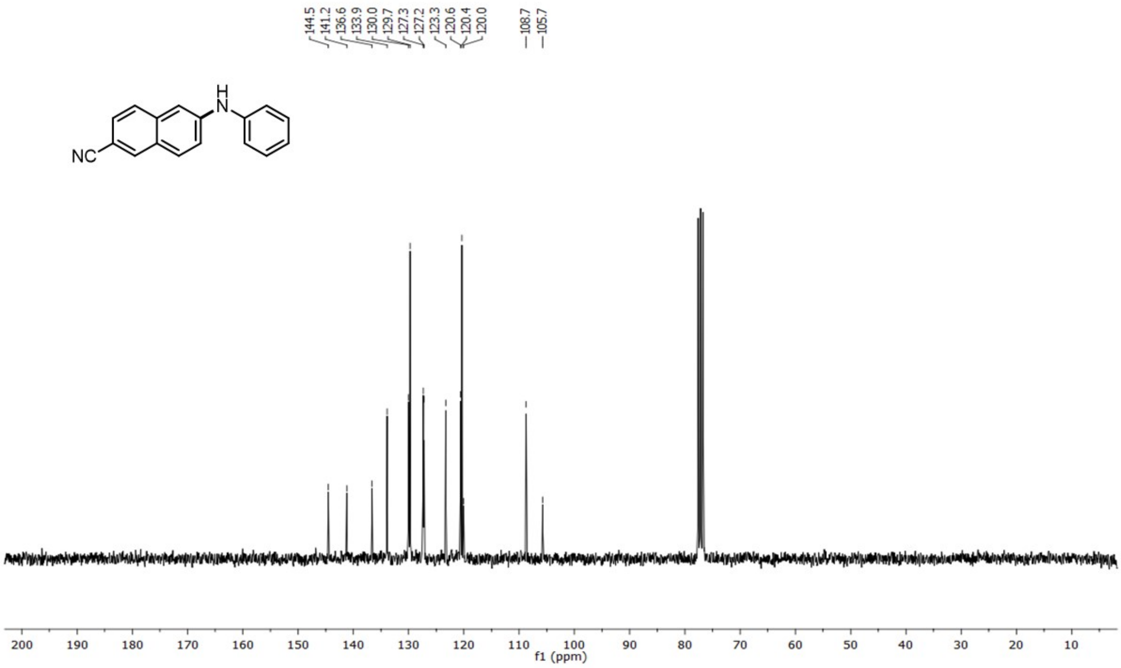

^{1}H NMR spectrum of *N*-[3,5-Bis(trifluoromethyl)phenyl]naphthalen-2-amine, 4, (300 MHz, $CDCl_3$, 298 K)**)**

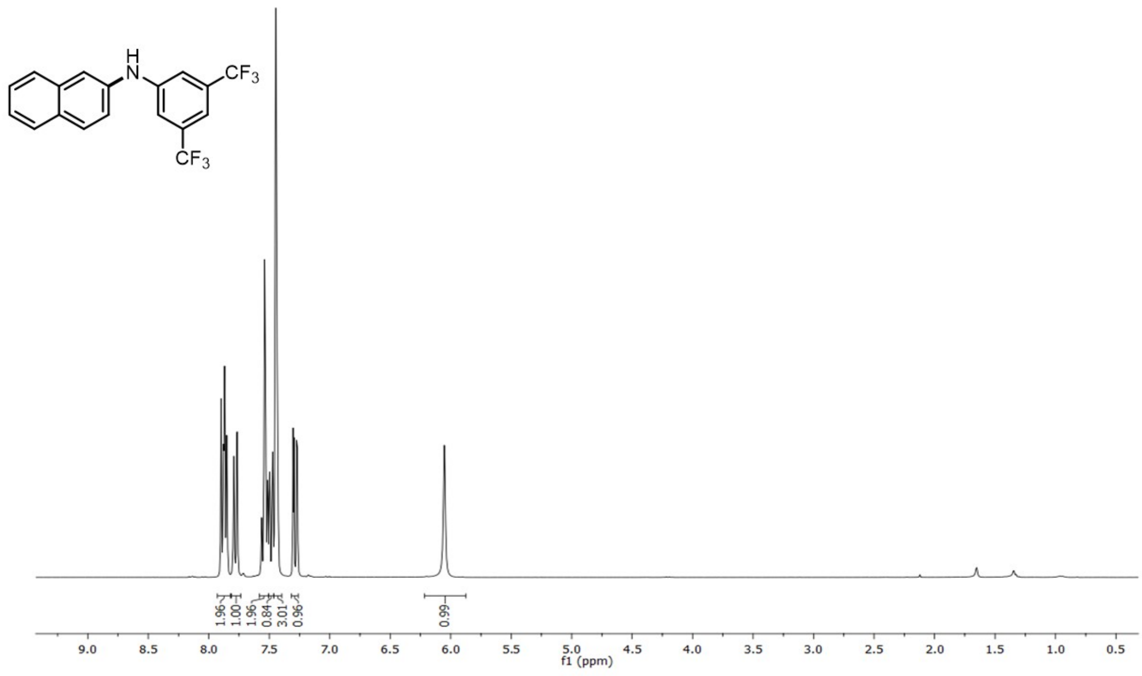

^{13}C{^{1}H} NMR spectrum of *N*-[3,5-Bis(trifluoromethyl)phenyl]naphthalen-2-amine, 4m, (75 MHz, $CDCl_3$, 298 K)

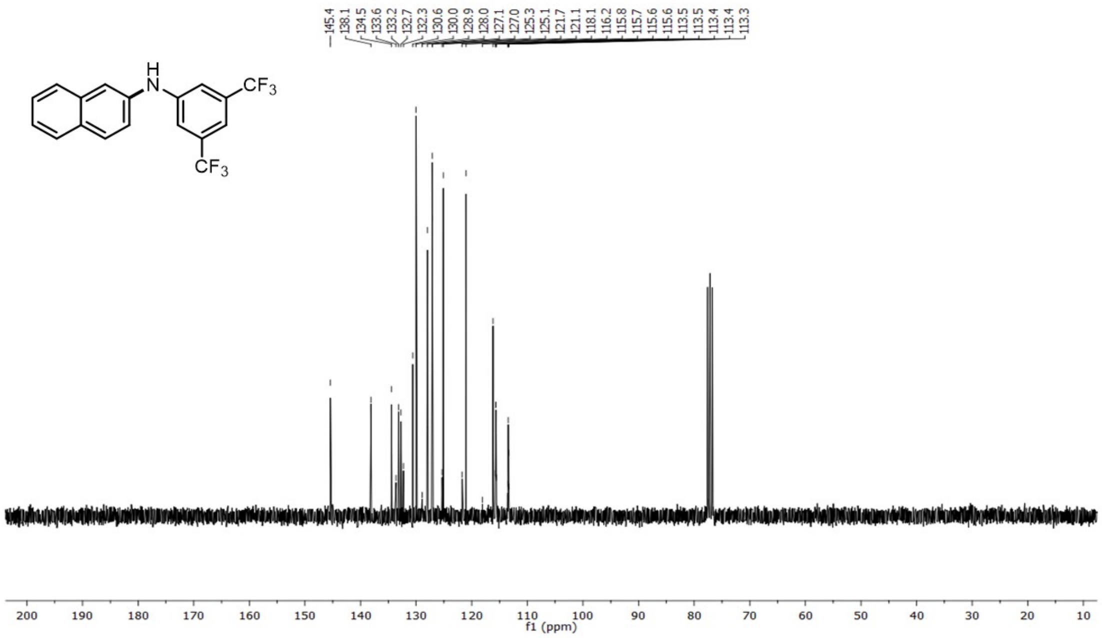

^{1}H NMR spectrum of *N*-phenylquinolin-6-amine, 4n, (300 MHz, $CDCl_3$, 298 K)

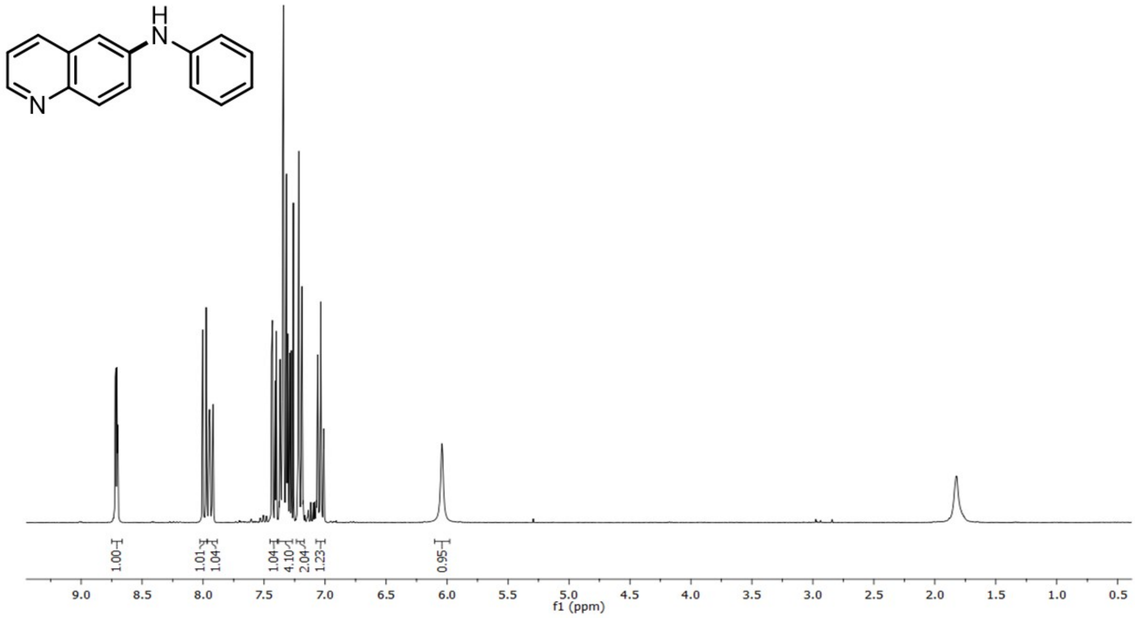

^{1}H NMR spectrum of 4-(phenylamino)benzonitrile, 4o, (300 MHz, $CDCl_3$, 298 K)

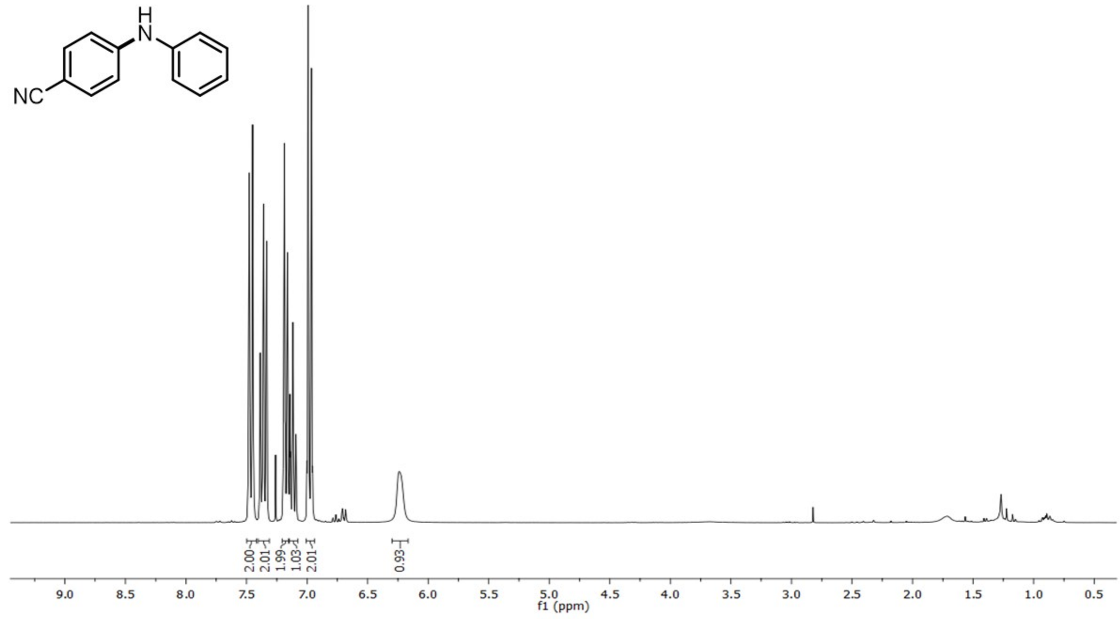

^{1}H NMR spectrum of diphenylamine, 4p, (300 MHz, $CDCl_3$, 298 K)

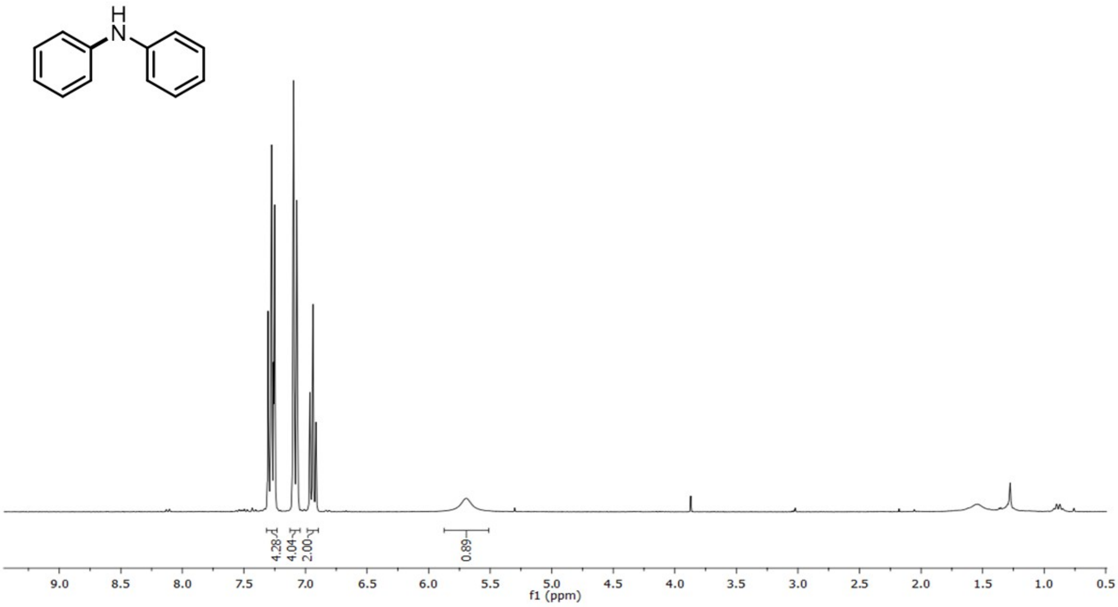

^{1}H NMR spectrum of 6-morpholino-2-naphthonitrile, 5a, (300 MHz, $CDCl_3$, 298 K)

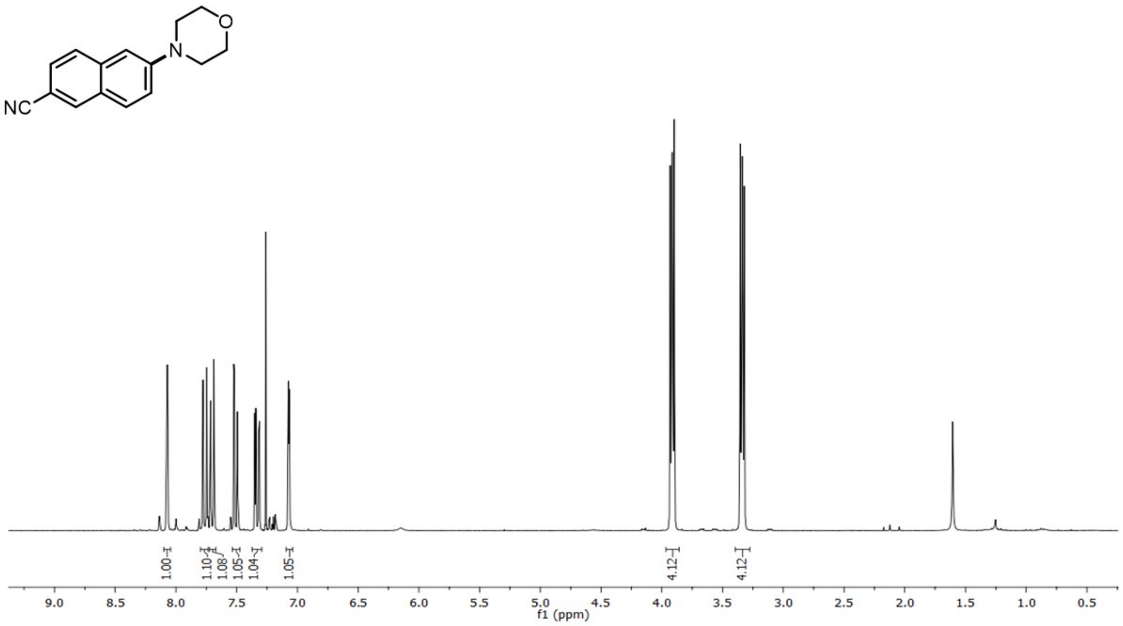

^{13}C{^{1}H} NMR spectrum of 6-morpholino-2-naphthonitrile, 5a (75 MHz, $CDCl_3$, 298 K)

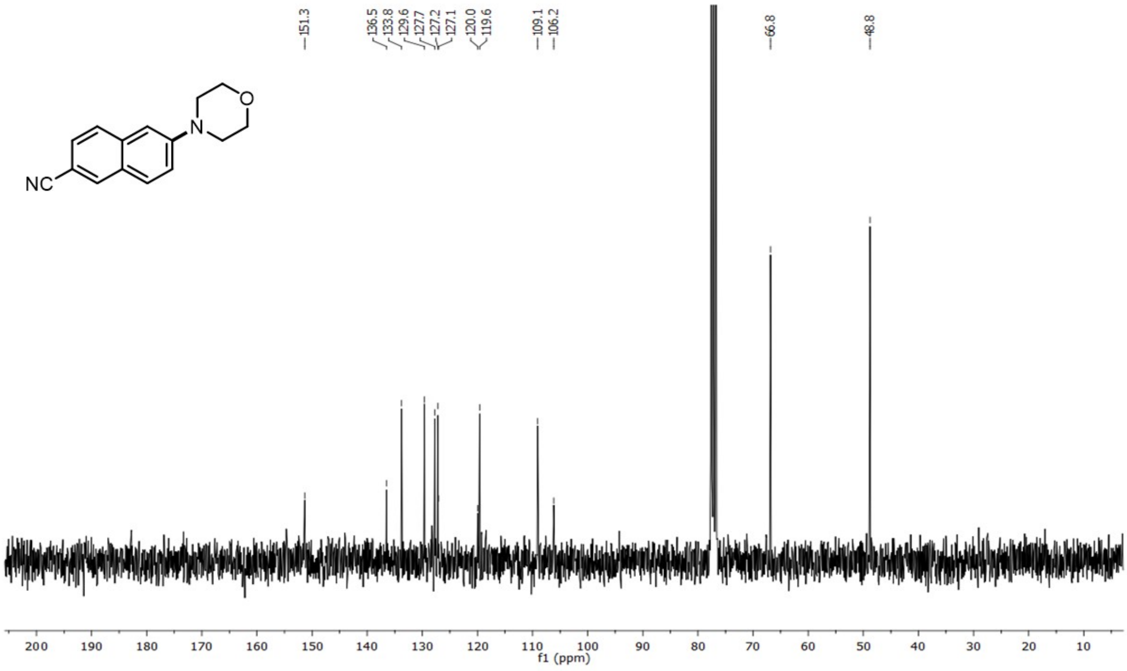

^{1}H NMR spectrum of 6-(Piperidin-1-yl)-2-naphthonitrile, 5b, (300 MHz, $CDCl_3$, 298 K)

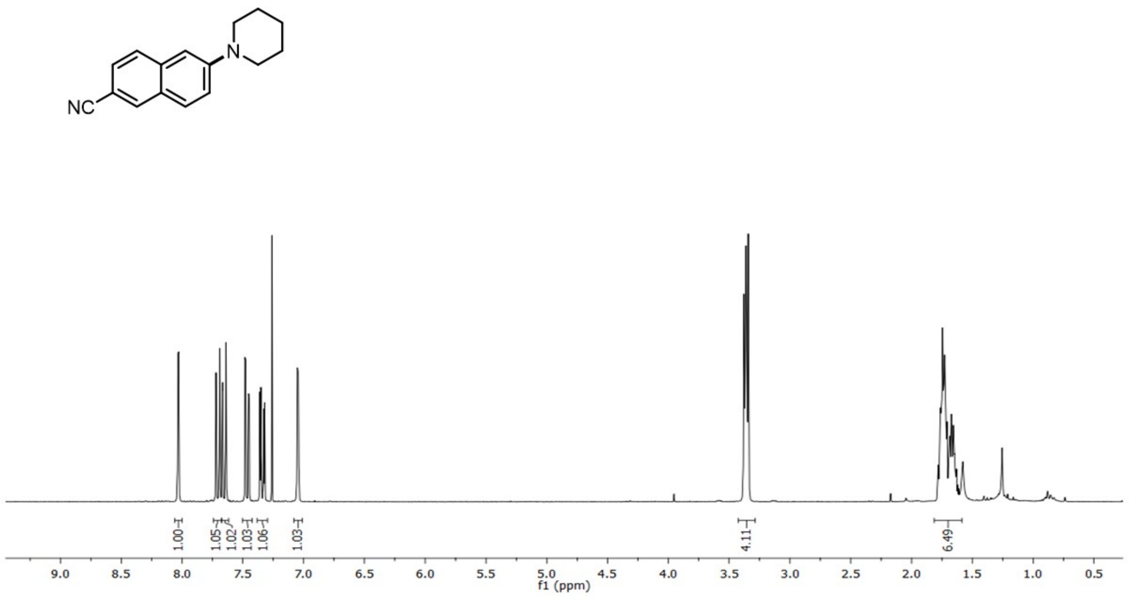

$^{13}C\{^1H\}$ NMR spectrum of 6-(Piperidin-1-yl)-2-naphthonitrile, 5b, (75 MHz, $CDCl_3$, 298 K)

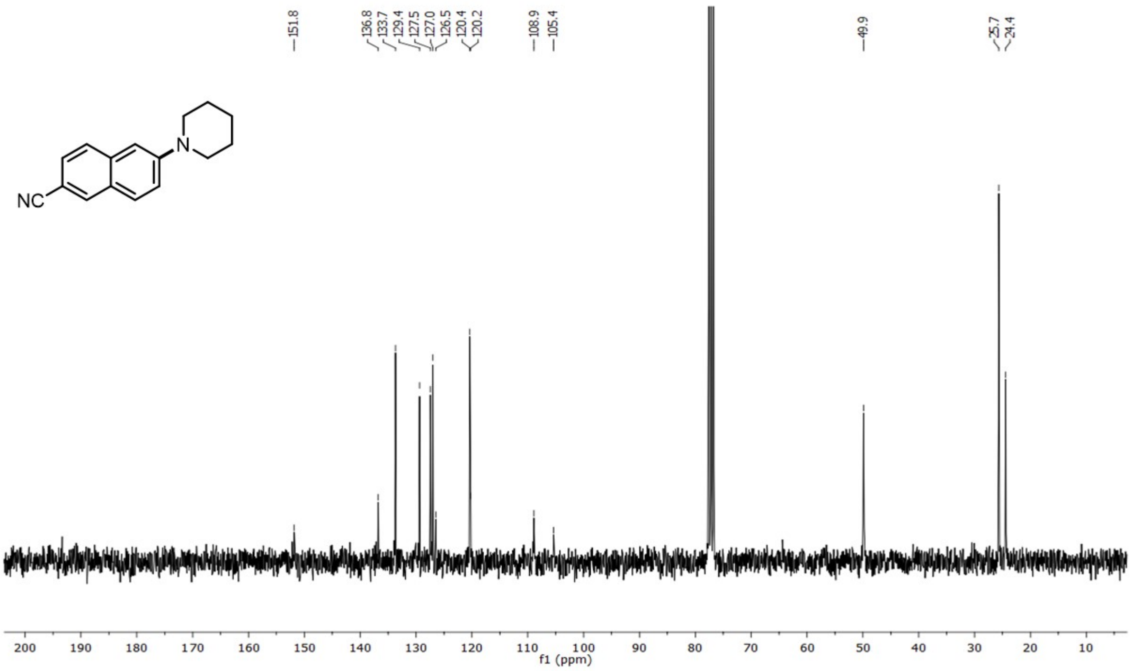

^{1}H NMR spectrum of 1-(7-Methoxynaphthalen-2-yl)pyrrolidine, 5c, (300 MHz, $CDCl_3$, 298 K)

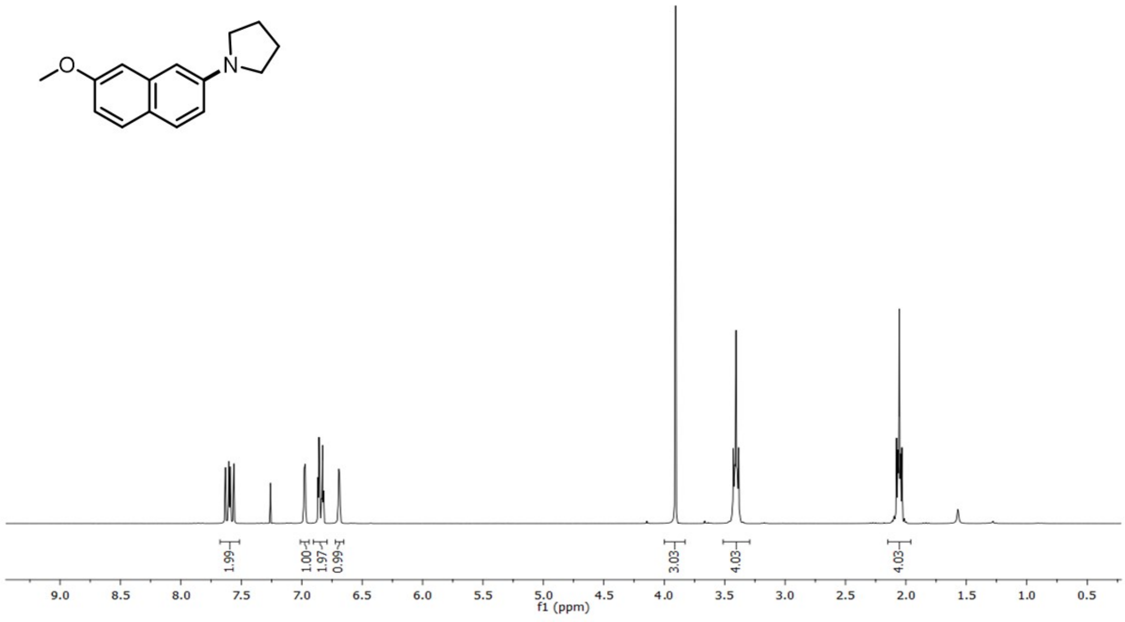

$^{13}C\{^{1}H\}$ NMR spectrum of 1-(7-Methoxynaphthalen-2-yl)pyrrolidine, 5c, (75 MHz, $CDCl_3$, 298 K)

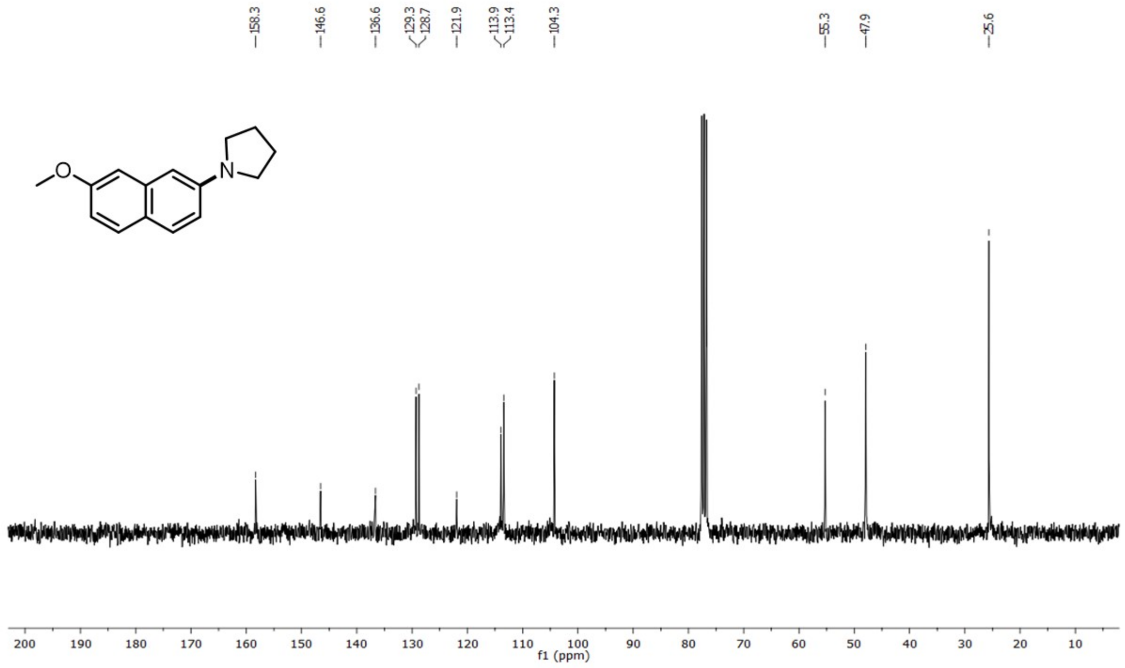

^{1}H NMR spectrum of 1-(naphthalen-2-yl)indoline, 5d, (300 MHz, $CDCl_3$, 298 K)**.**

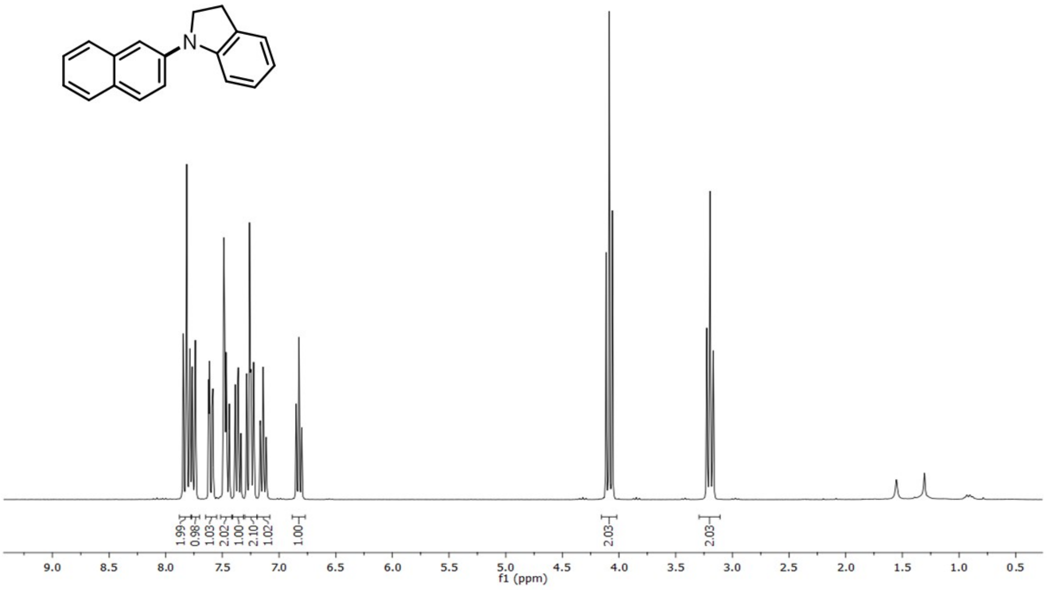

^{1}H NMR spectrum of 1-(7-methoxynaphthalen-2-yl)-2-methylpiperidine, 5e, (300 MHz, $CDCl_3$, 298 K)

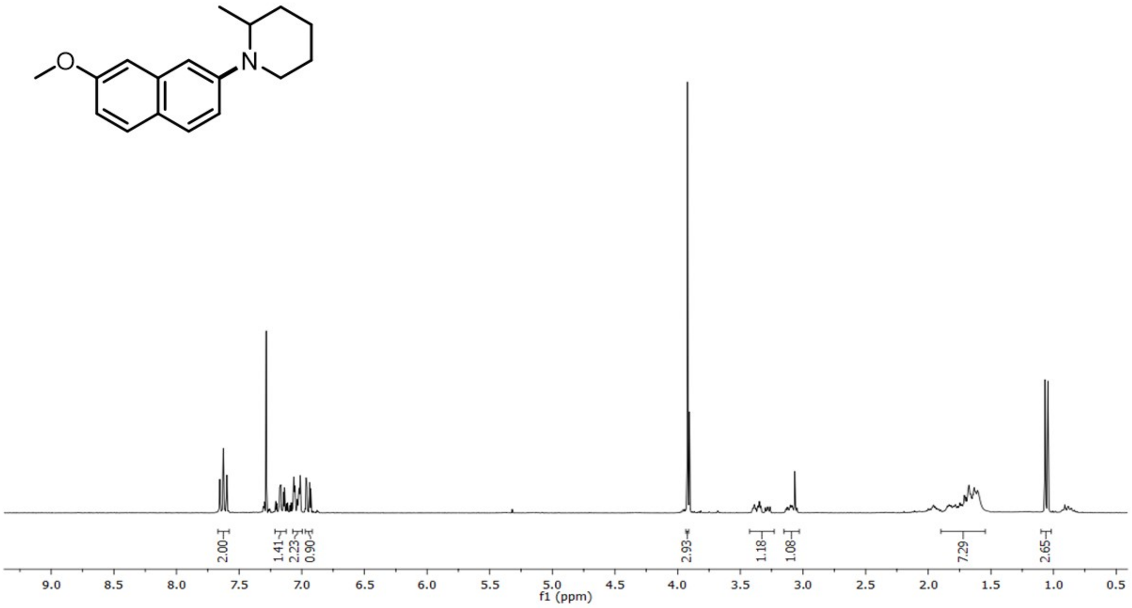

^{13}C{^{1}H} NMR spectrum of1-(7-methoxynaphthalen-2-yl)-2-methylpiperidine, 5e, (75 MHz, $CDCl_3$, 298 K)**.**

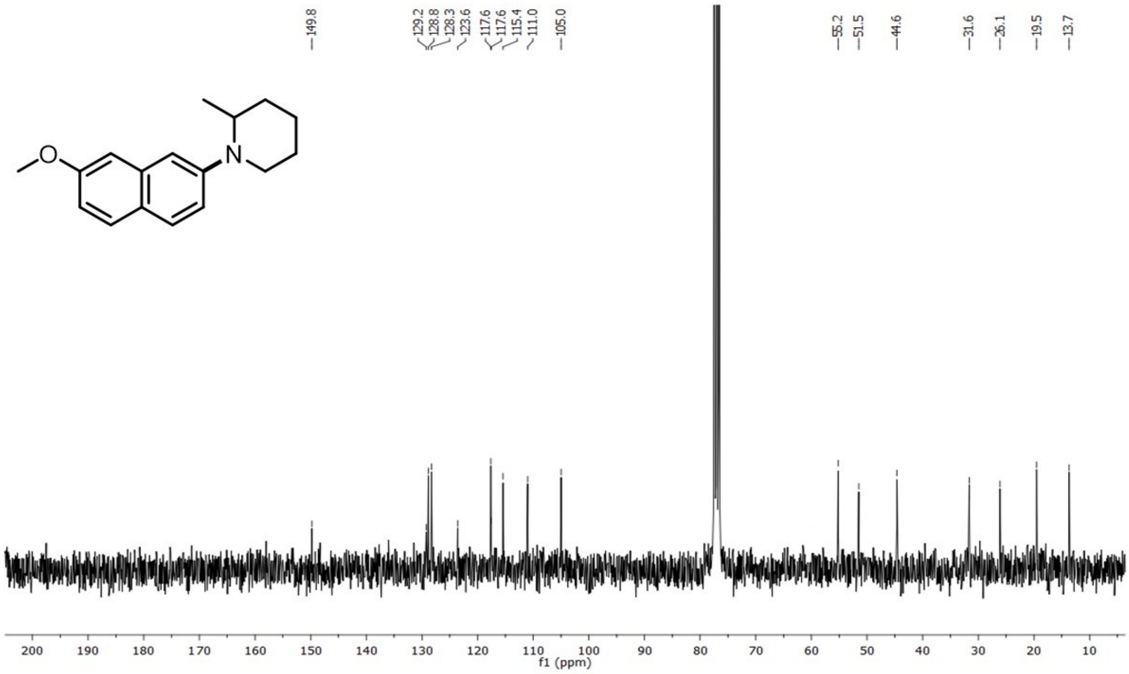

^{1}H NMR spectrum of 4-(Naphthalen-1-yl)morpholine, 5f, (300 MHz, $CDCl_3$, 298 K)

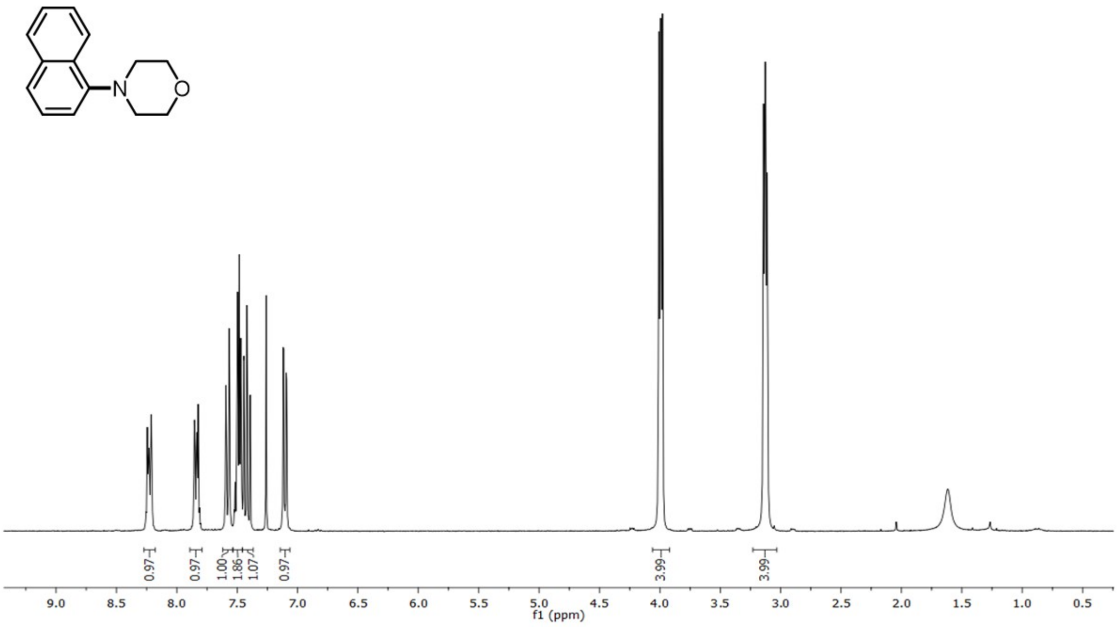

^{1}H NMR spectrum of *N*-Methyl-*N*-phenylnaphthalen-1-amine, 5g, (300 MHz, $CDCl_3$, 298 K)**.**

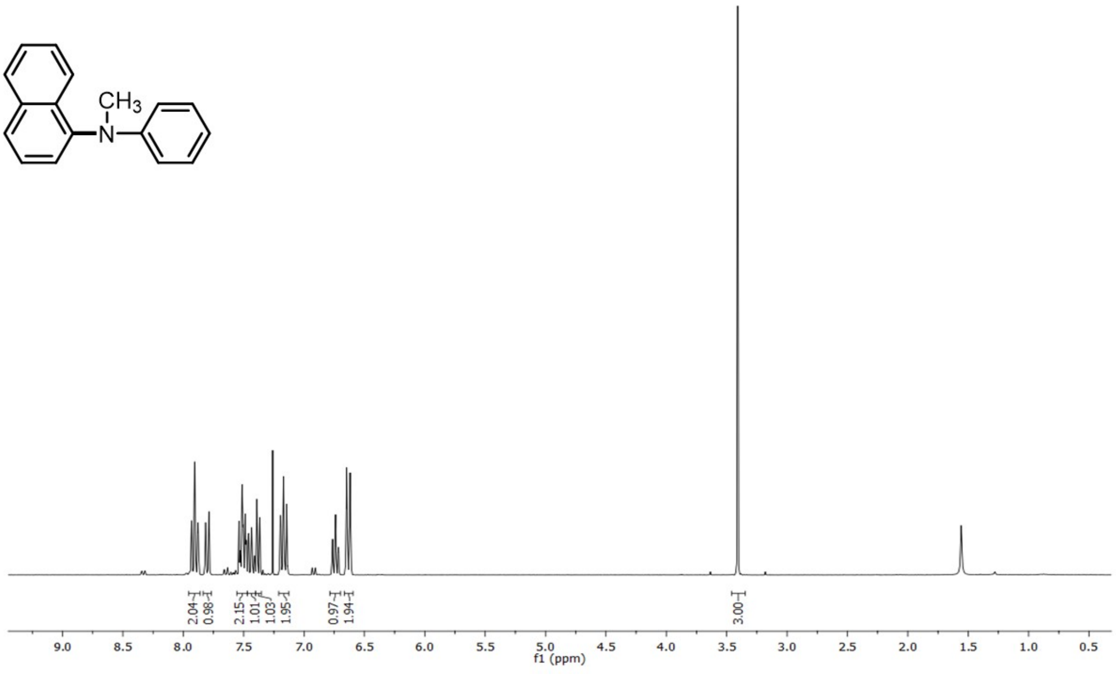

^{1}H NMR spectrum of *N*,*N*-Dibenzylnaphthalen-2-amine, 5h, (300 MHz, $CDCl_3$, 298 K)

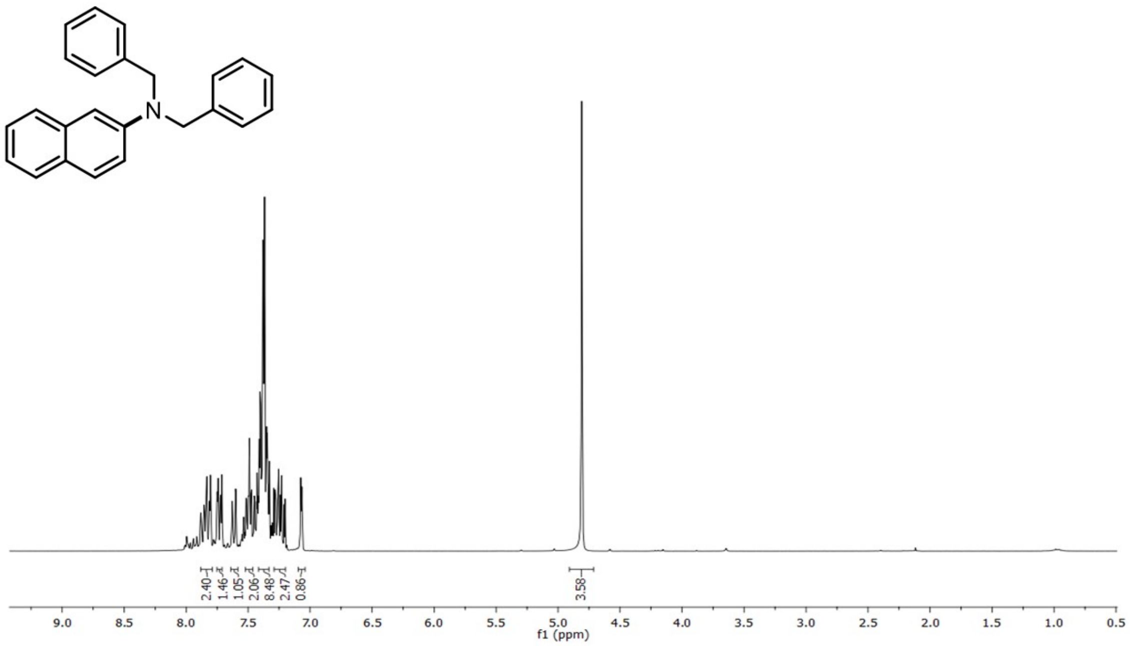

^{1}H NMR spectrum of *N*-benzylnaphthalen-2-amine, 5i, (300 MHz, $CDCl_3$, 298 K)

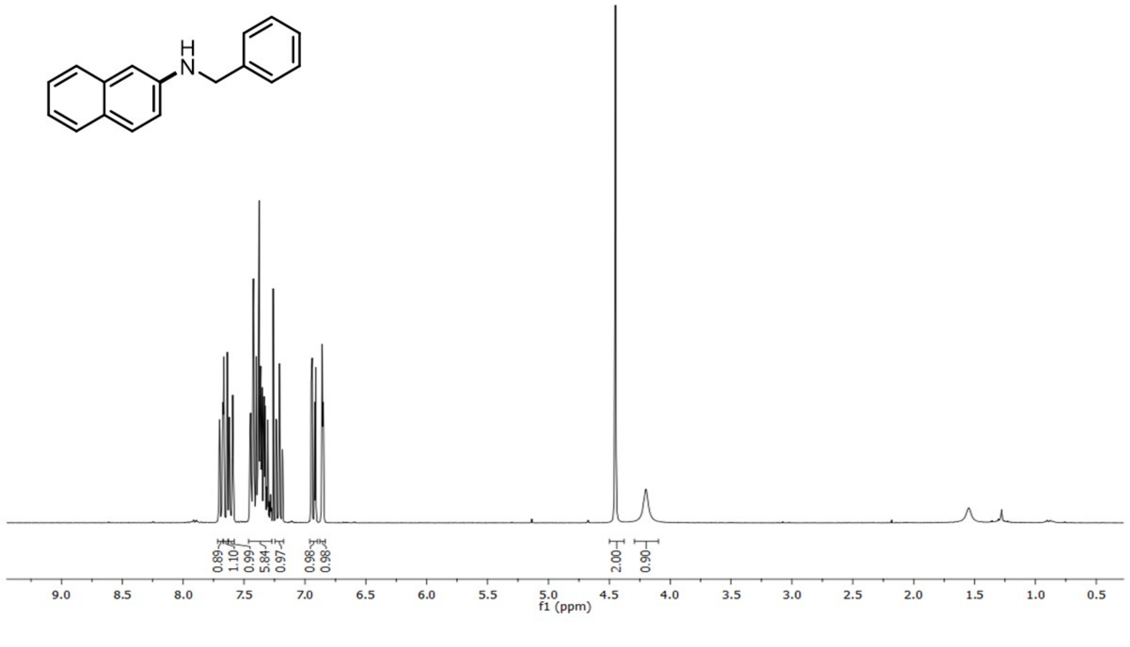

^{1}H NMR spectrum of 4-(hexylamino)benzonitrile, 5j, (300 MHz, $CDCl_3$, 298 K)

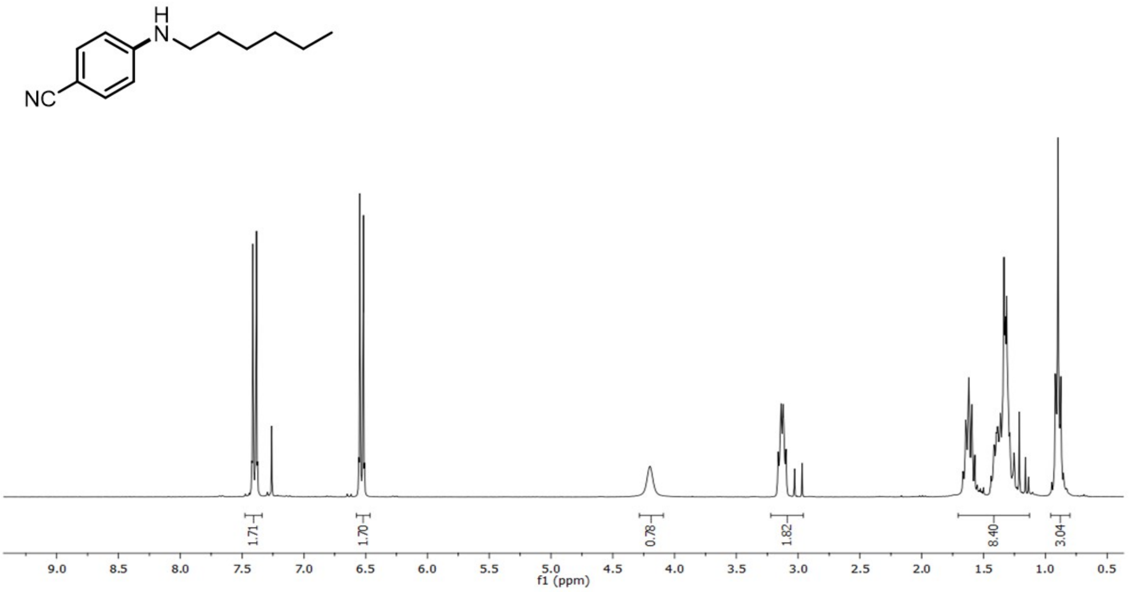

^{1}H NMR spectrum of 4-(butylamino)benzonitrile, 5k, (300 MHz, $CDCl_3$, 298 K)

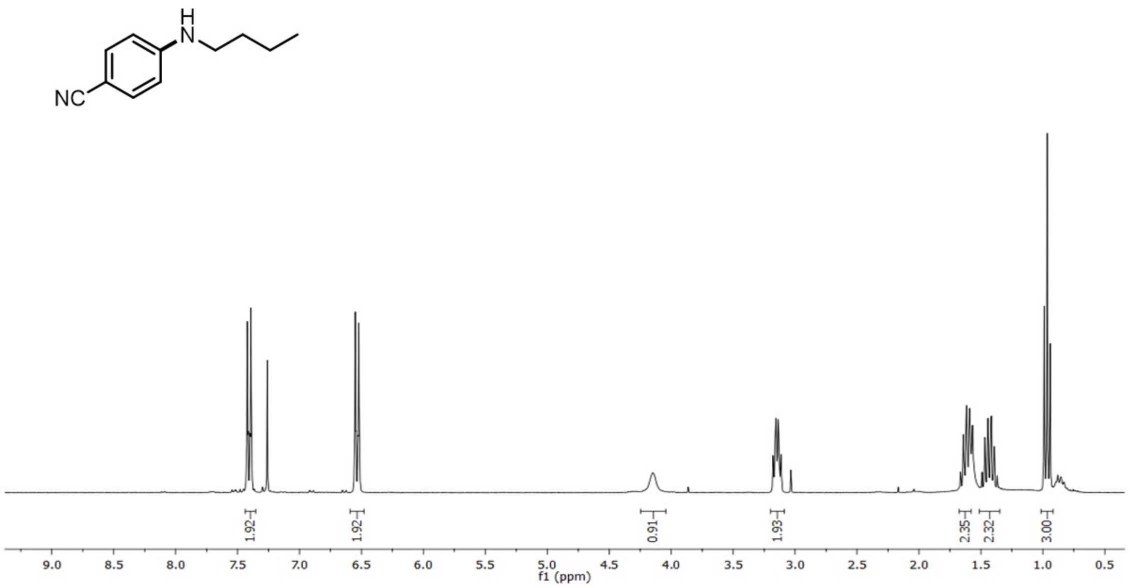

^{1}H NMR spectrum of *N*-hexylnaphthalen-2-amine, 5l, (300 MHz, $CDCl_3$, 298 K)**.**

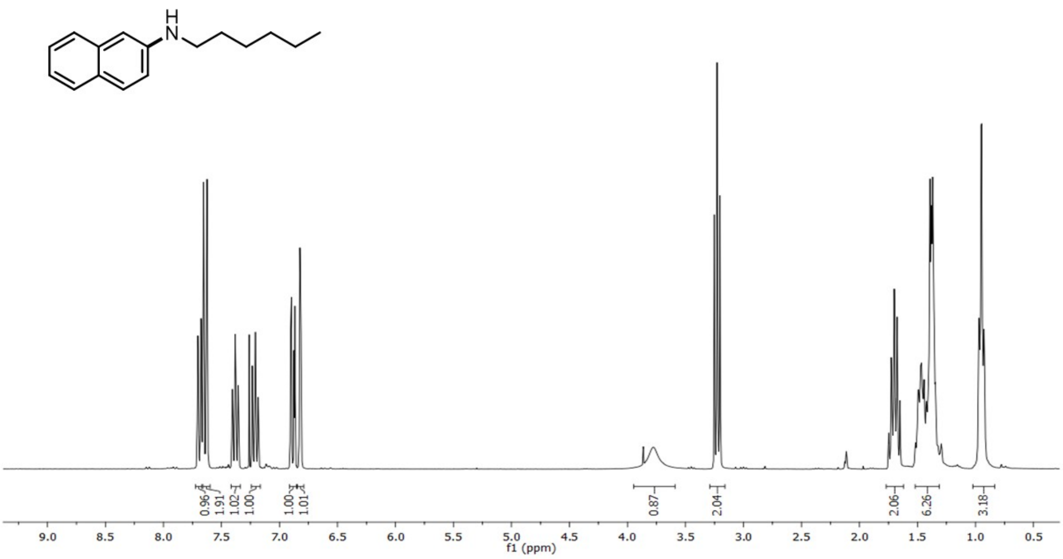

^{1}H NMR spectrum of *N*-butylnaphthalen-2-amine, 5m, (300 MHz, $CDCl_3$, 298 K)

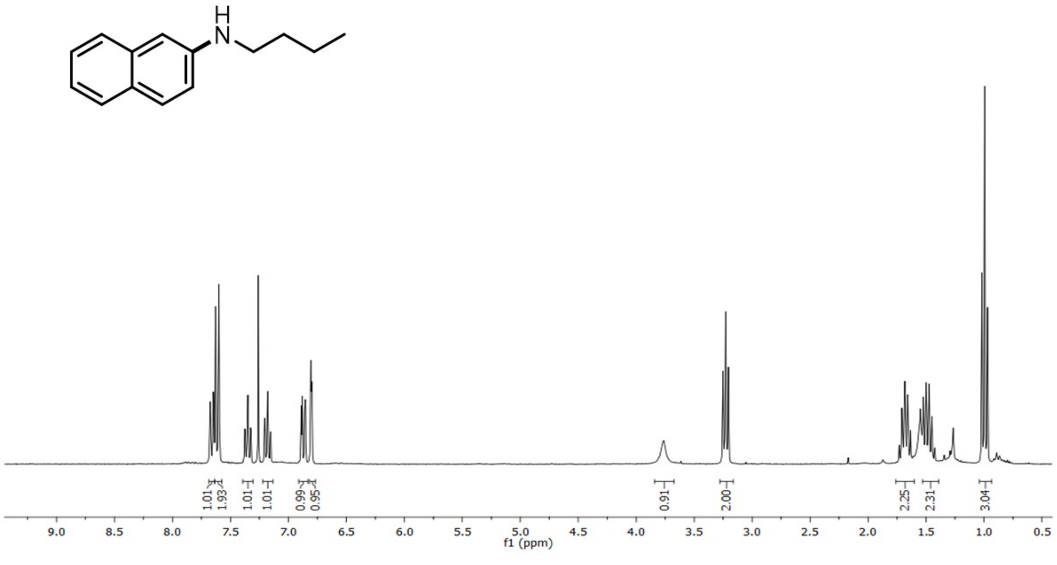

^{1}H NMR spectrum of *N*-(naphthalen-2-yl)pyridyl-3-amine, 6a, (300 MHz, $CDCl_3$, 298 K)**.**

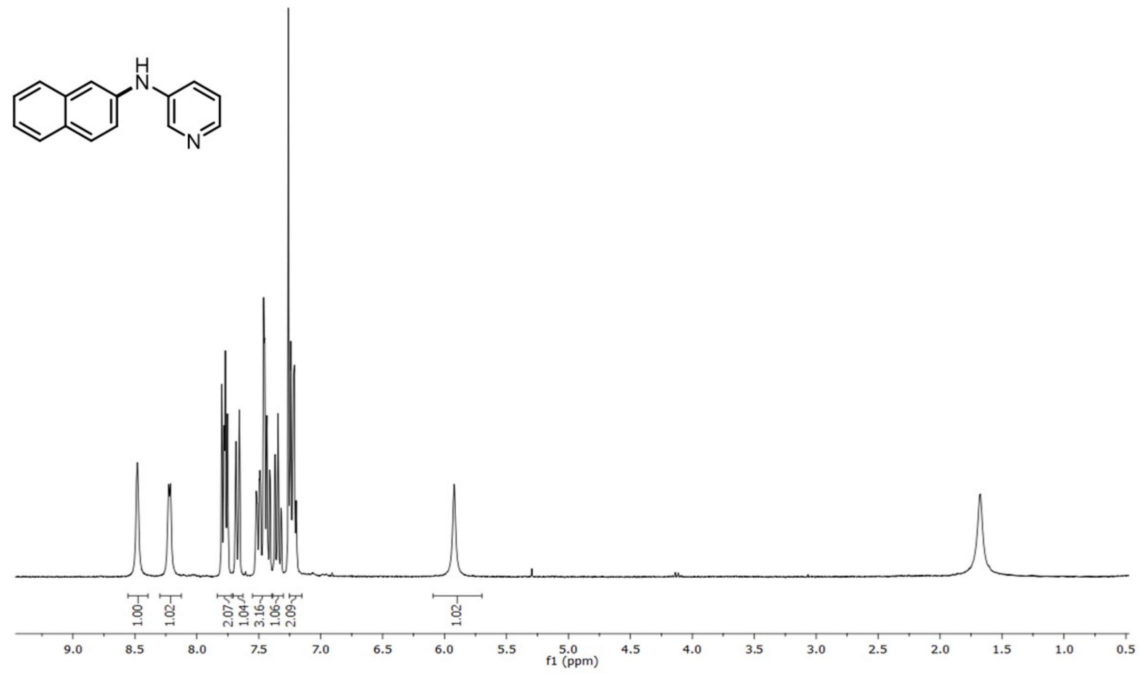

^{13}C{^{1}H} NMR spectrum of *N*-(naphthalen-2-yl)pyridyl-3-amine, 6a, (75 MHz, $CDCl_3$, 298 K)**.**

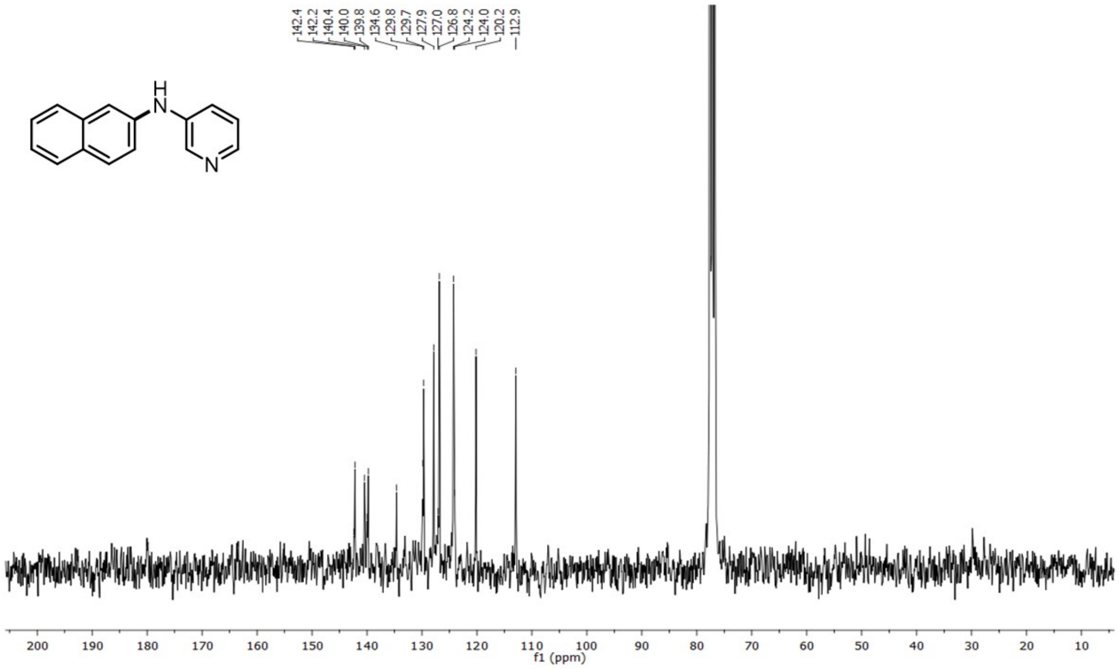

^{1}H NMR spectrum of *N*(Naphthalen-1-yl)pyridine-2-amine, 6b, (300 MHz, $CDCl_3$, 298 K)**.**

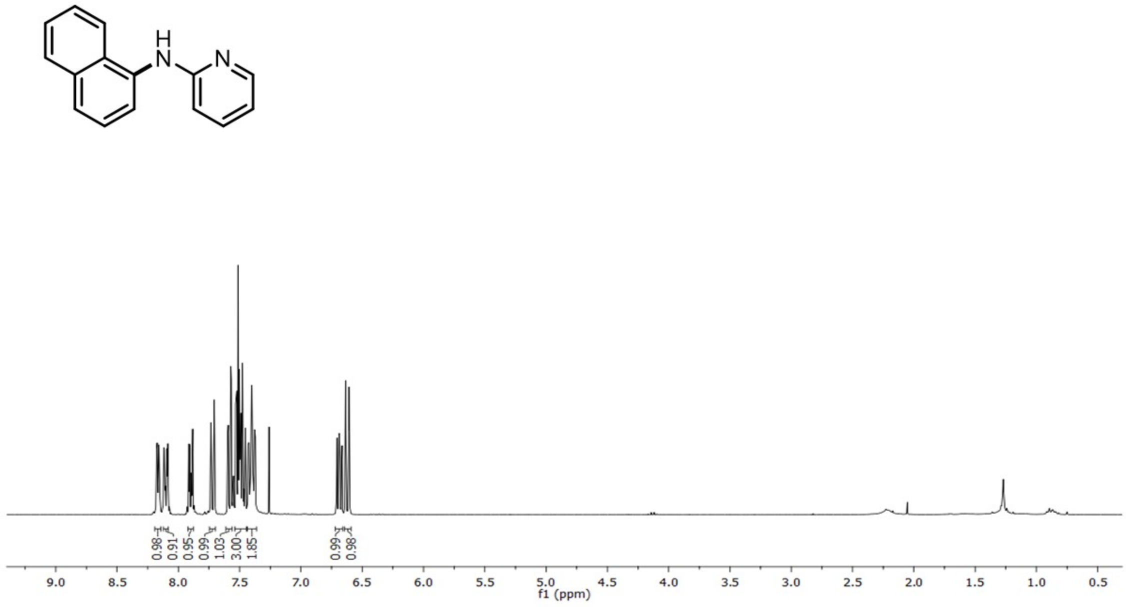

^{1}H NMR spectrum of *N*-(naphthalen-1-yl)pyrimidin-2-amine, 6c, (300 MHz, $CDCl_3$, 298 K)**.**

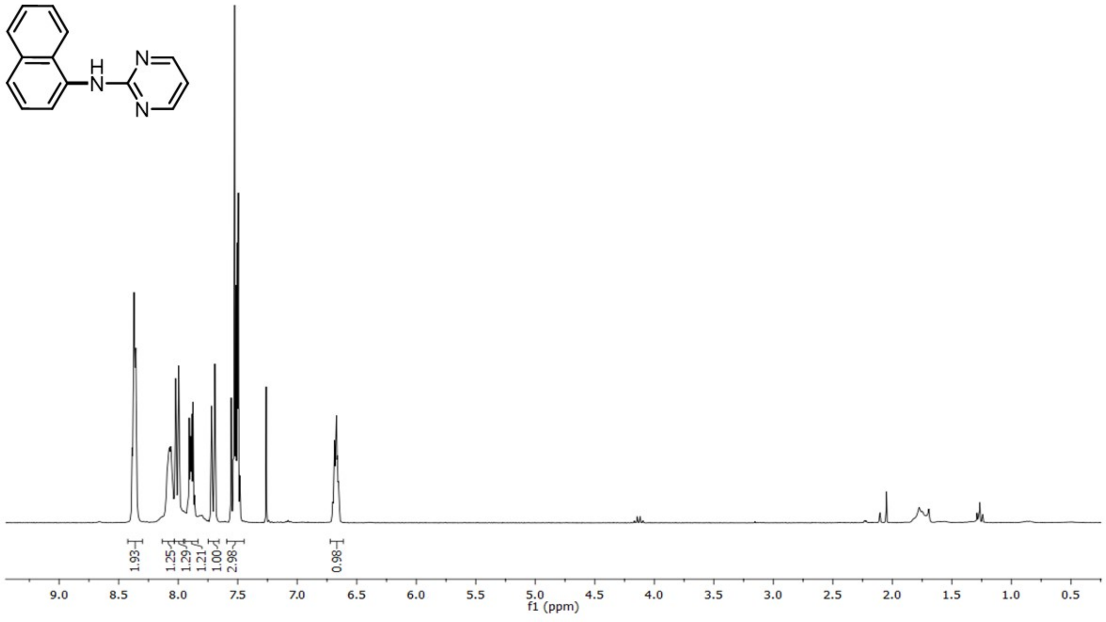

^{1}H NMR spectrum of *N*-(7-Methoxynaphthalen-2-yl)pyrimidin-2-amine, 6d, (300 MHz, $CDCl_3$, 298 K)

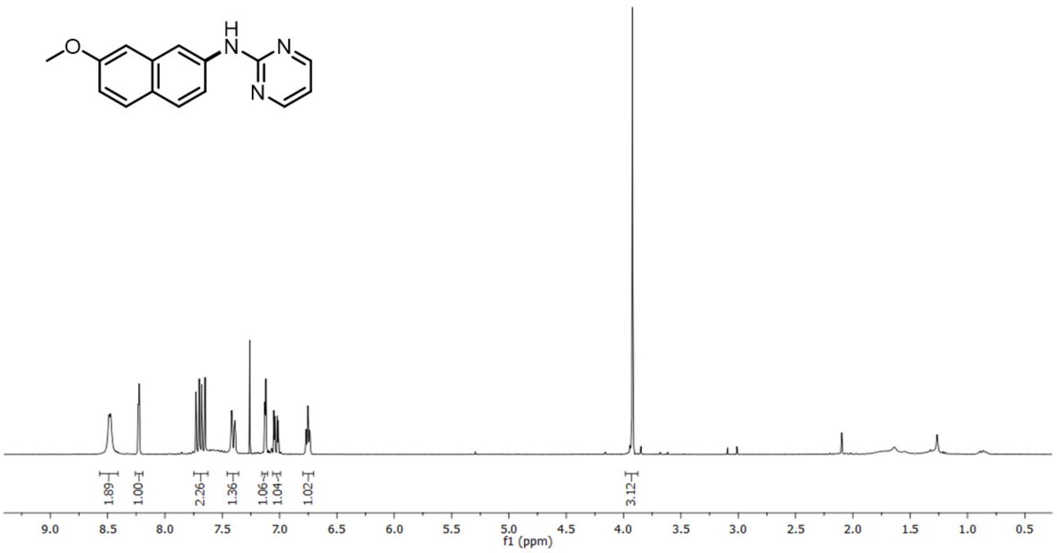

^{13}C{^{1}H} NMR spectrum of *N*-(7-Methoxynaphthalen-2-yl)pyrimidin-2-amine, 6d, (75 MHz, $CDCl_3$, 298 K)

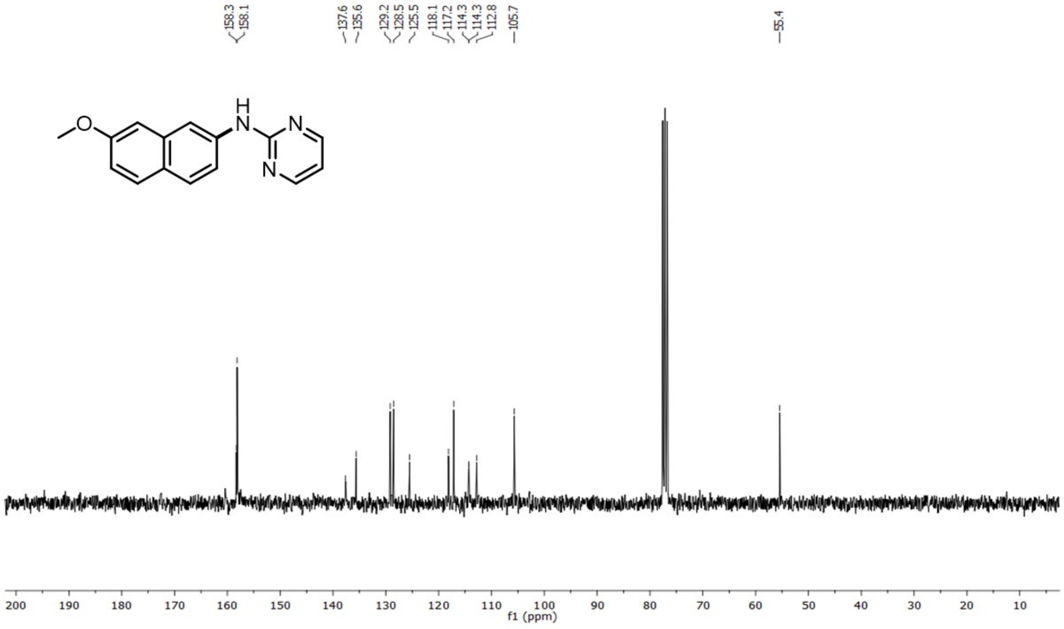

¹H NMR spectrum of *N*-(pyrazin-2-yl)quinoline-6-amine, 6e, (300 MHz, $CDCl_3$, 298 K)**.**

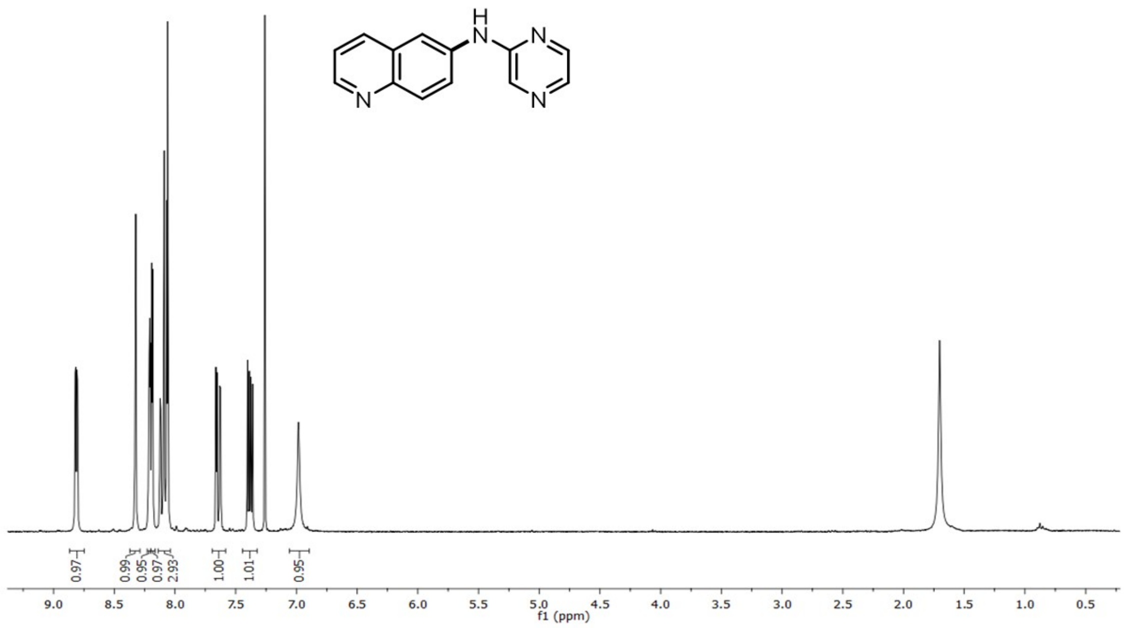

^{1}H NMR spectrum of *N*-(Naphthalen-1yl)pyrazin-2-amine, 6f, (300 MHz, $CDCl_3$, 298 K)**.**

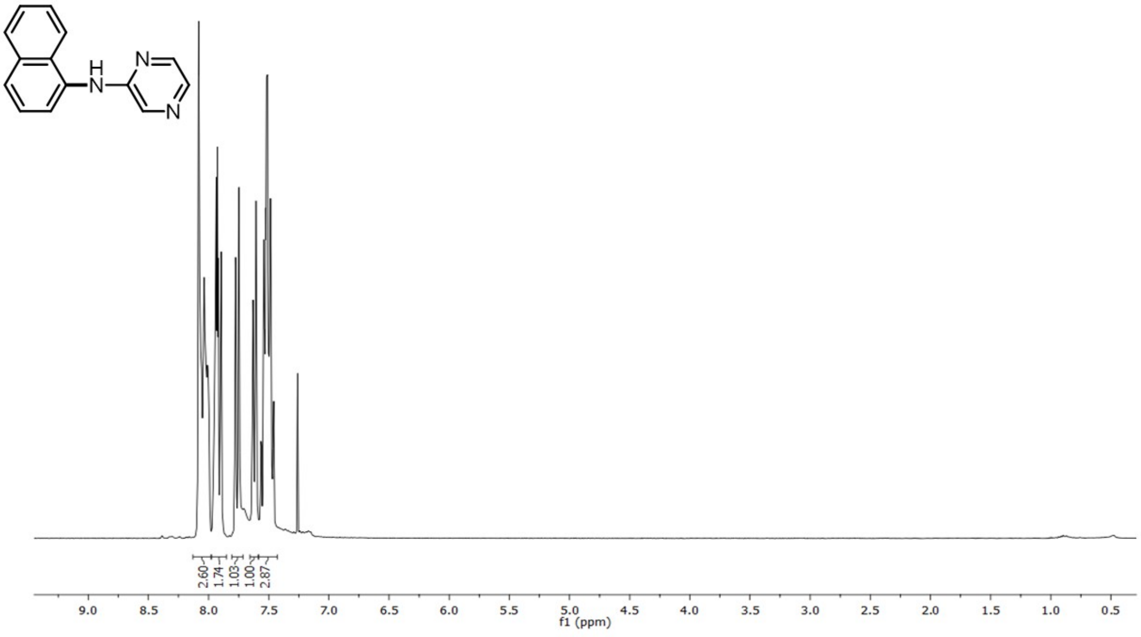

^{13}C{^{1}H} NMR spectrum of *N*-(Naphthalen-1yl)pyrazin-2-amine, 6f, (75 MHz, $CDCl_3$, 298 K)

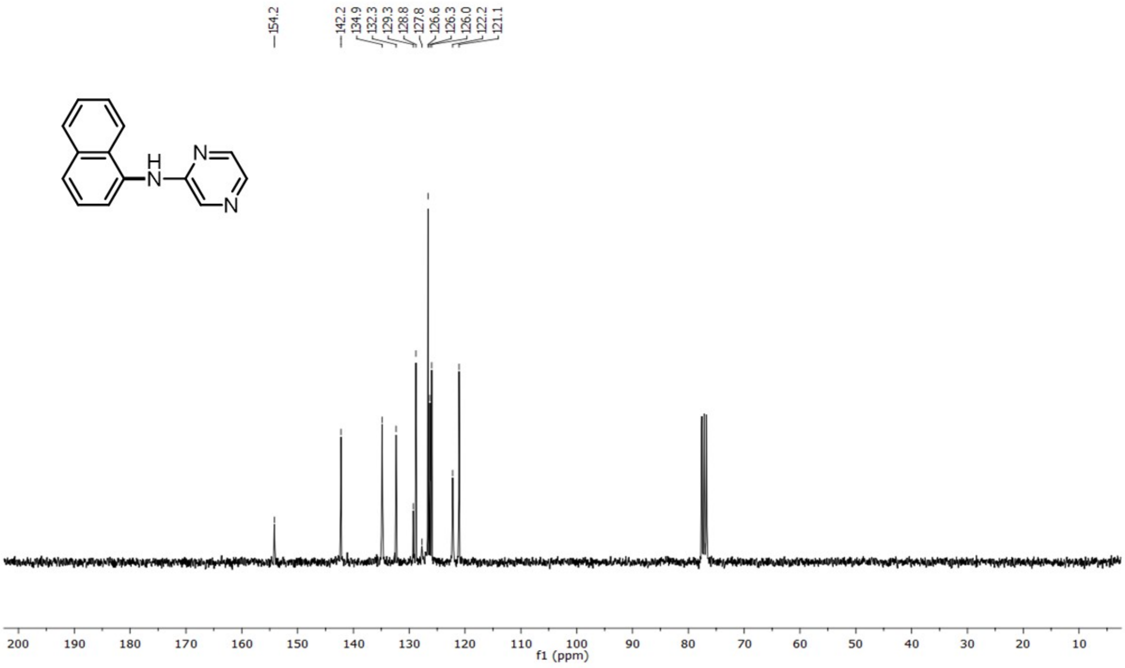

^{1}H NMR spectrum of N-(7-Methoxynaphthalen-2-yl)oxazol-2-amine, 6g, (300 MHz, $CDCl_3$, 298 K)

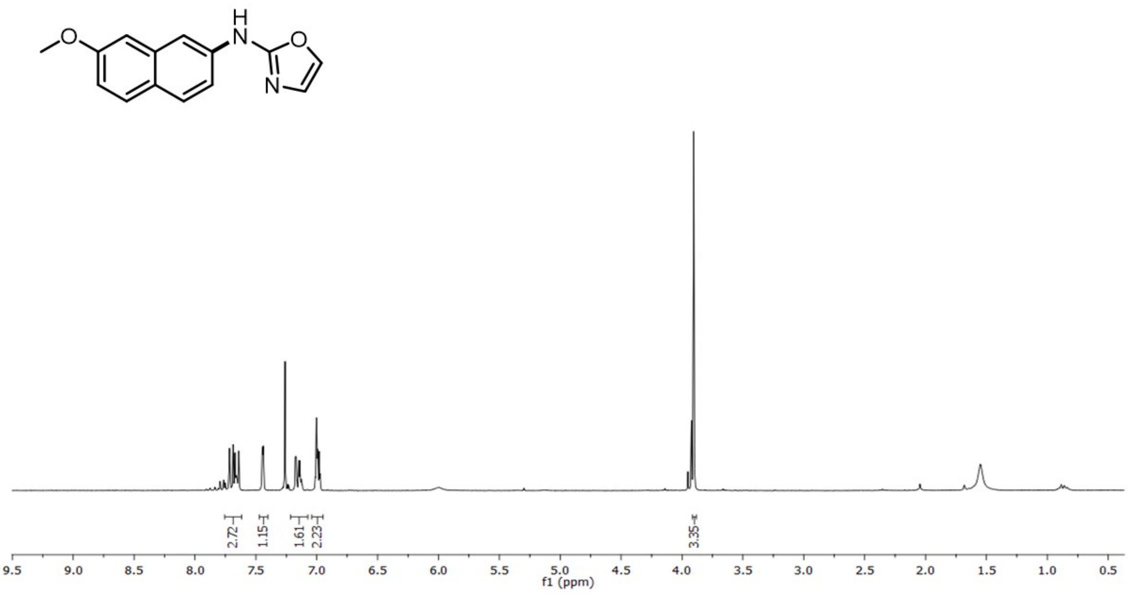

$^{13}C\{^{1}H\}$ NMR spectrum of N-(7-Methoxynaphthalen-2-yl)oxazol-2-amine, 6g, (75 MHz, $CDCl_3$, 298 K)

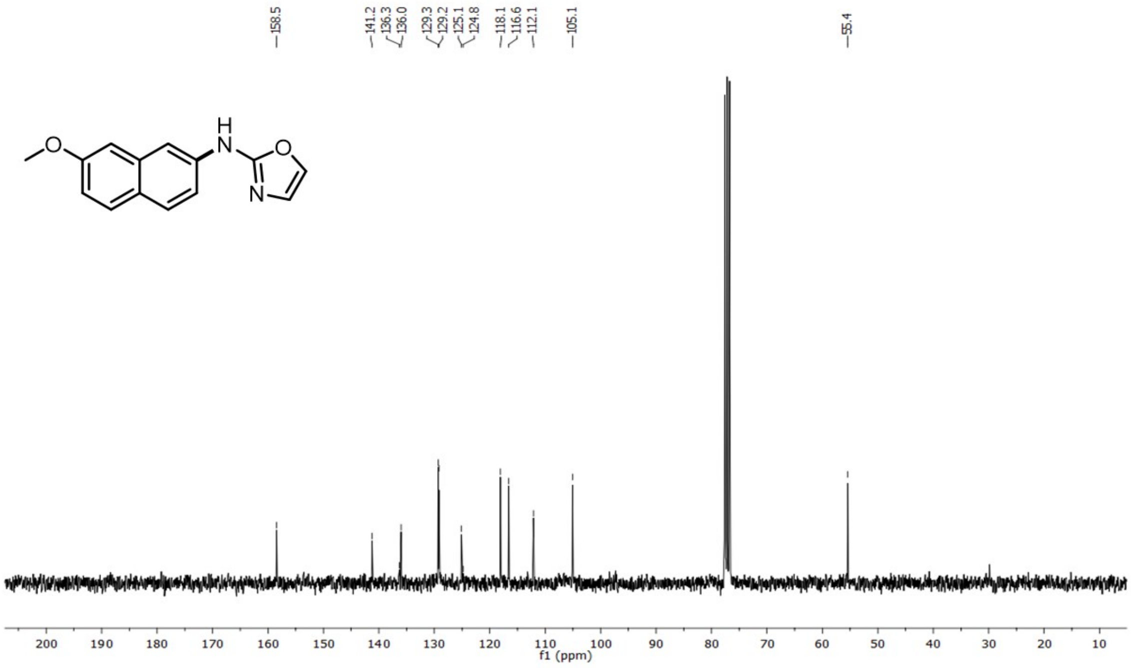

^{1}H NMR spectrum of 6-(benzo[*d*]oxazol-2-ylamino)-2-naphthonitrile, 6h, (300 MHz, DMSO-d$_6$, 298 K)

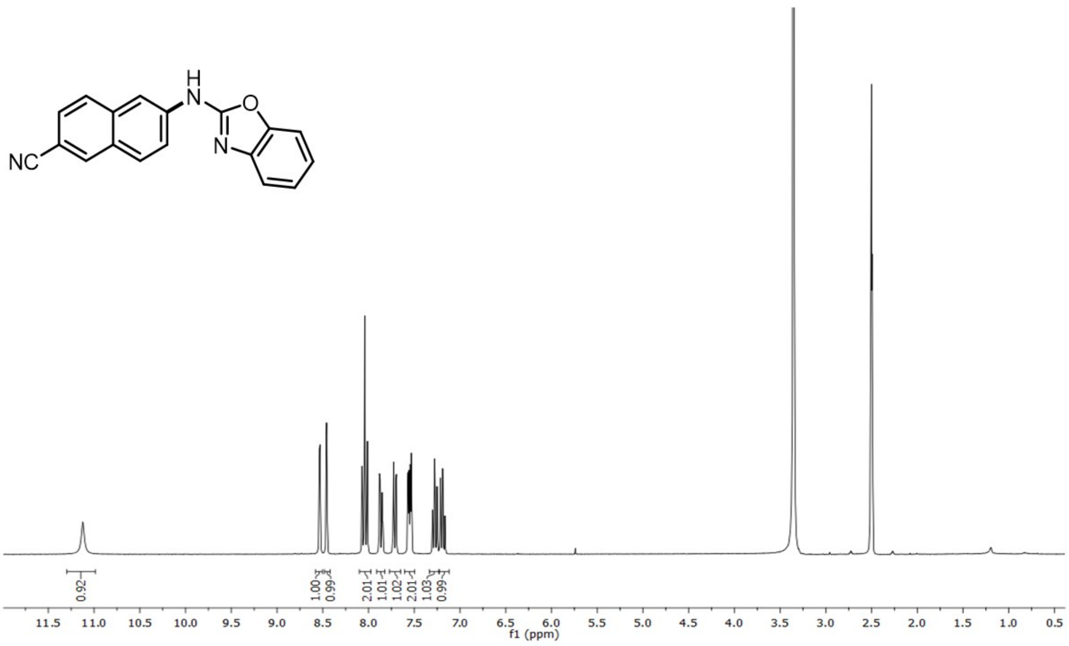

^{13}C{^{1}H} NMR spectrum of 6-(benzo[d]oxazol-2-ylamino)-2-naphthonitrile, 6h, (75 MHz, DMSO-d$_6$, 298 K)

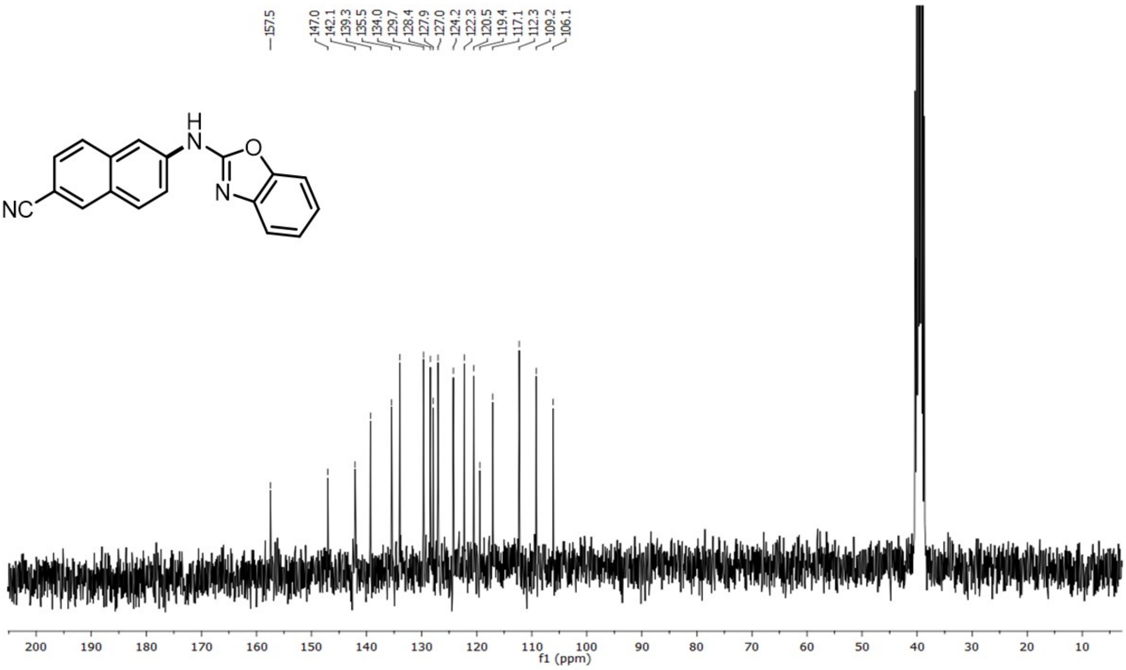

¹H NMR spectrum of 7-Methoxy-*N*-(pyridine-4-ylmethyl)naphthalen-2-amine, 6i, (300 MHz, $CDCl_3$, 298 K)

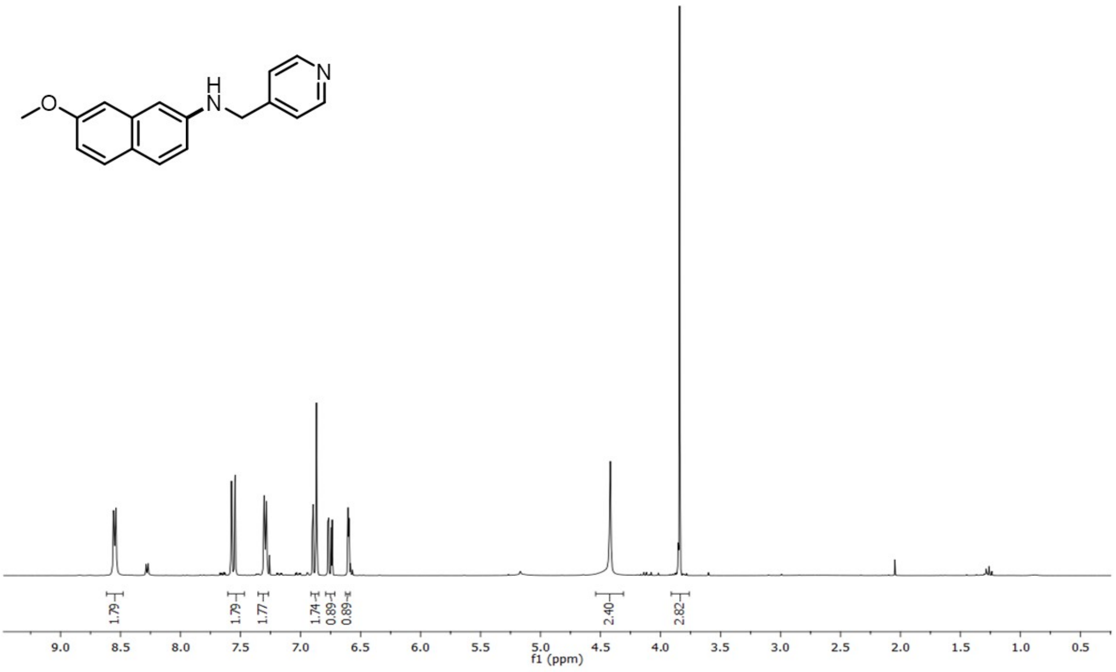

¹³C{¹H} NMR spectrum of 7-Methoxy-*N*-(pyridine-4-ylmethyl)naphthalen-2-amine, 6i, (75 MHz, $CDCl_3$, 298 K)

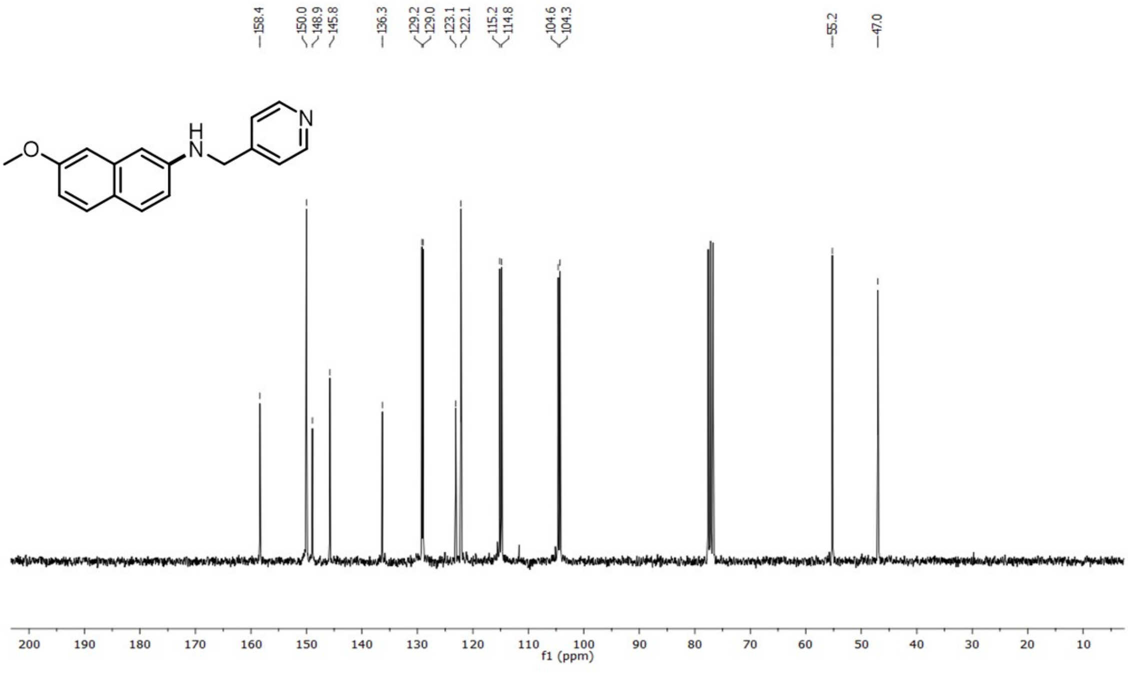

^{1}H NMR spectrum of *N*-(Pyridin-2-ylmethyl)naphthalen-1-amine, 6j, (300 MHz, $CDCl_3$, 298 K)

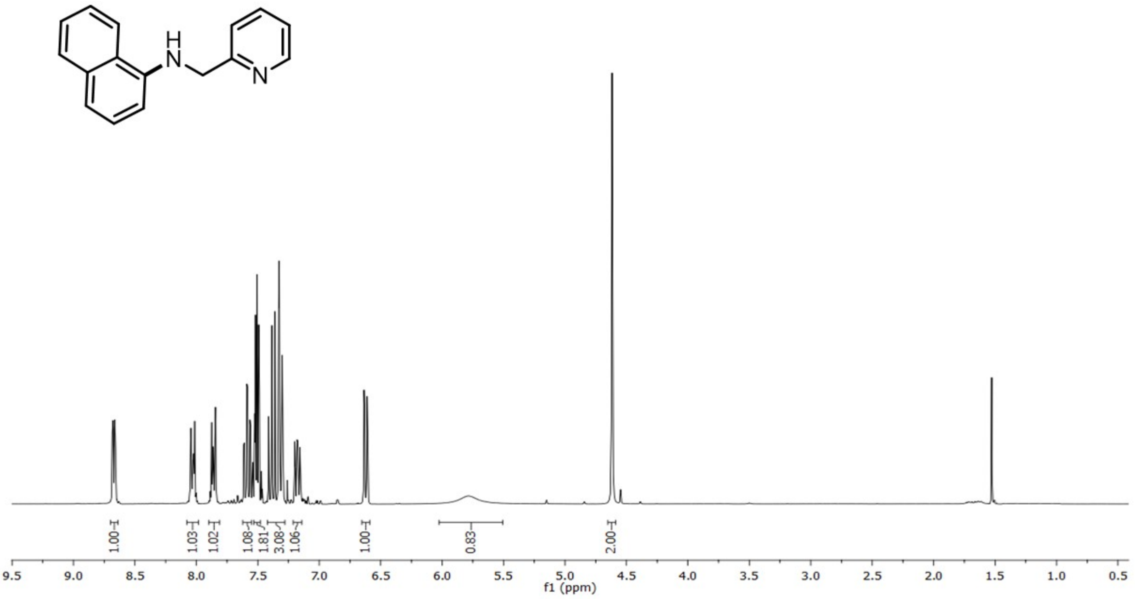

^{1}H NMR spectrum of 1-(Naphthalen-2-yl)-1*H*-pyrrole, 7a, (300 MHz, $CDCl_3$, 298 K)

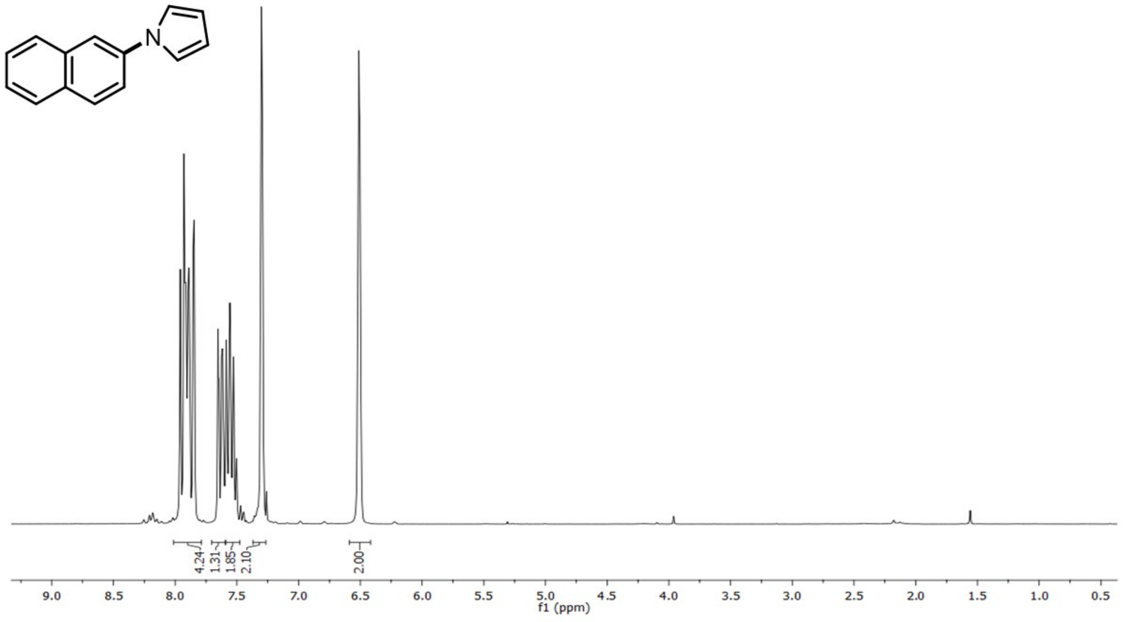

^{1}H NMR spectrum of 6-(1*H*-Pyrazol-1-yl)-2-naphthonitrile, 7b, (300 MHz, $CDCl_3$, 298 K)**.**

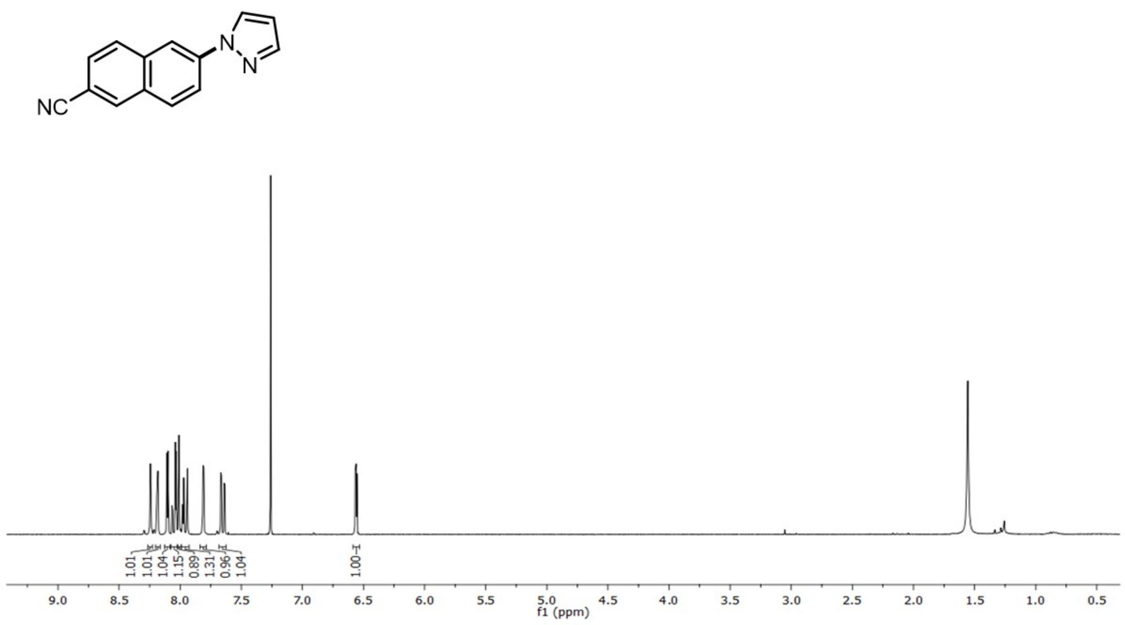

^{13}C{^{1}H} NMR spectrum of 6-(1*H*-Pyrazol-1-yl)-2-naphthonitrile, 7b, (75 MHz, $CDCl_3$, 298 K)**.**

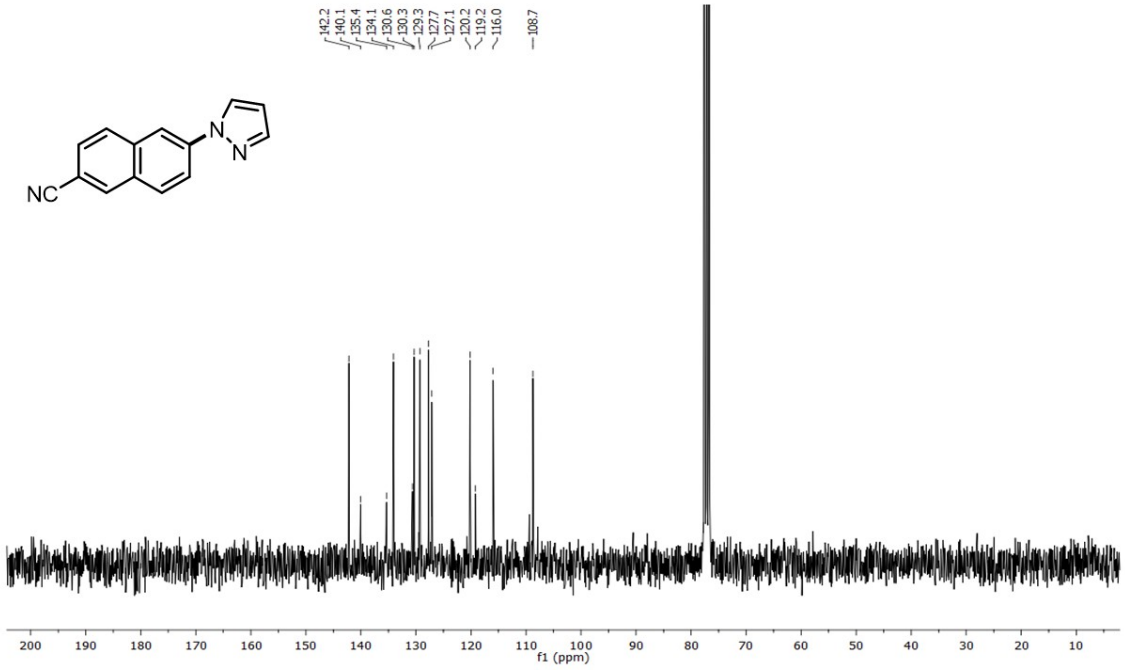

^{1}H NMR spectrum of 9-(Naphthalen-2-yl)-9*H*-carbazole, 7c, (300 MHz, $CDCl_3$, 298 K)

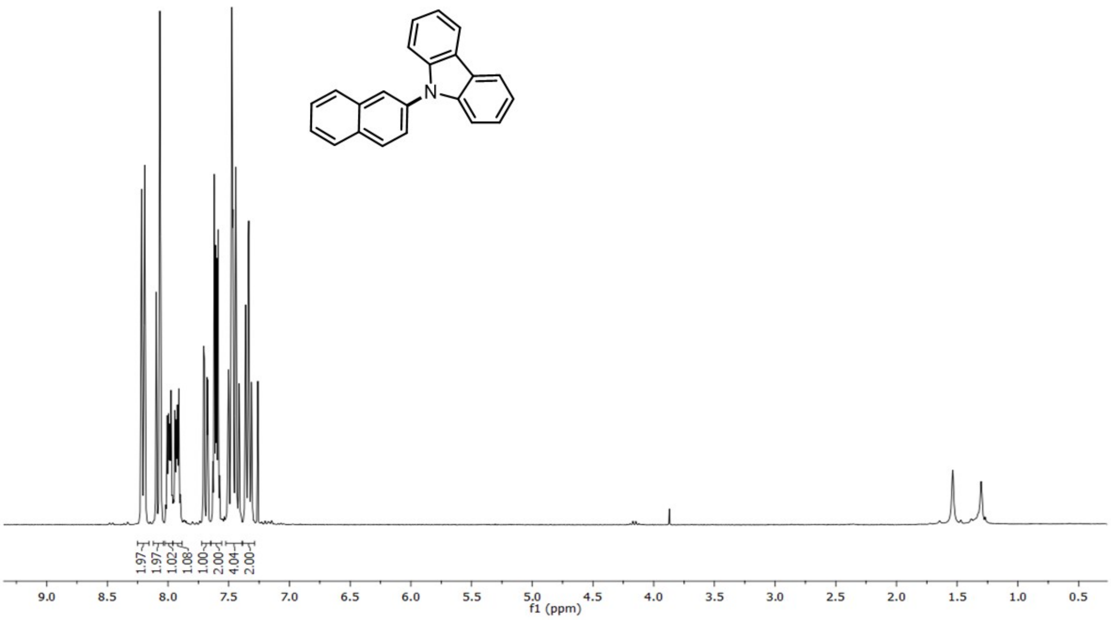

$^{13}C\{^{1}H\}$ NMR spectrum of 9-(Naphthalen-2-yl)-9*H*-carbazole, 7c, (75 MHz, $CDCl_3$, 298 K)

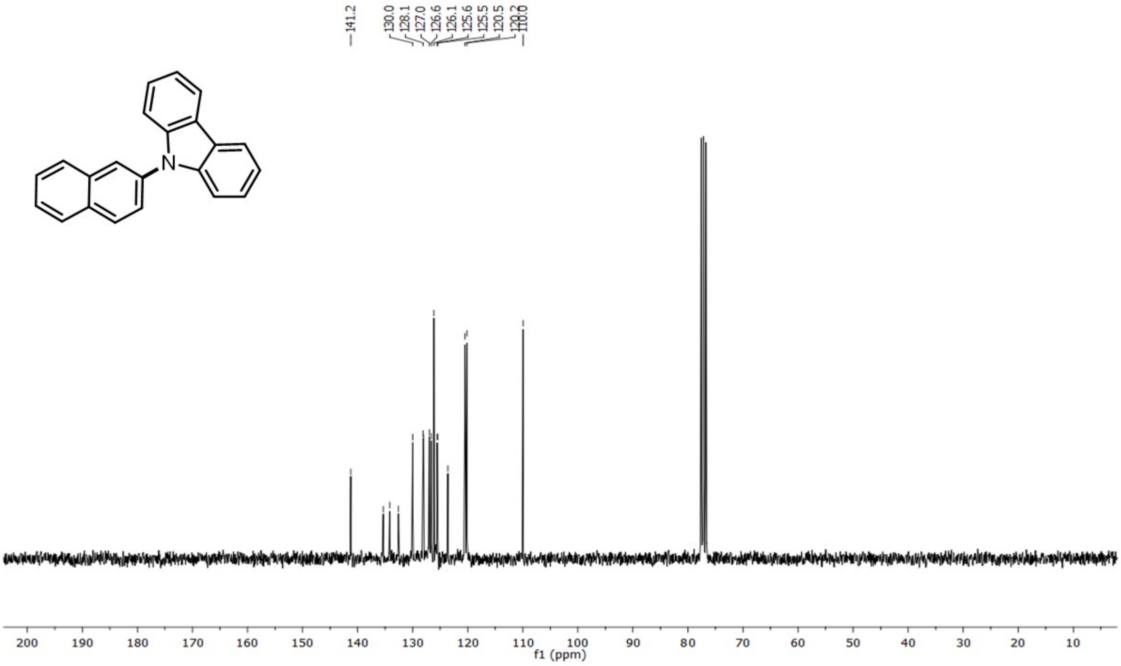

^{1}H NMR spectrum of 1-(Naphthalen-2-yl)-1*H*-indole, 7d, (300 MHz, $CDCl_3$, 298 K)

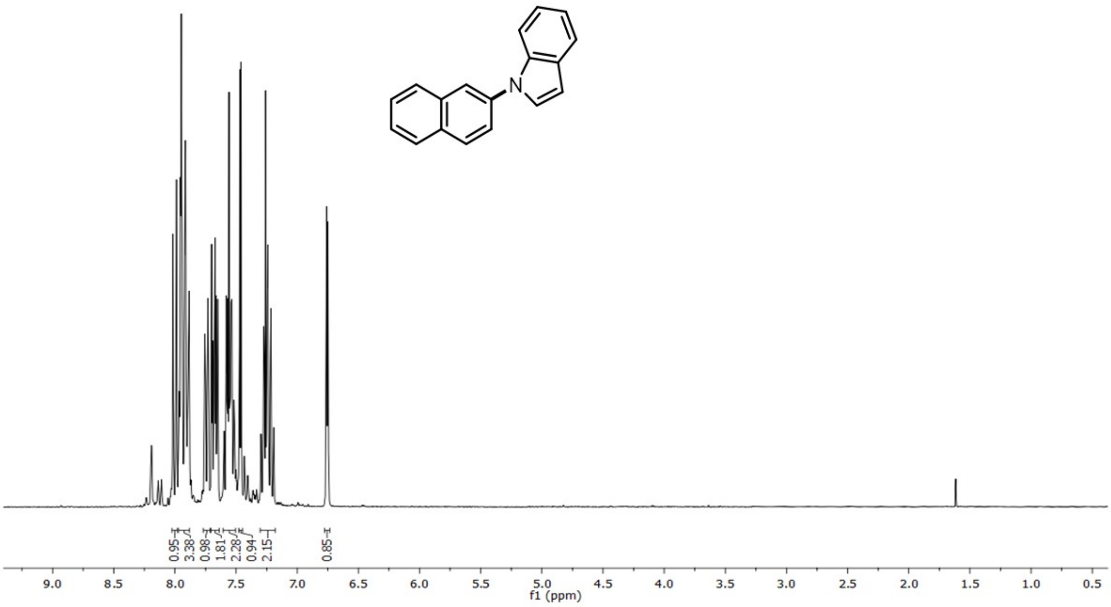

^{1}H NMR spectrum of 1-(7-methoxynaphthalen-2-yl)-3-methyl-1*H*-indole, 7e, (300 MHz, $CDCl_3$, 298 K)

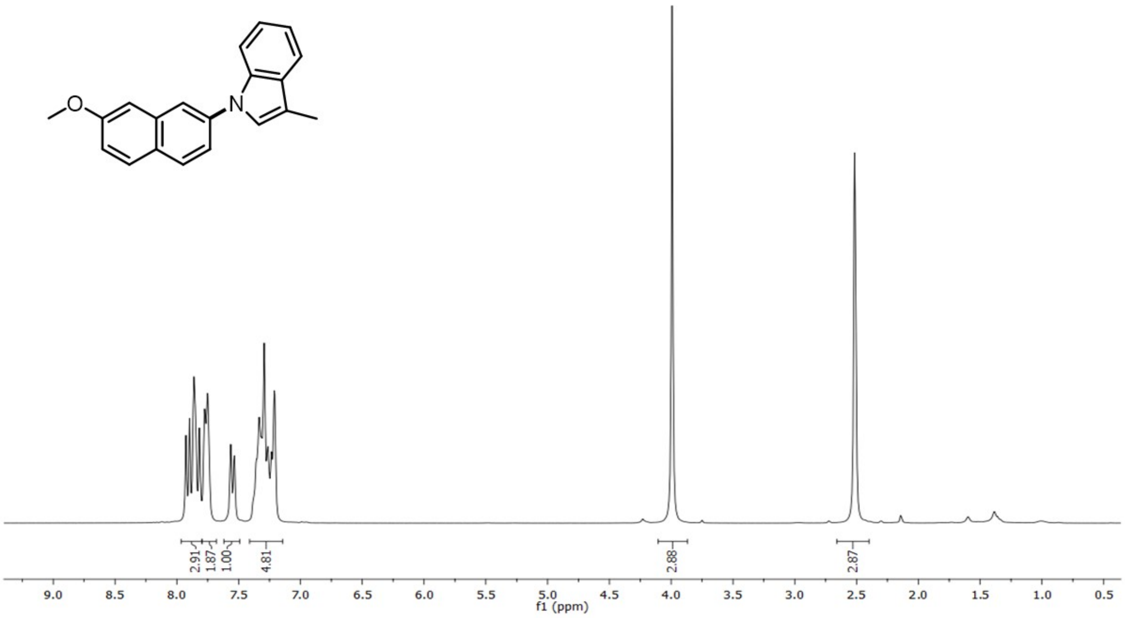

^{13}C{^{1}H} NMR spectrum of 1-(7-methoxynaphthalen-2-yl)-3-methyl-1*H*-indole, 7e, (75 MHz, $CDCl_3$, 298 K)

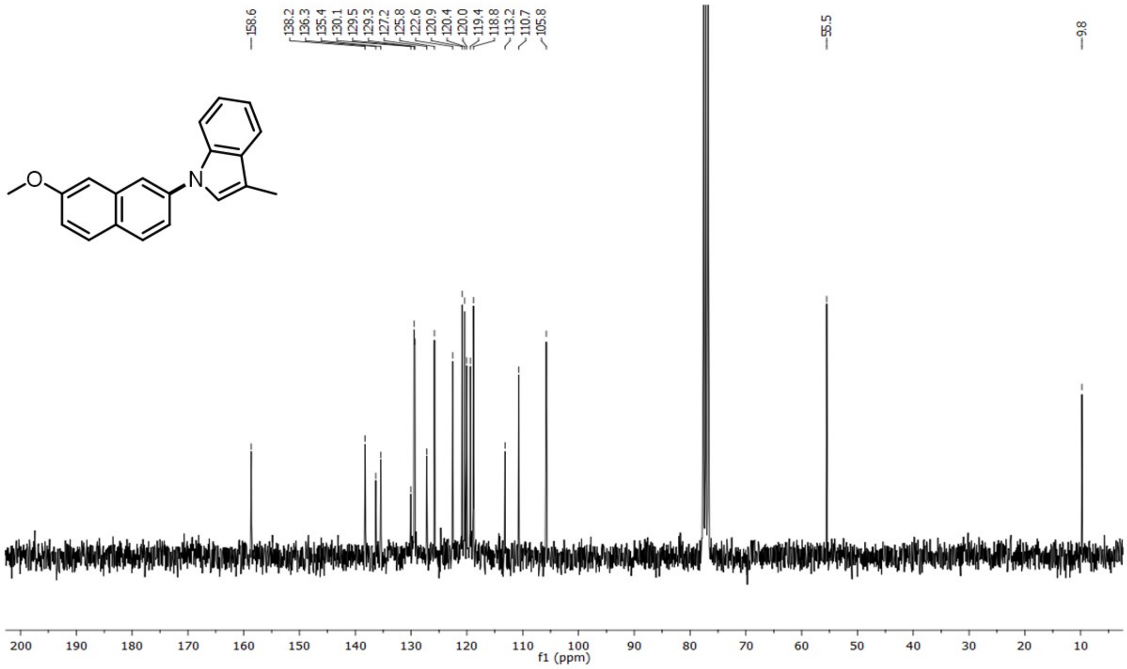

^{1}H NMR spectrum of 1-(7-methoxynaphthalen-2-yl)-1*H*-indole, 7f, (300 MHz, $CDCl_3$, 298 K)

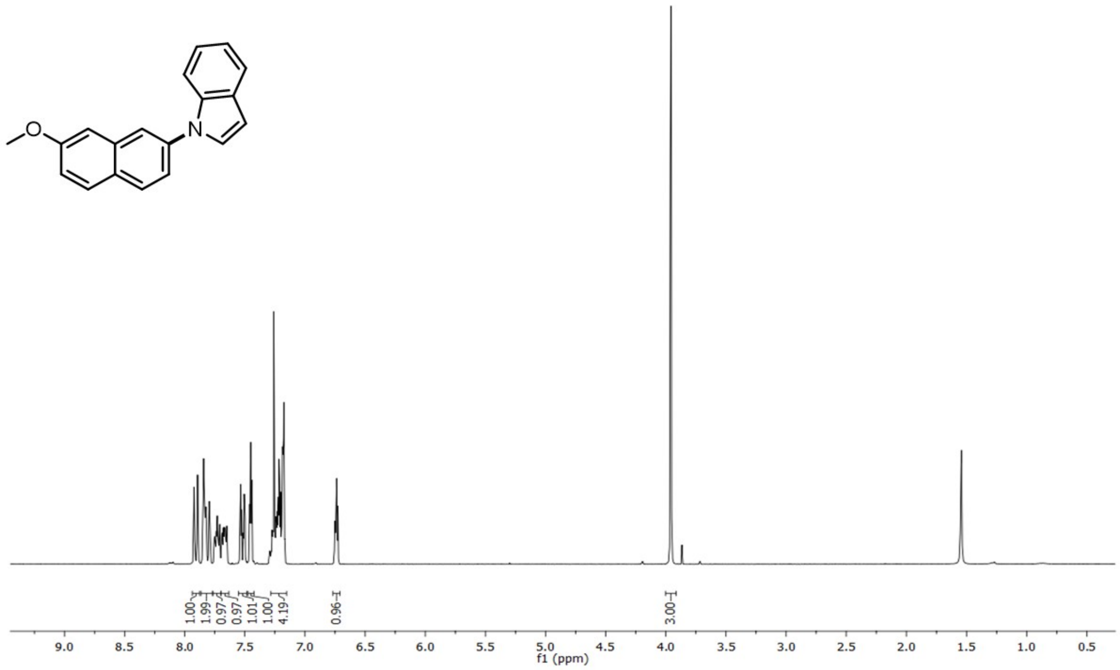

$^{13}C\{^1H\}$ NMR spectrum of 1-(7-methoxynaphthalen-2-yl)-1*H*-indole, 7f, (75 MHz, $CDCl_3$, 298 K)

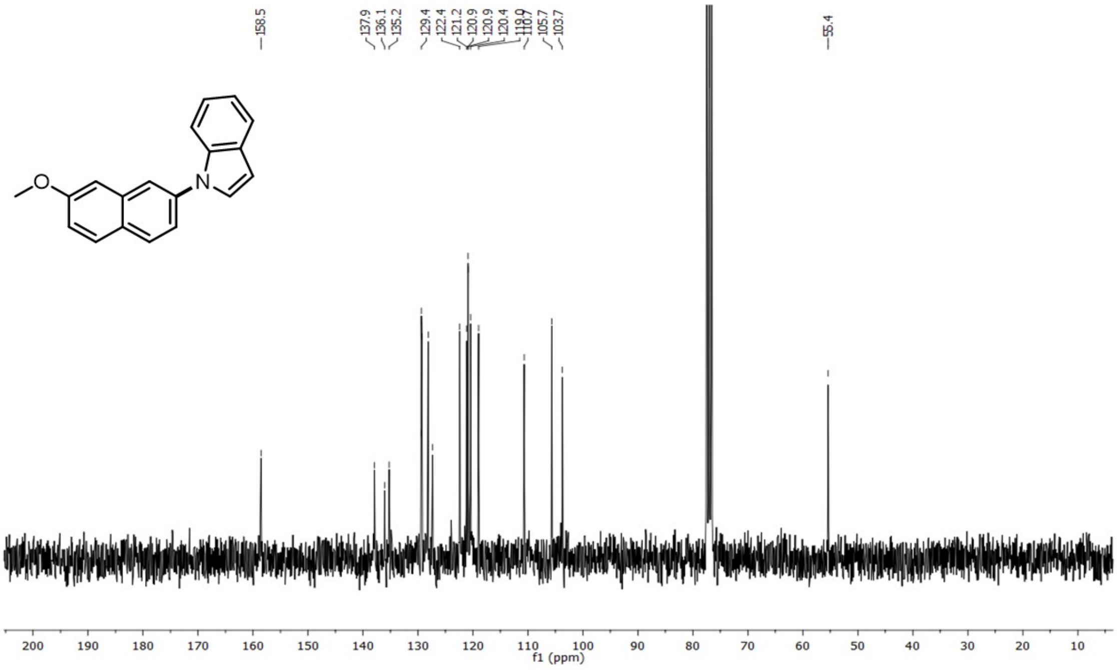

^{1}H NMR spectrum of 6-(2-methyl-1*H*-indol-1-yl)-2-naphthonitrile, 7g, (300 MHz, $CDCl_3$, 298 K)

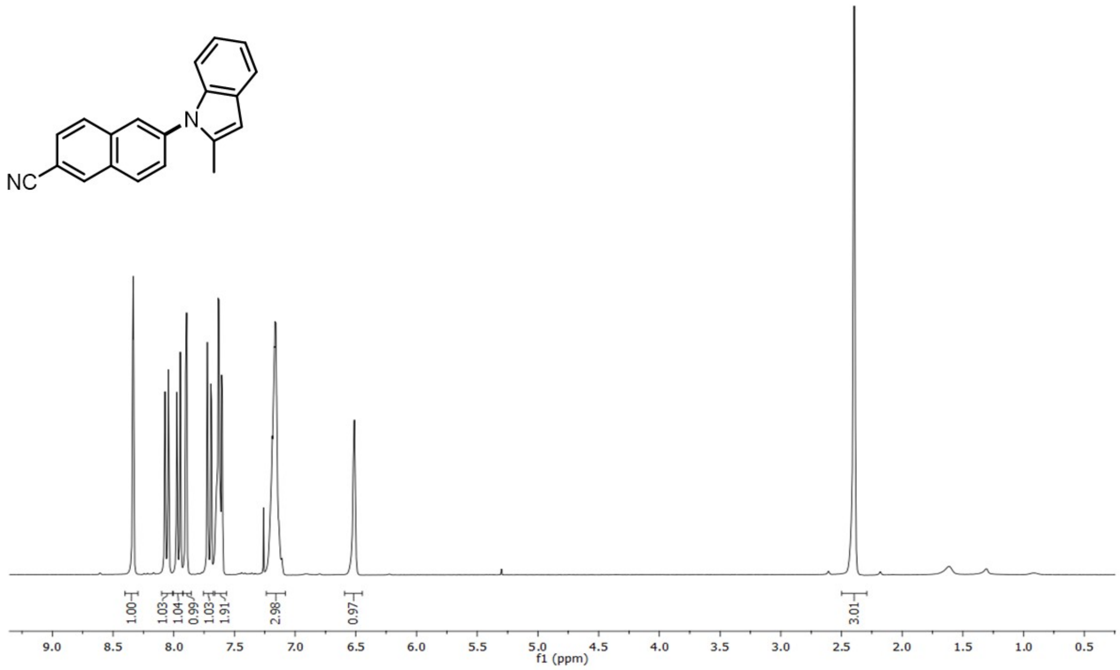

^{13}C{^{1}H} NMR spectrum of 6-(2-methyl-1*H*-indol-1-yl)-2-naphthonitrile, 7g, (75 MHz, $CDCl_3$, 298 K)

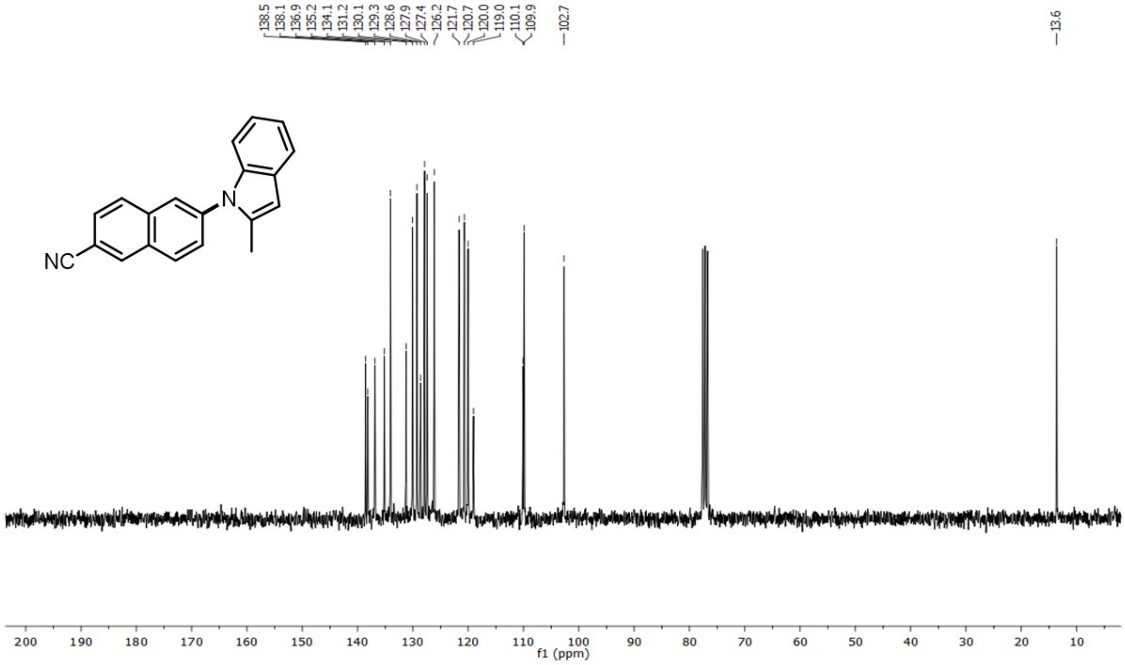

^{1}H NMR spectrum of 6-(3-methyl-1*H*-indol-1-yl)-2- naphthonitrile, 7h, (300 MHz, $CDCl_3$, 298 K)

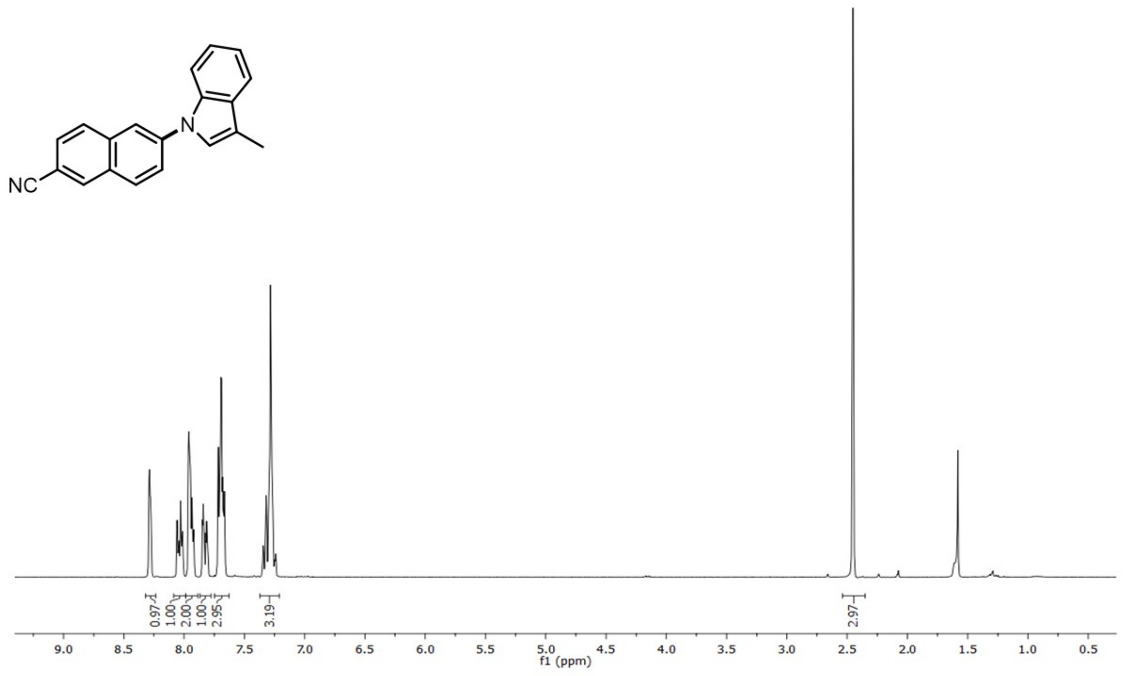

^{13}C{^{1}H} NMR spectrum of 6-(3-methyl-1*H*-indol-1-yl)-2- naphthonitrile, 7h, (75 MHz, $CDCl_3$, 298 K)

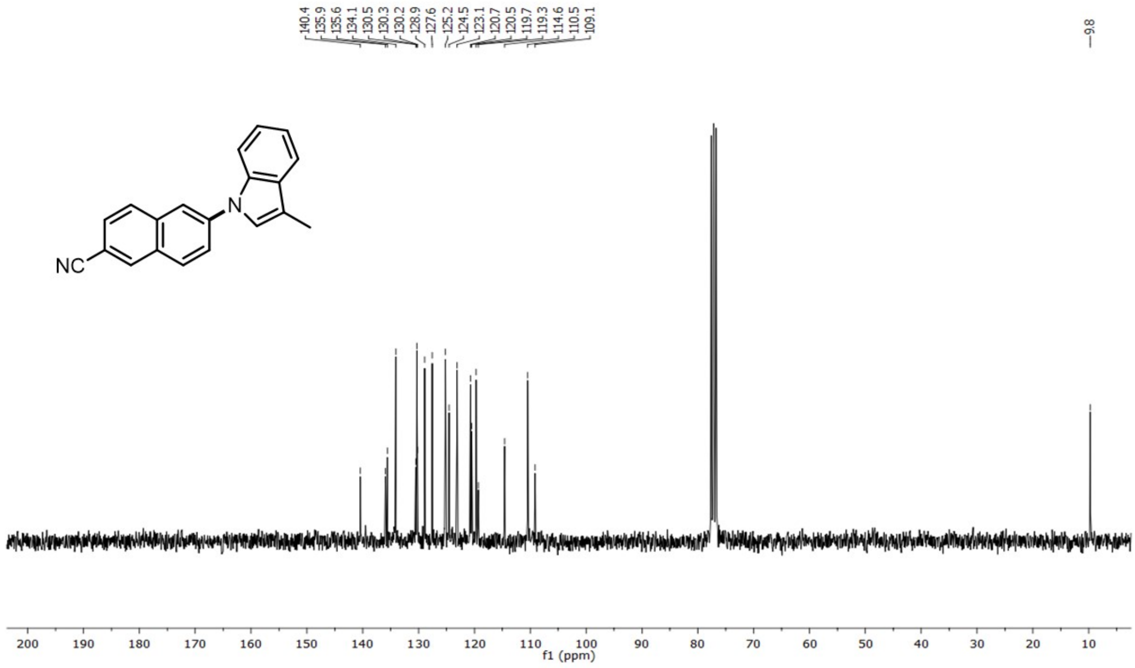

¹H NMR spectrum of methyl-6-(2,3-dimethyl-1*H*-indol-1-yl)-2-naphthoate, 7i, (300 MHz, $CDCl_3$, 298 K)

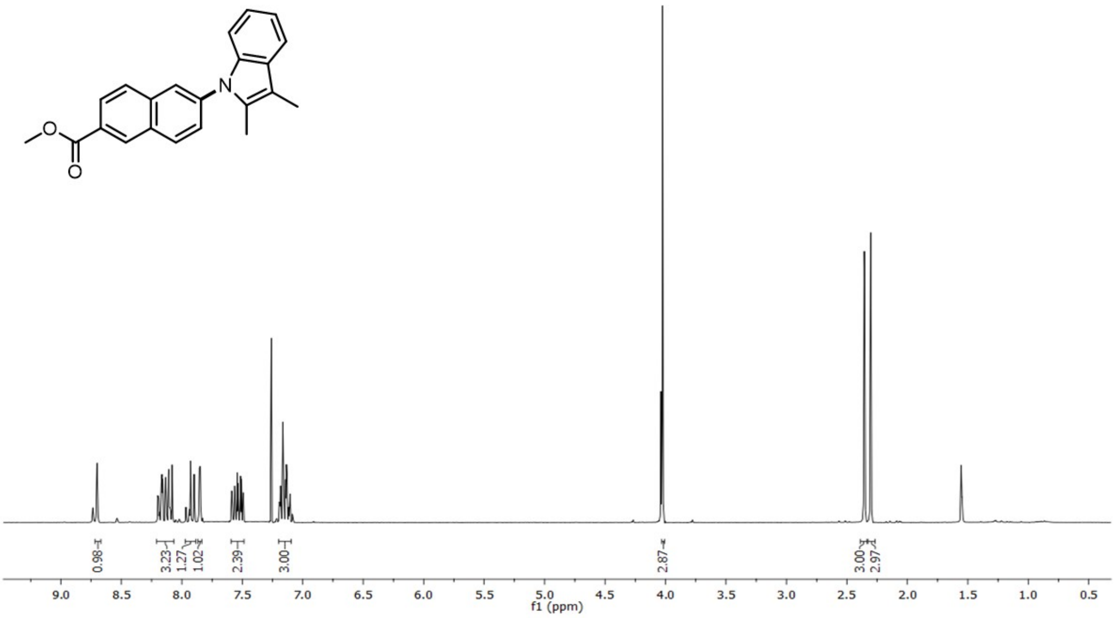

¹³C{¹H} NMR spectrum of methyl-6-(2,3-dimethyl-1*H*-indol-1-yl)-2-naphthoate, 7i, (75 MHz, $CDCl_3$, 298 K)

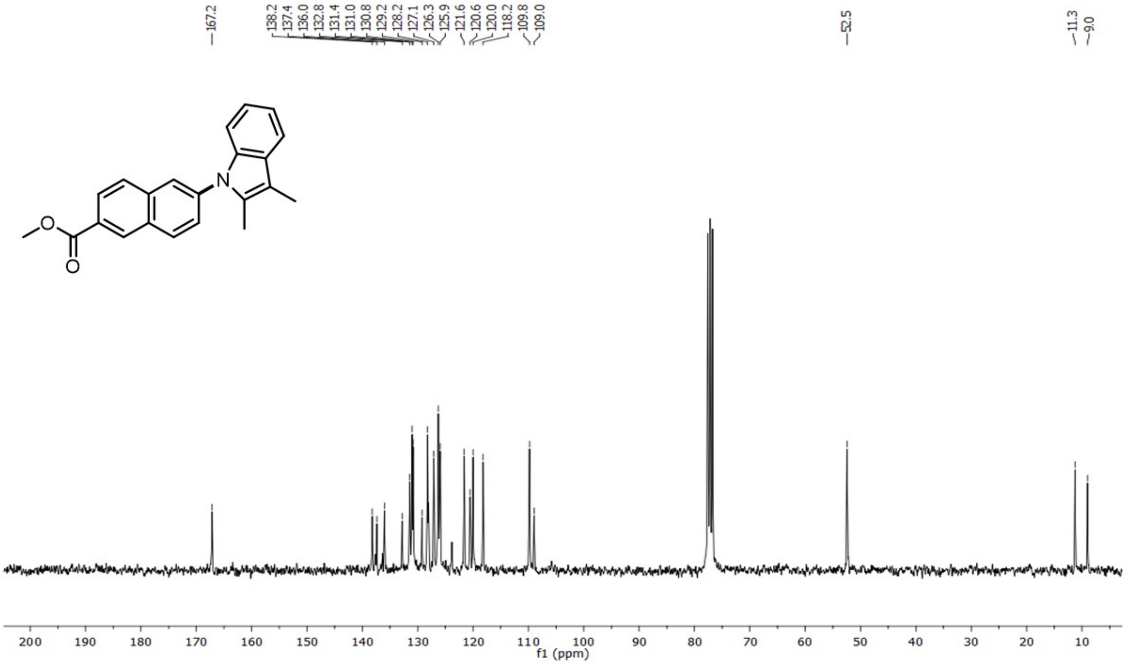

^{1}H NMR spectrum of 1-(Naphthalen-2-yl)-2-phenyl-1*H*-indole, 7j, (300 MHz, $CDCl_3$, 298 K)**.**

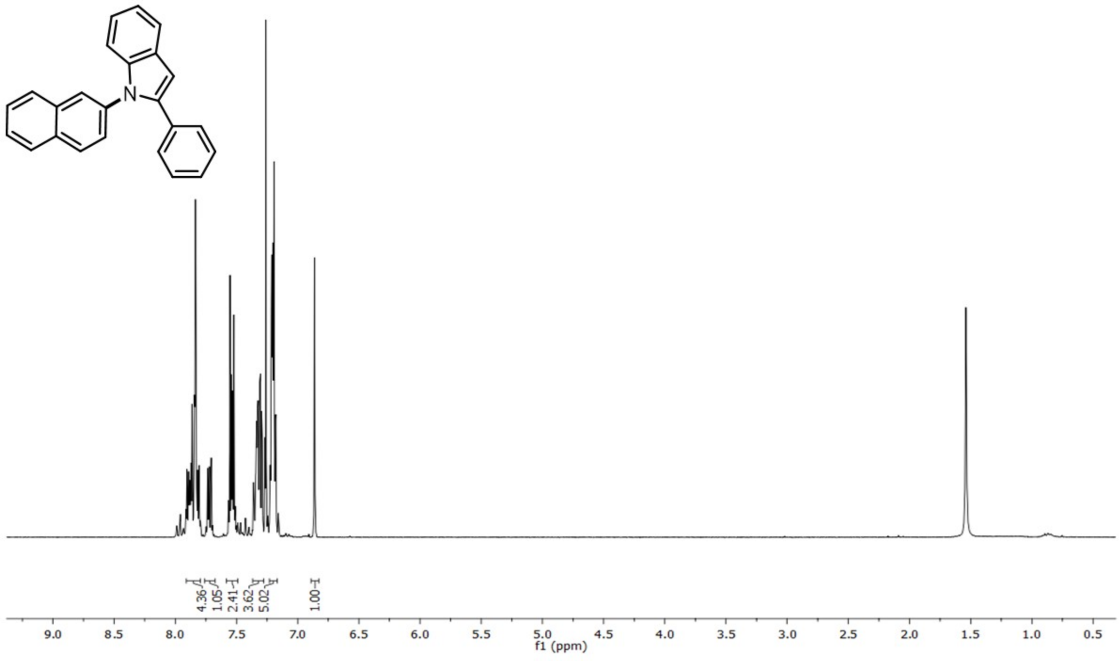

^{13}C{^{1}H} NMR spectrum of 1-(Naphthalen-2-yl)-2-phenyl-1*H*-indole, 7j, (75 MHz, $CDCl_3$, 298 K)**.**

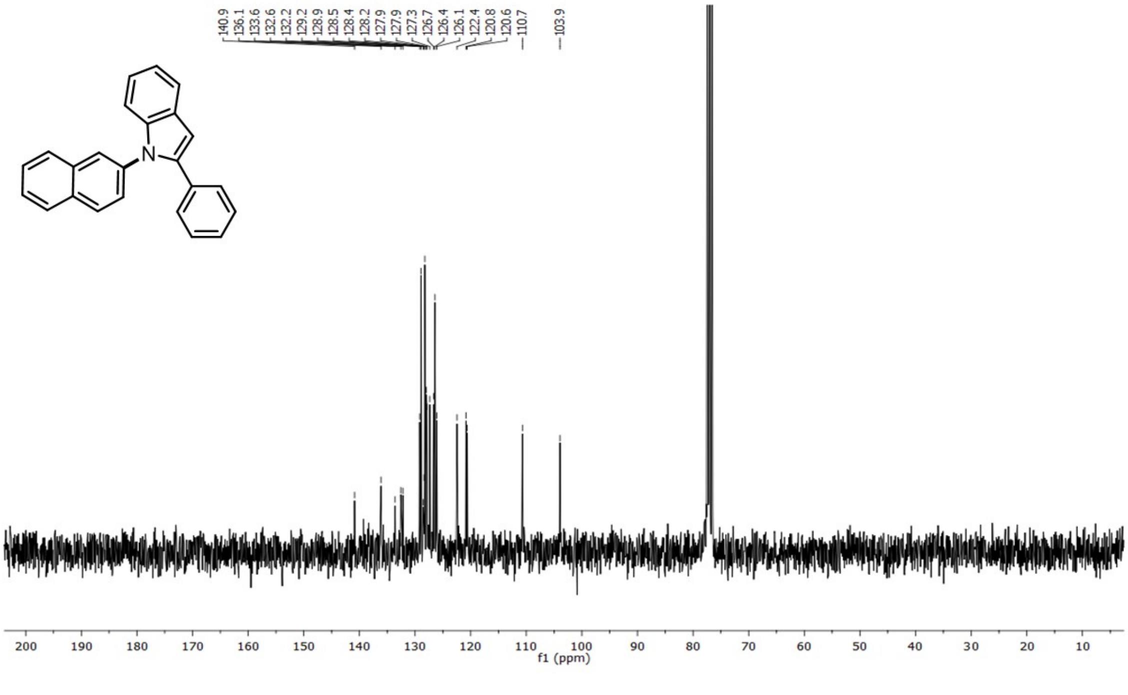

^{1}H NMR spectrum of *N*-(naphthalen-1-yl)benzamide, 8a, (300 MHz, $CDCl_3$, 298 K)

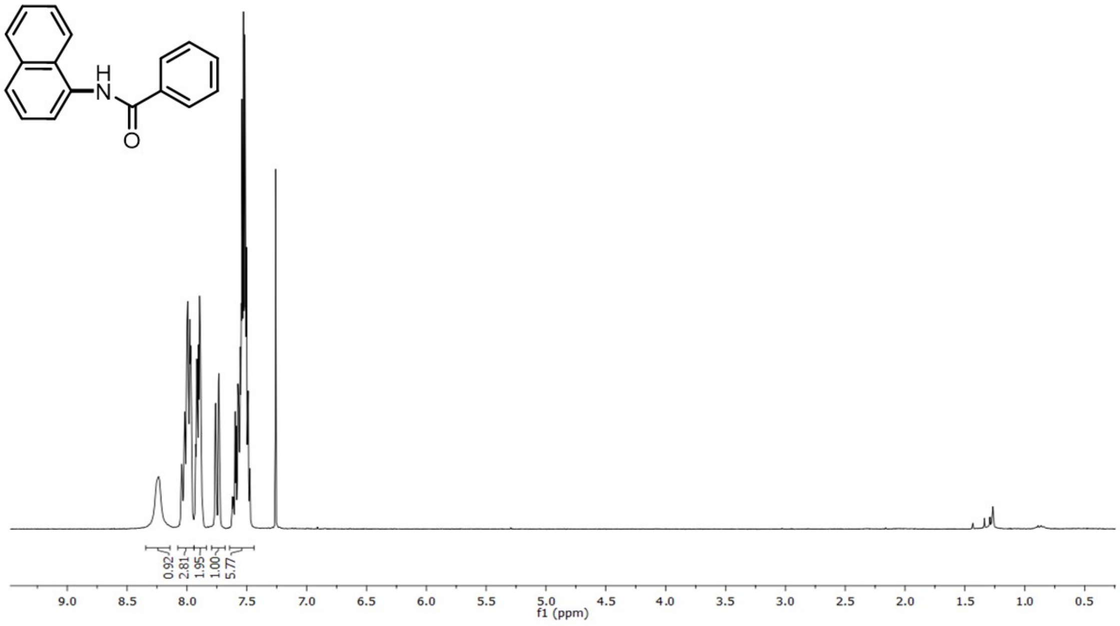

^{1}H NMR spectrum of 4-methoxy-*N*-(naphthalen-1-yl)benzamide, 8b, (300 MHz, $CDCl_3$, 298 K)

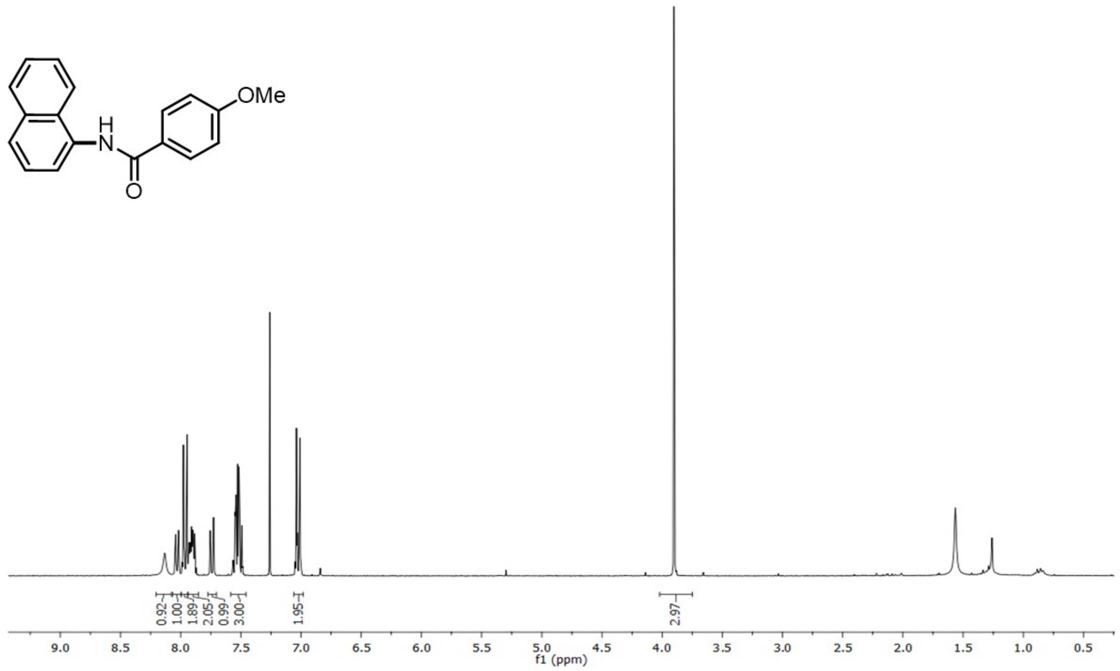

^{1}H NMR spectrum of *N*-(naphthalen-1-yl)-4-(trifluoromethyl)benzamide, 8c, (300 MHz, $CDCl_3$, 298 K)**)**

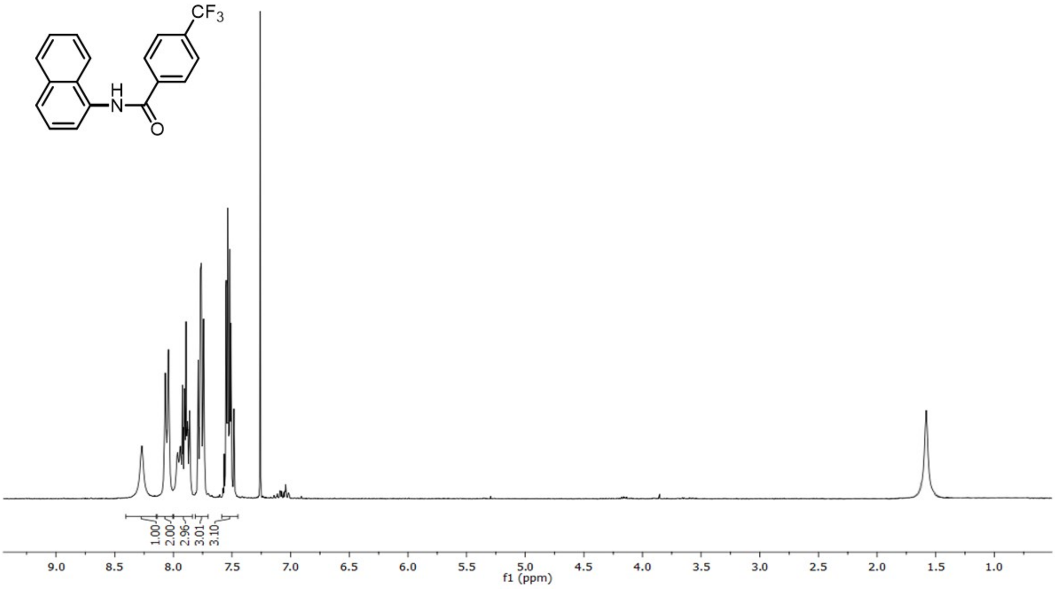

^{13}C{^{1}H} NMR spectrum of *N*-(naphthalen-1-yl)-4-(trifluoromethyl)benzamide, 8c, (75 MHz, $CDCl_3$, 298 K)

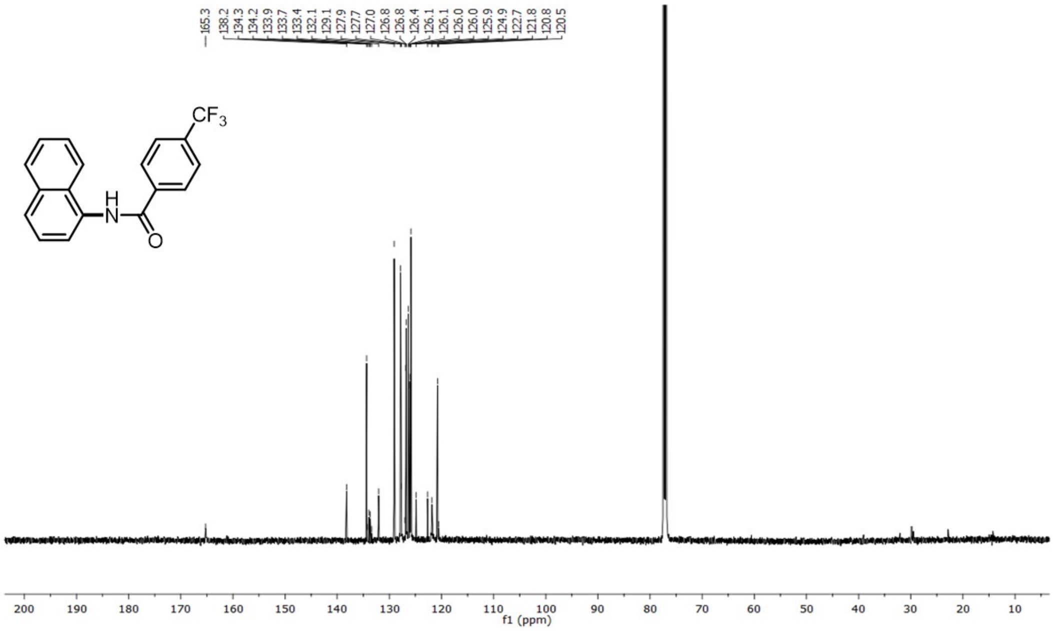

^{1}H NMR spectrum of 4-fluoro-*N*-(naphthalen-2-yl)benzamide, 8d, (300 MHz, $CDCl_3$, 298 K)

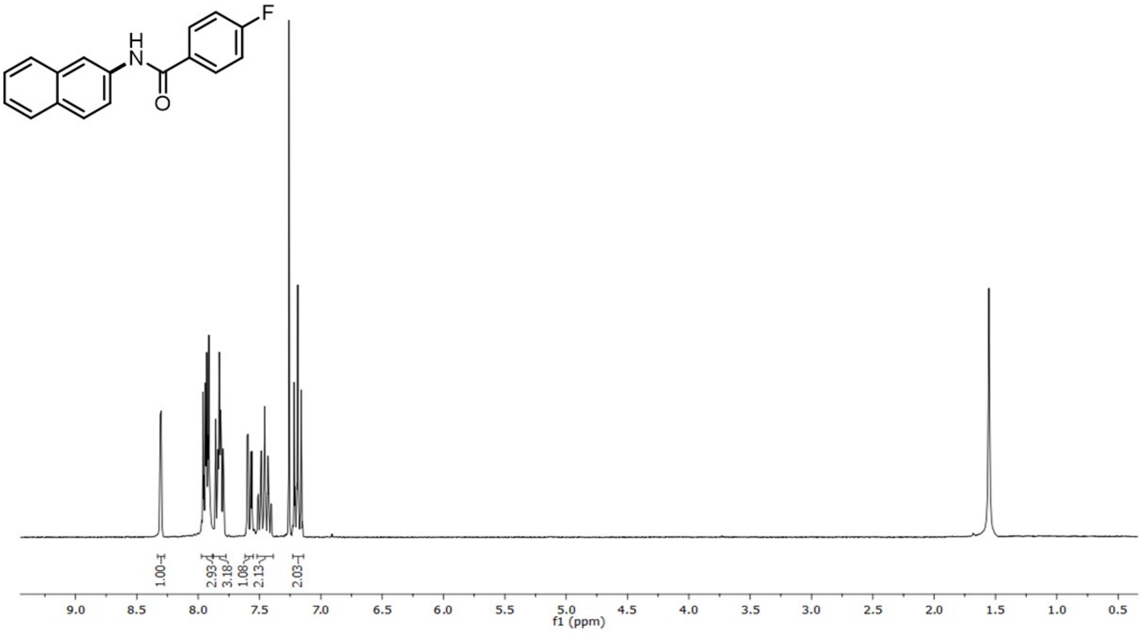

$^{13}C\{^{1}H\}$ NMR spectrum of 4-fluoro-*N*-(naphthalen-2-yl)benzamide, 8d, (75 MHz, $CDCl_3$, 298 K)

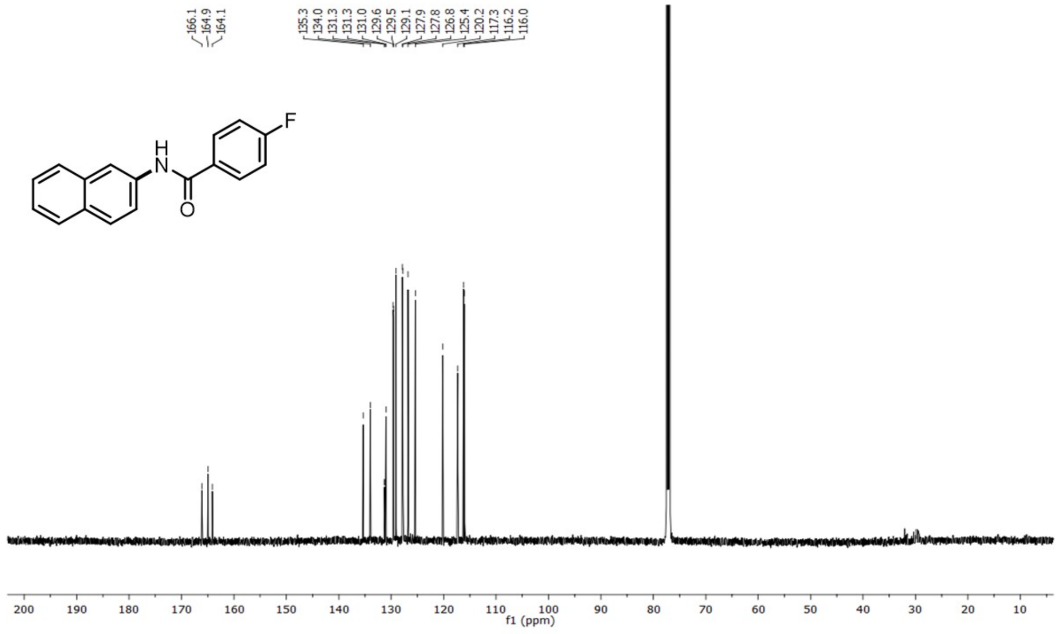

^{1}H NMR spectrum of *N*-(7-methoxynaphthalen-2-yl)formamide, 8e, (300 MHz, $CDCl_3$, 298 K)

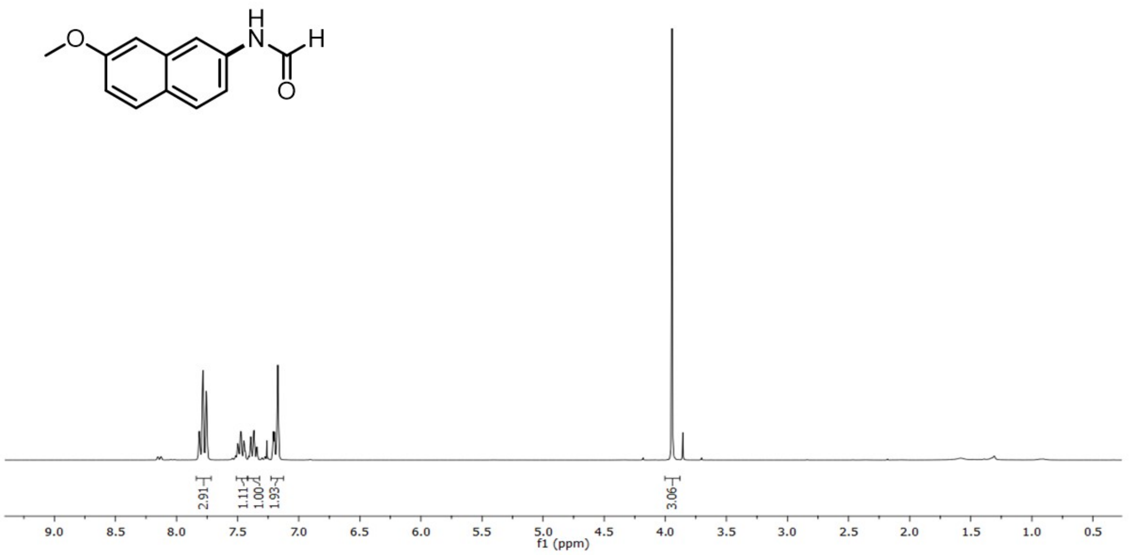

^{13}C{^{1}H} NMR spectrum of *N*-(7-methoxynaphthalen-2-yl)formamide, 8e, (75 MHz, $CDCl_3$, 298 K)

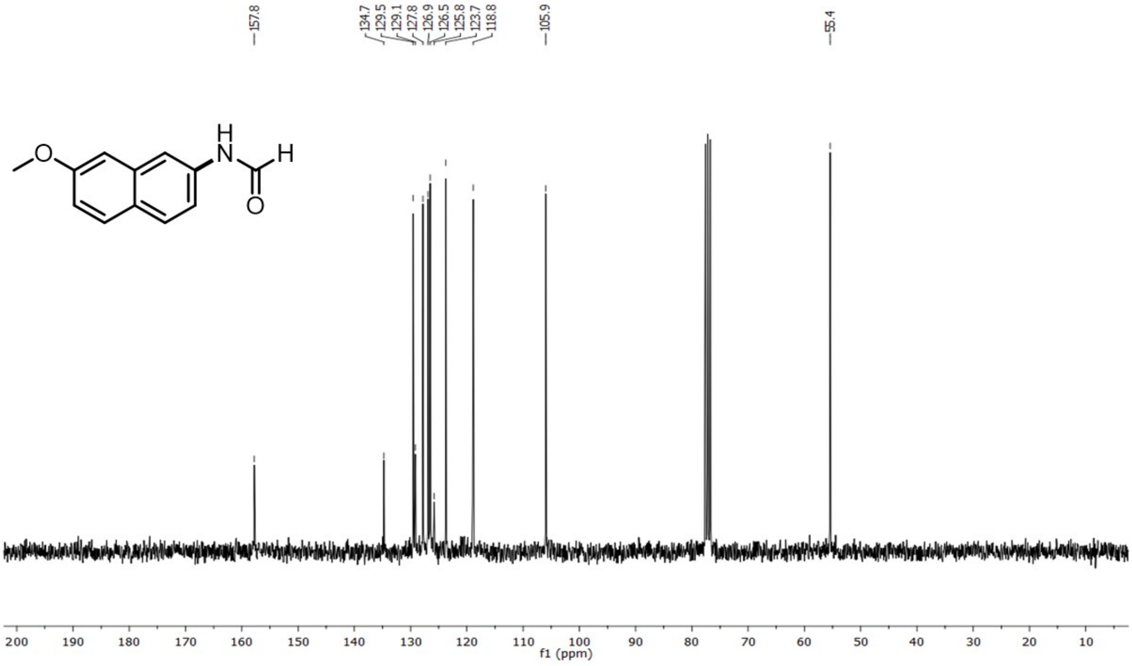

8. Computational details.

Geometry optimization of minima and transition states were carried out with the Gaussian 09 software package.[34] Optimizations were carried out without symmetry or geometry restrictions using the M06L functional with the double ζ 6-31g(d,p) basis set[35] on all elements except palladium, which was described with the SDD basis set and its associated electron core potential (ECP)[36]. The optimization and frequency calculations were performed including bulk solvent effects (ethanol) with the continuum SMD model.[37] The final energies were obtained at the DFT(M06) level with the triple ζ 6-311+g(2d,p) basis on all lighter elements, and palladium being described with the LANL2TZ(f) basis set and ECP, which accounts for the relativistic effects.[38] Vibrational frequencies were used to classify all stationary points as either minima (i.e. reactants, intermediates and products, with only real frequencies) or saddle points (i.e. transition states, with a single imaginary frequency vibrating along the reaction coordinate connecting reactants to products). The free energies reported include quasi-harmonic corrections for the entropy and concentration corrections (1 mol/L).[39]

8.1. Interactions between the Pd center and aryl rings of the terphenyl moiety.

The stabilization by non-covalent interactions between the Pd center and a side aryl ring of the terphenyl moiety was examined (Figure S1). Our calculations show that the active Pd(0) species is stabilized, ΔG = 11.7 kcal mol^{-1} in ethanol, through interactions with the carbons in the 1'- and 2'-positions of the biaryl group.

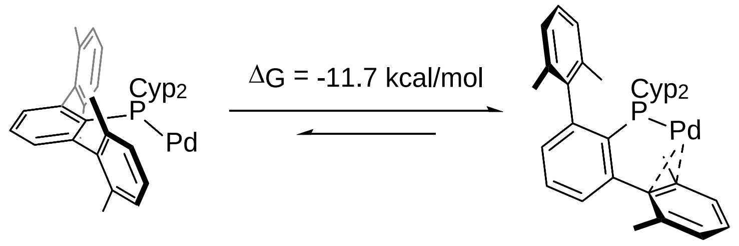

$PCyp_2Ar^{Xyl2}$_Mono $PCyp_2Ar^{Xyl2}$_Bi

Figure S1. Equilibria between monodentate and bidentate dialkylterphenyl phosphine Pd (0) species

8.2. Oxidative addition of phenyl sulfamate derivatives.

For the purpose of comparison, oxidative addition of phenyl sulfamate derivatives with different *para*-substituents (R = H, OH, CN) was also studied, see Figure S2. The formation of complexes **B** was exothermic regardless the substituents in the aryl moiety.

As expected, the energy barrier to oxidative addition is higher in the case of R = H and OH (27.7 and 28.3 kcal mol^{-1}), consistent with our experimental results.

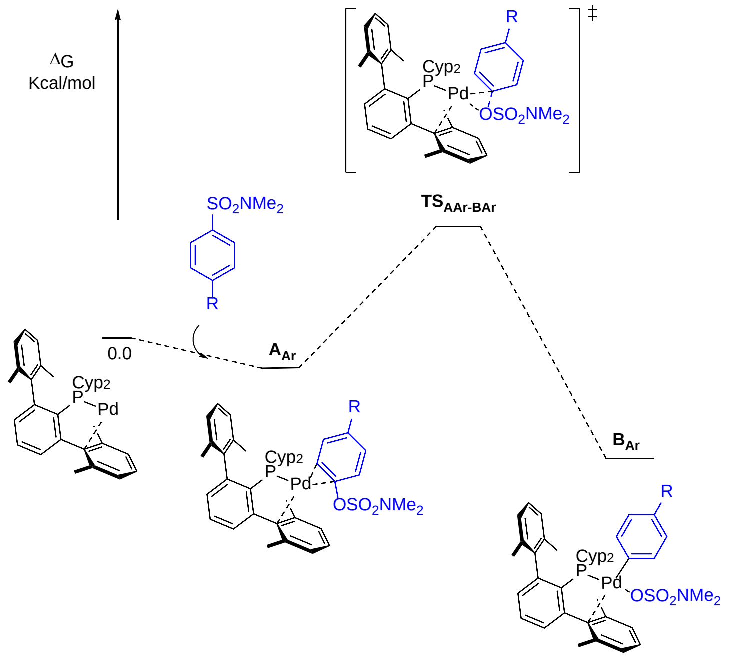

R	A_{Ar}	$TS_{AAr-BAr}$	B_{Ar}	$\Delta G^{‡}$
H	-1.4	26.3	-18.3	27.7
CN	-4.6	21.5	-20.7	26.1
OH	-0.4	27.9	-17.9	28.3

Figure S2. Gibbs energy profile, in kcal mol^{-1}, for the oxidative addition step of phenyl sulfamate derivatives.

8.3. Ligand exchange

Coordination of O*t*Bu$^-$ to the cationic complex **C** to give the square complex **E$_{OtBu}$** was also considered as an alternative to the path described in the main text (Figure S3). Likewise, an intramolecular deprotonation takes place through **TS$_{FOtBu-GOtBu}$** and, also similarly to that described in the main text. While the overall barrier for the formation of complex **H,** following dissociation of *t*BuOH from intermediate **G$_{OtBu}$** is 25.3 kcal mol^{-1}, the barrier via dissociation of O*t*Bu$^-$, coordination and intermolecular deprotonation of aniline is lower at 12.3 kcal mol^{-1}.

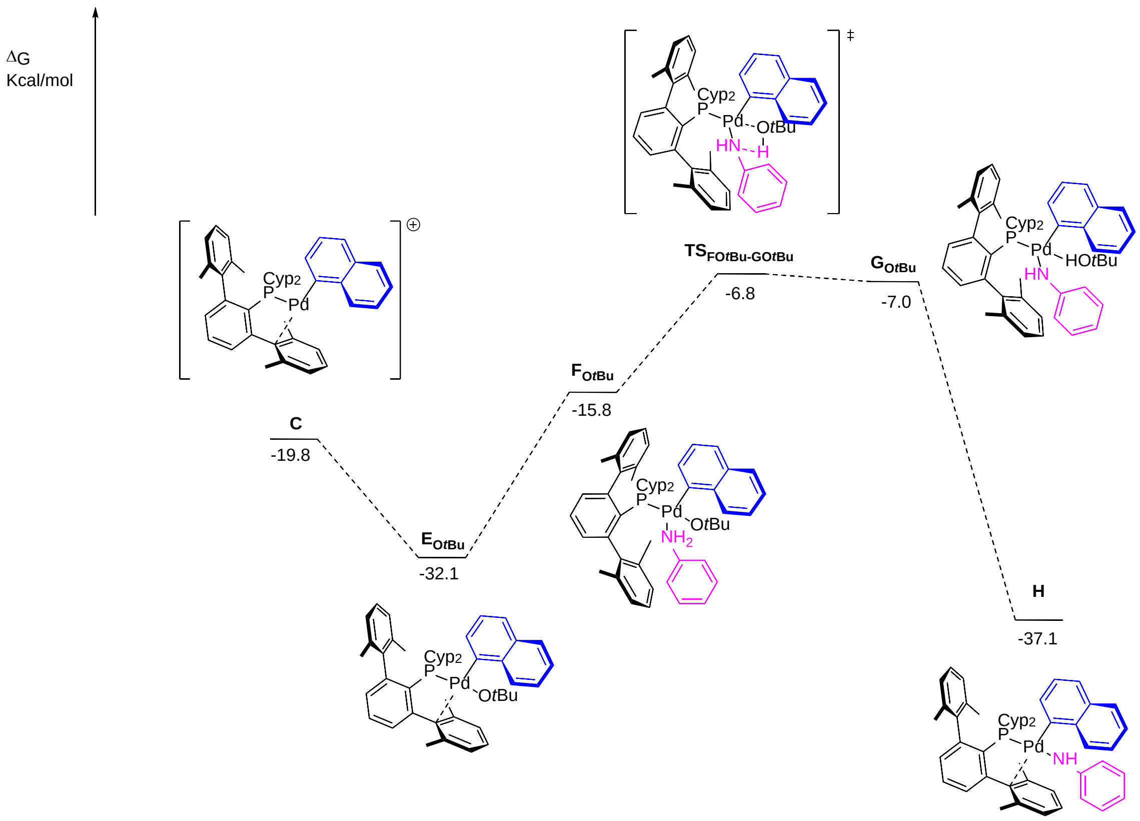

Figure S3. Gibbs energy profile for the formation of H via coordination of *t*BuO$^-$. Energies are in kcal mol^{-1}.

8.4. Mechanistic studies involving ethylamine

A calculated energy profile for the analogous reaction with ethylamine is shown in Figure S4. As noted for aniline, the first step is the coordination of the amine to cationic complex **C**. In this case a transition state was located for intermolecular deprotonation of ethylamine, **TS$_{FEtNH2\text{-}GEtNH2}$**, corresponding to a 2.6 kcal mol^{-1} from intermediate **E$_{EtNH2}$.** Notice that in this case **F$_{EtNH2}$** does not feature a metal to ligand interaction between the palladium and the OH$^-$. Rather, the latter forms a "soft complex" or an ionic pair with **E$_{EtNH2}$**. Subsequent release of H_2O would give rise to the anilido complex **H$_{EtNH2}$**. In the reductive elimination stage, the calculated transition state **TS$_{HEtNH2\text{-}IEtNH2}$** was 6.9 kcal·mol^{-1} higher in energy than intermediate **H$_{EtNH2}$**. The new complex **I$_{EtNH2}$** features, similarly to its aniline counterpart, the new amine coordinated to the Pd center through its N atom.

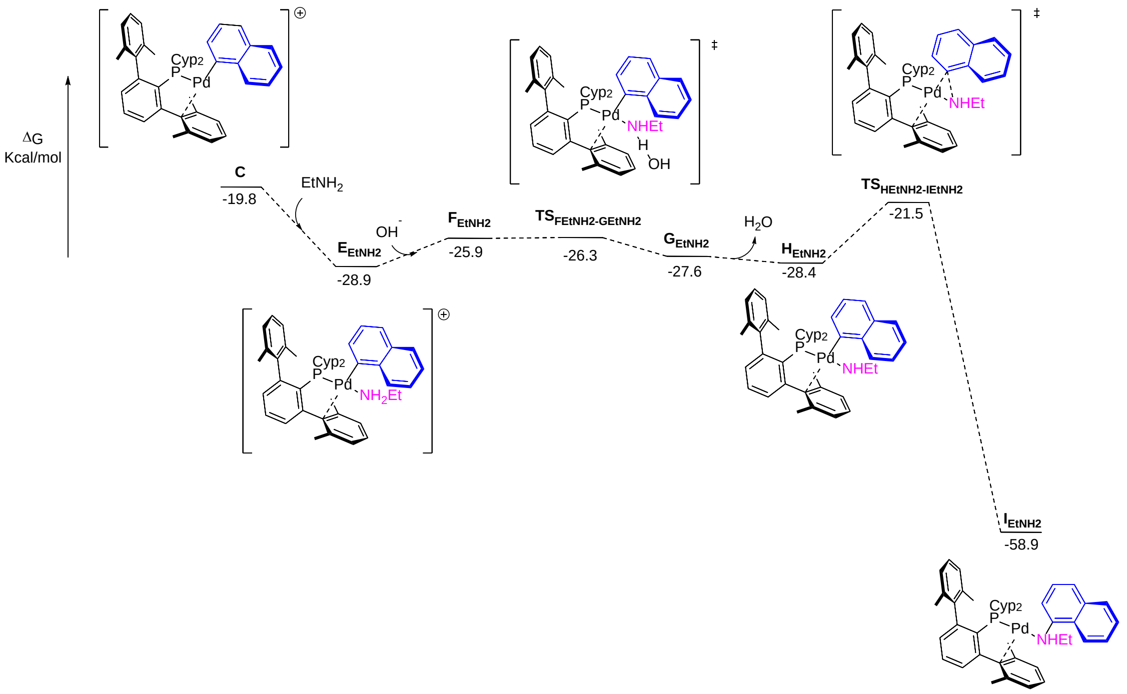

Figure S4. Gibbs energy profile for the coordination of ethylamine and reductive elimination. Energies are in kcal mol^{-1}

9. Microkinetic model

The reaction mechanism derived from the calculations was probed by utilizing a microkinetic model using the COPASI v. 4.39 software.[40] Table S5 summarizes the elementary steps and associated free energy barriers used to estimate the reaction rates. Ligand dissociation and ligand substitution reactions were assumed to be diffusion controlled and therefore having low energy barriers, with $\Delta G^‡$ being set to 4 kcal mol^{-1} in the chemical model for these reactions. Initial concentrations were taken from the experimental conditions for the catalysis: 1-naphthyl sulfamate, 1M; aniline, 1.2M. Concentration of *t*BuOH was set to 10.53 M (1 mL of *t*BuOH in 1 mL of H_2O). Initial concentrations of OH^-, *t*BuO^- and $PhNH^-$, were estimated from the pK_a values for *t*BuOH and $PhNH_2$,[41] considering the initial concentration of *t*BuOK (1.2 M), and assuming [H_2O] = 55.6 M. The microkinetic simulations were based on deterministic time course simulations with the LSODA algorithm.[42] The simulations were set to run for 18 h (68000 s) at 318 K and 383 K.

Table S5. Elementary steps and associated forward and reverse energy barriers-

Reaction (elementary step)	Forward $\Delta G^{‡,a}$	Reverse $\Delta G^{‡,a}$
1. cat + naphtylsulfamate $\rightleftharpoons$ **A**; k_1, k_{-1}	4.0	12.3
2. **A** $\longrightarrow$ **B**; k_2 (from **TS_{A-B}**); oxidative addition	27.3 (24.8)[b]	43.8
3. **B** $\rightleftharpoons$ **C** + sulfamate; k_3, k_{-3}	4.0	4.0
4. **C** + Aniline $\rightleftharpoons$ **E_{PhNH2}**; k_4, k_{-4}	4.0	4.0
5. **C** + H_2O $\rightleftharpoons$ **E_{H2O}**; k_5, k_{-5}	7.2	4.0
6. **C** + OH^- $\rightleftharpoons$ **E_{OH}**; k_5, k_{-5}	4.0	21.2
7. **C** + *t*BuO$^-$ $\rightleftharpoons$ **E_{OtBu}**; k_6, k_{-6}	4	16.3
8. **C** + $PhNH^-$ $\rightleftharpoons$ **E_{PhNH}**; k_7, k_{-7}	4	21.3
9. **E_{OH}** + Aniline $\rightleftharpoons$ **F_{OH}**; k_8, k_{-8}	20.9	4
10. **E_{OtBu}** + Aniline $\rightleftharpoons$ **F_{OtBu}**; k_9, k_{-9}	16.3	4
11. **F_{OH}** $\rightleftharpoons$ **G_{OH}**; k_{10}, k_{-10} (from **$TS_{FOH-GOH}$**)	11.7	0.9
12. **F_{OtBu}** $\rightleftharpoons$ **G_{OtBu}**; k_{11}, k_{-11} (from **$TS_{FOtBu-GOtBu}$**)	9.0	0.2
13. **G_{OH}** $\rightleftharpoons$ **H** + H_2O; k_{12}, k_{-12}	4	31.8
14. **G_{OtBu}** $\rightleftharpoons$ **H** + *t*BuOH; k_{13}, k_{-13}	4	34.1
15. **E_{PhNH2}** + OH^- $\rightleftharpoons$ **H** + H_2O; k_{14}, k_{-14}	4	21.3
16. **H** $\rightleftharpoons$ **I**; k_{15}, k_{-15} (from **TS_{H-I}**); reductive coupling	11.9	27.2
17. **I** $\rightleftharpoons$ **naphthlyamine + cat**; k_{16}, k_{-1}	4	6.2

[a]kcal mol^{-1}; [b]estimated from experimental observations.

The results from the simulations indicated very low conversions even at 383 K, suggesting that the barrier for the oxidative addition step was overestimated in our DFT calculations. Setting the forward energy barrier for this step to 24.6 kcal·mol^{-1}, ca. 2.5 kcal mol^{-1} below the calculated, yielded 100% conversion at times ≈ 65000 s, which would agree with the experimental reaction times and conversions. Moreover, a new chemical model was designed so that the outcome of the different routes could be differentiated in the results of the microkinetic simulations. This was easily achieved by giving different names to the intermediates and products of the various routes in the

program setup. In the new simulations, the concentrations of the C-N coupling product obtained via intermolecular deprotonation of $\mathbf{E_{PhNH2}}$ by OH^- was several orders of magnitude higher than that from the next more productive route, that taking place via $\mathbf{E_{OH}}$ (Figure S5).

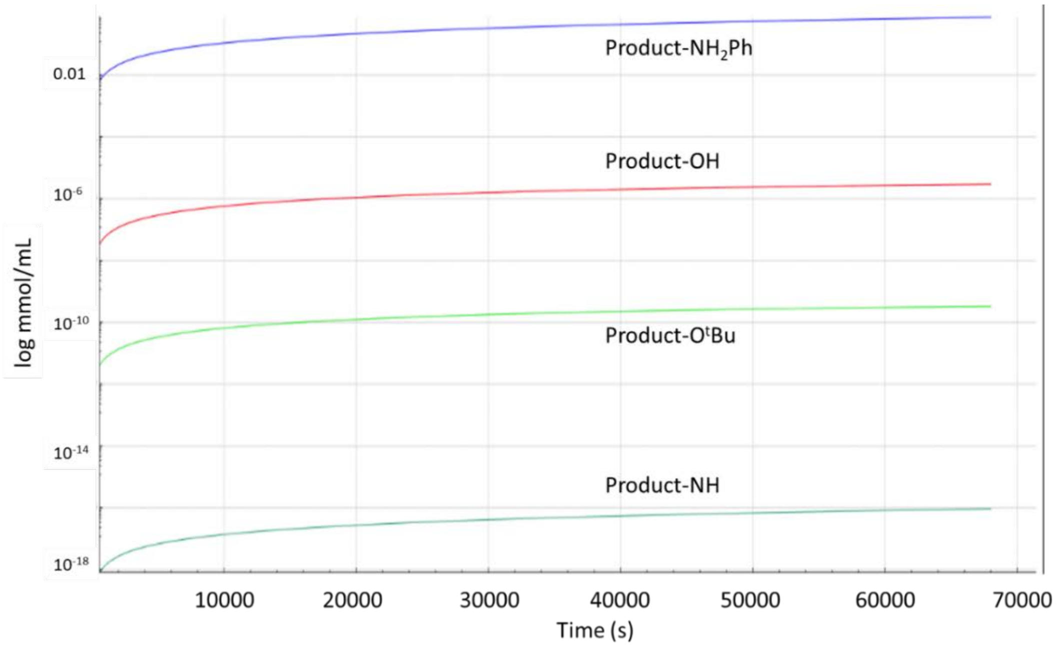

Figure S5. Time course of the concentration of the C-N coupling product decomposed as contributions from the calculated routes that involve coordination of $PhNH_2$, OH^-, *t*BuO^-, and $PhNH^-$ to **C**. The vertical scale is logarithmic.

Figure S6 indeed represents the calculated concentration of several palladium species formed during catalysis, the most abundant of which is $\mathbf{E_{OH}}$. But even this species is present in a roughly constant concentration of ca. $5{\cdot}10^{-4}$ M over the course of the reaction, according to our microkinetic analysis. This is so since formation of $\mathbf{E_{OH}}$ is reversible. It does not evolve through $\mathbf{TS_{FOH\text{-}GOH}}$, but can dissociate OH- to regenerate **C**, and **C** can in turn coordinate to the amine to afford **H**, as shown in Figure 5. The overall barrier for this process is 17.2 kcal/mol. Figure 6 also shows how, towards the end of the reaction, when the substrates are almost consumed, $\mathbf{E_{OH}}$ does not accumulate. Instead, it reacts through **C** with the remaining amine to form **H** and eventually complex **I**, whose formation is not reversible. Providing no aryl sulfamate remains in the reaction medium either, to turn over the catalytic cycle, species **I** should be the only remaining Pd species at the end of the catalysis.

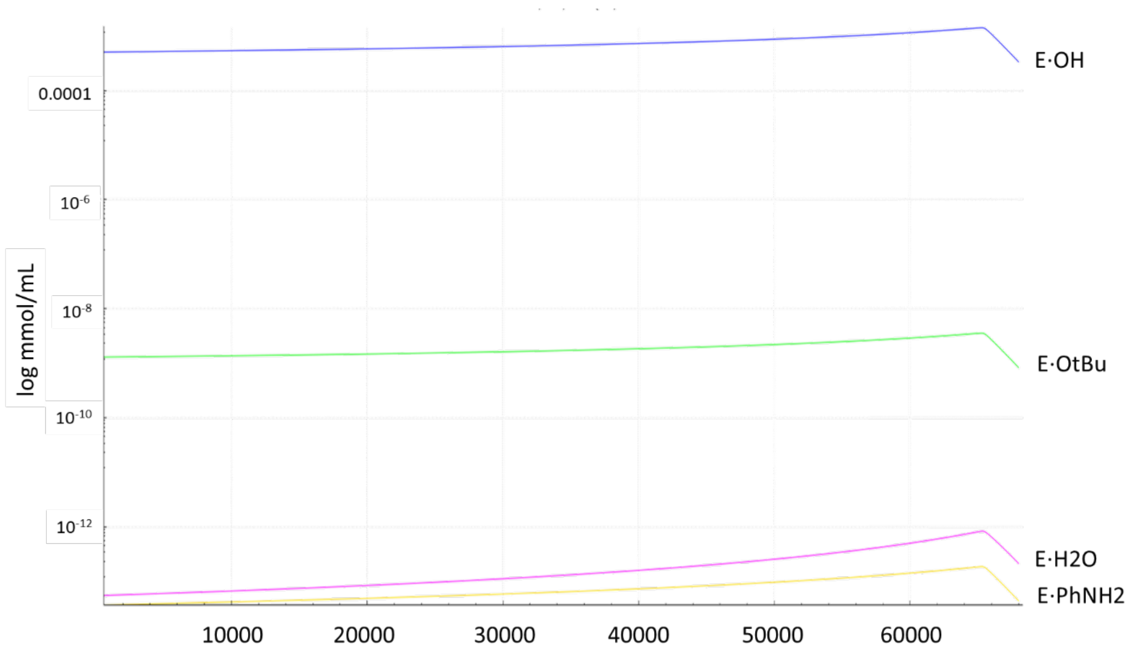

Figure S6. Time course of the concentration of intermediates E. The concentration of **E·PhNH2** is the lowest throughout the reaction course since it evolves faster into the product. The vertical scale is logarithmic.

10. References

1. Marín, M.; Moreno, J. J.; Navarro-Gilabert, C.; Álvarez, E.; Maya, C.; Peloso, R.; Nicasio, M. C.; Carmona, E. Synthesis, Structure and Nickel Carbonyl Complexes of Dialkylterphenyl Phosphines. *Chem. Eur. J.* **2019**, *25*, 260-272.

2. (a) Monti, A.; Rama, R. J.; Gomez, B.; Maya, C.; Alvarez, E.; Carmona, E.; Nicasio, M. C. *N*-substituted aminobiphenyl palladacycles stabilized by dialkylterphenyl phosphanes: Preparation and applications in C–N cross-coupling reactions. *Inorg. Chim. Acta* **2021**, *518*, 120214. (b) Rama, R. J.; Maya, C.; Nicasio, M. C. Dialkylterphenyl Phosphine-Based Palladium Precatalysts for Efficient Aryl Amination of *N*-Nucleophiles. *Chem. Eur. J.* **2020**, *26*, 1064-1073. (c) Bruno, N. C.; Tudge, M. T.; Buchwald, S. L. Design and preparation of new palladium precatalysts for C–C and C–N cross-coupling reactions. *Chem. Sci.* **2013**, *4*, 916-920. (d) Bruno, N. C.; Niljianskul, N.; Buchwald, S. L. N-Substituted 2-Aminobiphenylpalladium Methanesulfonate Precatalysts and Their Use in C–C and C–N Cross-Couplings. *J. Org. Chem.* **2014**, *79*, 4161-4166.

3. (a) Quasdorf, K. W.; Riener, M.; Petrova, K. V.; Garg, N. K. Suzuki−Miyaura Coupling of Aryl Carbamates, Carbonates, and Sulfamates. *J. Am. Chem. Soc.* **2009**, *131*, 17748-17749. (b) Quasdorf, K. W.; Antoft-Finch, A.; Liu, P.; Silberstein, A. L.; Komaromi, A.; Blackburn, T.; Ramgren, S. D.; Houk, K. N.; Snieckus, V.; Garg, N. K. Suzuki−Miyaura Cross-Coupling of Aryl Carbamates and Sulfamates: Experimental and Computational Studies. *J. Am. Chem. Soc.* **2011**, *133*, 6352-6363.

4. Odedra, A.; Wu, C.-J.; Pratap, T. B.; Huang, C.-W.; Ran, Y.-F.; Liu, R.-S. Ruthenium-Catalyzed Aromatization of Enediynes via Highly Regioselective Nucleophilic Additions on a π-Alkyne Functionality. A Useful Method for the Synthesis of Functionalized Benzene Derivatives. *J. Am. Chem. Soc.* **2005**, *127*, 3406-3412.

5. Desmarets, C.; Champagne, B.; Walcarius, A.; Bellouard, C.; Amrani, R. O.; Ahajji, A.; Fort, Y.; Schneider, R. Facile Synthesis and Characterization of Naphthidines as a New Class of Highly Nonplanar Electron Donors Giving Robust Radical Cations. *J. Org. Chem*. **2006**, *71*, 1351-1361.

6. (a) Gao, C.-Y.; Yang, L.-M. Nickel-Catalyzed Amination of Aryl Tosylates. *J. Org. Chem*. **2008**, *73*, 1624-1627. (b) Ogata, T.; Hartwig, J. F. Palladium-Catalyzed Amination of Aryl and Heteroaryl Tosylates at Room Temperature *J. Am. Chem. Soc.* **2008**, *130*, 13848-13849.

7. Dumrath, A.; Lübbe, C.; Neumann, H.; Jackstell, R.; Beller, B. Recyclable Catalysts for Palladium-Catalyzed Aminations of Aryl Halides. *Chem. Eur. J.* **2011**, *17*, 9599-9604.

8. Raders, S. M.; Moore, J. N.; Parks, J. K.; Miller, A. D.; Leissing, T. M.; Kelley, S. P.; Rogers, R. D.; Shaughnessy, K. H. Trineopentylphosphine: A Conformationally Flexible Ligand for the Coupling of Sterically Demanding Substrates in the Buchwald–Hartwig Amination and Suzuki–Miyaura Reaction. *J. Org. Chem.* **2013***, 78,* 4649-4664.

9. Jong, H.; Eey, S. T.-C.; Lim, Y. H.; Pandey, S.; Iqbal, N. A. B.; Yong, F. F.; Robins, E. G.; Johannes, C. W. One-Pot Palladium-Catalyzed Cross-Coupling Treble of Borylation, the Suzuki Reaction and Amination. *Adv. Synth, Catal.* **2017***, 359,* 616-622.

10.Hajra, A.; Wei, Y.; Yoshikai, N. Palladium-Catalyzed Aerobic Dehydrogenative Aromatization of Cyclohexanone Imines to Arylamines. *Org. Lett.* **2012***, 14,* 5488-5491.

11 Chen, W.; Chen, K.; Chen, W.; Liu, M.; Wu, H. Well-Designed N-Heterocyclic Carbene Ligands for Palladium-Catalyzed Denitrative C–N Coupling of Nitroarenes with Amines. *ACS Catal.* **2019**, *9*, 8110-8115.

12. Kim, M.; Chang, S. Rhodium(NHC)-Catalyzed Amination of Aryl Bromides. *Org. Lett.* **2010**, *12*, 1640-1643.

13. Mishra, A. K.; Biswas, S. Brønsted Acid Catalyzed Functionalization of Aromatic Alcohols through Nucleophilic Substitution of Hydroxyl Group. *J. Org. Chem*. **2016**, *81*, 2355–2363.

14. Kataoka, N.; Shelby, Q.; Stambuli, J. P.; Hartwig, J. F. Air Stable, Sterically Hindered Ferrocenyl Dialkylphosphines for Palladium-Catalyzed C−C, C−N, and C−O Bond-Forming Cross-Couplings. *J. Org. Chem.* **2002**, *67, 5553*-5566.

15. Kuwano, R.; Utsunomiya, M.; Hartwig, J. F. Aqueous Hydroxide as a Base for Palladium-Catalyzed Amination of Aryl Chlorides and Bromides. *J. Org. Chem.* **2002**, *67*, 6479-6486.

16. Richardson, J.; Ruble, J. C.; Love, E. A.; Berritt, S. A Method for Identifying and Developing Functional Group Tolerant Catalytic Reactions: Application to the Buchwald–Hartwig Amination. *J. Org. Chem.* **2017***, 82,* 3741-3750.

17. Meyers, C.; Maes, B. U. W.; Loones, K. T. J.; Bal, G.; Lemière, G. L. F.; Dommisse, R. A. Study of a New Rate Increasing "Base Effect" in the Palladium-Catalyzed Amination of Aryl Iodides. *J. Org. Chem.* **2004**, *69*, 6010-6017.

18. Huang, J. H.; Yang, L. M. Nickel-Catalyzed Amination of Aryl Phosphates through Cleaving Aryl C–O Bonds. *Org. Lett.* **2011**, *13,* 3750-3753.

19. Cortright, S. B.; Huffman, J. C.; Yoder, R. A.; Coalter III, J. N.; Johnston, J. N. IAN Amines: Chiral C2-Symmetric Zirconium(IV) Complexes from Readily Modified Axially Chiral C1-Symmetric β-Diketimines. *Organometallics* **2004**, *23,* 2238-2250.

20. Wolfe, J. P.; Buchwald, S. L. Scope and Limitations of the Pd/BINAP-Catalyzed Amination of Aryl Bromides. *J. Org. Chem.* **2000**, *65*, 1144-1157.

21. Driver, M. S.; Hartwig, J. F. A Second-Generation Catalyst for Aryl Halide Amination: Mixed Secondary Amines from Aryl Halides and Primary Amines Catalyzed by (DPPF)$PdCl_2$. *J. Am. Chem. Soc.* **1996**, *118,* 7217-7218.

22. Ackermann, L.; Sandmann, R.; Song, W. Palladium- and Nickel-Catalyzed Aminations of Aryl Imidazolylsulfonates and Sulfamates. *Org. Lett.* **2011**, *13,* 1784-1786.

23. Huang, Y.-B.; Yang, C.; Yi, J.; Deng, X.-J.; Fu, Y.; Liu, L. Cu-Catalyzed Carbon-Heteroatom Coupling Reactions under Mild Conditions Promoted by Resin-Bound Organic Ionic Bases. *J. Org. Chem.* **2011***, 76,* 800-810.

24. Shen, Q.; Ogata, T.; Hartwig, J. F. Highly Reactive, General and Long-Lived Catalysts for Palladium-Catalyzed Amination of Heteroaryl and Aryl Chlorides, Bromides, and Iodides: Scope and Structure–Activity Relationships. *J. Am. Chem. Soc.* **2008***, 130,* 6586-6596.

25. Ackermann, L.; Lygin, A. V. Cationic Ruthenium(II) Catalysts for Oxidative C–H/N–H Bond Functionalizations of Anilines with Removable Directing Group: Synthesis of Indoles in Water. *Org. Lett.* **2012**, *14,* 764-767.

26. Andreson, K. W.; Tundel, R. E.; Ikawa, T.; R. Altman, A.; Buchwald, S. L. Monodentate Phosphines Provide Highly Active Catalysts for Pd-Catalyzed C-N Bond-Forming Reactions of Heteroaromatic Halides/Amines and (H)N-Heterocycles. *Angew. Chem. Int. Ed.* **2006**, *45*, 6523-6527.

27. (a) Kouznetsov, V. V.; Zacchino, S. A.; Sortino, M.; Vargas Mendez, L. Y.; Gupta, M. P. Cytotoxic and Antifungal Activities of Diverse α-Naphthylamine Derivatives. *Sci. Pharm.* **2012**, *80,* 867-878. (b) Pistritto, V. A.; Schutzbach-Horton, M. E.; Nicewicz, D. A. Nucleophilic Aromatic Substitution of Unactivated Fluoroarenes Enabled by Organic Photoredox Catalysis. *J. Am. Chem. Soc.* **2020**, *142,* 17187-17194.

28. (a) Majumdar, K. C.; Chakravorty, S.; Taher, A. New Efficient RCM-Mediated Synthesis of Pyrrolidine Derivatives. *Synth. Commun*. **2008**, *38*, 3159-3169. (b) Li, X.; Yang, D.; Jiang, Y.; Fu, H. Efficient copper-catalyzed N-arylations of nitrogen-containing heterocycles and aliphatic amines in water. *Green Chem*. **2010**, *12*, 1097-1105.

29. Liu, X.; Sheng, H.; Zhou, Y.; Song, Q. Palladium-catalyzed C–H bond activation for the assembly of N-aryl carbazoles with aromatic amines as nitrogen sources. *Chem. Commun.* **2020**, *56*, 1665-1668.

30. So, C. M.; Zhou, Z.; Lau, C. P.; Kwong, F. Y. Palladium-Catalyzed Amination of Aryl Mesylates. *Angew. Chem. Int. Ed*. **2008**, *47*, 6402-6406.

31. Al-Awadi, H.; Ibrahim, M. R.; Dib, H. H.; Al-Awadi, N. A.; Ibrahim, Y. A. Gas-phase thermolysis of 1-acylnaphtho[1,8-de][1,2,3]triazines. Interesting direct routes towards condensed naphtho[1,8-de]heterocyclic ring systems. *Tetrahedron* **2005**, *61*, 10507-10513.

32. Itoh, N.; Sakamoto, T.; Miyazawa E.; Kikugawa, Y. Introduction of a Hydroxy Group at the Para Position and N-Iodophenylation of N-Arylamides Using Phenyliodine(III) Bis(Trifluoroacetate). *J. Org. Chem.* **2002***, 67*, 7424-7428.

33. Rama, R. J.; Maya,C.; Nicasio, M. C. Palladium-mediated intramolecular dearomatization of ligated dialkylterphenyl phosphines. *Dalton Trans.* **2019***, 48,* 14575-14579.

34. Gaussian 09, Revision E.01 and B.01, Frisch, M. J.; Trucks, G. W.; Schlegel, H. B.; Scuseria, G. E.; Robb, M. A.; Cheeseman, J. R.; Scalmani, G.; Barone, V.; Mennucci, B.; Petersson, G. A.; Nakatsuji, H.; Caricato, M.; Li, X.; Hratchian, H. P.; Izmaylov, A. F.; Bloino, J.; Zheng, G.; Sonnenberg, J. L.; Hada, M.; Ehara, M.; Toyota, K.; Fukuda, R.; Hasegawa, J.; Ishida, M.; Nakajima, T.; Honda, Y.; Kitao, O.; Nakai, H.; Vreven, T.; Montgomery, J. A., Jr.; Peralta, J. E.; Ogliaro, F.; Bearpark, M. J.; Heyd, J. J.; Brothers, E. N.; Kudin, K. N.; Staroverov, V. N.; Keith, T. A.; Kobayashi, R.; Normand, J.; Raghavachari, K.; Rendell, A. P.; Burant, J. C.; Iyengar, S. S.; Tomasi, J.; Cossi, M.; Rega, N.; Millam, J. M.; Klene, M.; Knox, J. E.; Cross, J. B.; Bakken, V.; Adamo, C.; Jaramillo, J.; Gomperts, R.; Stratmann, R. E.; Yazyev, O.; Austin, A. J.; Cammi, R.; Pomelli, C.; Ochterski, J. W.; Martin, R. L.; Morokuma, K.; Zakrzewski, V. G.; Voth, G. A.; Salvador, P.; Dannenberg, J. J.; Dapprich, S.; Daniels, A. D.; Farkas, O.; Foresman, J. B.; Ortiz, J. V.; Cioslowski, J.; Fox, D. J. Gaussian, Inc., Wallingford CT, 2010.

35. (a) Ditchfield, R.; Hehre, W. J.; Pople J. Self-Consistent Molecular-Orbital Methods. IX. An Extended Gaussian-Type Basis for Molecular-Orbital Studies of Organic Molecules. *J. Chem. Phys.* **1971**, *54*, 724–728. b) Hehre, W. J.; Ditchfield, R.; Pople. J. A. Self-Consistent Molecular Orbital Methods. XII. Further Extensions of Gaussian-Type Basis Sets for Use in Molecular Orbital Studies of Organic Molecules. *J. Chem. Phys.* **1972**, *56*, 2257–2261. c) Hariharan, P. C.; Pople, J. A. The influence of polarization functions on molecular orbital hydrogenation energies. *Theor. Chim. Acta* **1973**, *28*, 213–222. d) Francl, M. M.; Pietro, W. J.; Hehre, W. J.; Binkley, J. S.; Gordon, M. S.; DeFrees, D. J.; Pople, J. A. Self-Consistent Molecular Orbital Methods. XXIII. A Polarization-Type Basis Set for Second-Row Elements. *J. Chem. Phys.* **1982**, *77*, 3654–3665.

36. Andrae, D.; Haeussermann, U.; Dolg, M.; Stoll, H.; Preuss, H. Energy-adjusted ab initio pseudopotentials for the 2nd and 3rd row transition-elements. *Theor. Chem. Acc.*, **1990**, *77*, 123-41.

37. Marenich, A. V.; Cramer, C. J.; Truhlar. D. G. Universal Solvation Model Based on Solute Electron Density and on a Continuum Model of the Solvent Defined by the Bulk Dielectric Constant and Atomic Surface Tensions. *J. Phys. Chem. B* **2009**, *113*, 6378–6396.

38. Hay, P. J.; Wadt, W. R. Ab initio effective core potentials for molecular calculations. Potentials for K to Au including the outermost core orbitals. *J. Chem. Phys.* **1985**, *82*, 299-310.

39. (a) Normal vibration modes with v ≤ 100 cm-1 were obtained from the free-rotor approximation. Grimme, S. Supramolecular Binding Thermodynamics by Dispersion-Corrected Density Functional Theory. *Chem. Eur. J.* **2012**, *18*, 9955–9964. (b) Quasi harmonic corrections have been performed with the GoodVibes software: Luchini, G.; Alegre-Requena, J. V.; Funes-Ardoiz, I.; Paton, R. S. *F1000Research*, **2020**, *9*, 291.

40. COPASI v 4.36; Hoops, S.; Sahle, S.; Gauges, R.; Lee, C.; Pahle, J.; Simus, N.; Singhal, M.; Xu, L.; Mendes, P.; Kummer, U. COPASI--a COmplex PAthway SImulator.

Bioinformatics **2006**, *22* (24), 3067–3074. https://doi.org/10.1093/bioinformatics/btl485.

41. Ripin, D. H.; Evans, D. A. p*Ka* Table. https://organicchemistrydata.org/hansreich/resources/pka/pka_data/evans_pKa_table.pdf (updated 4/8/2022).

42. Petzpold, L. Automatic Selection of Methods for Solving Stiff and Nonstiff Systems of Ordinary Differential Equations. SIAM *J. Sci. Stat. Comput.* **1983**, *4*, 136–148.
